# Acidotolerant *Bacteria* and Fungi as a Sink of Methanol-Derived Carbon in a Deciduous Forest Soil

**DOI:** 10.3389/fmicb.2017.01361

**Published:** 2017-07-24

**Authors:** Mareen Morawe, Henrike Hoeke, Dirk K. Wissenbach, Guillaume Lentendu, Tesfaye Wubet, Eileen Kröber, Steffen Kolb

**Affiliations:** ^1^Department of Ecological Microbiology, University of Bayreuth Bayreuth, Germany; ^2^Department of Molecular Systems Biology, Helmholtz Centre for Environmental Research Leipzig, Germany; ^3^Department of Pharmaceutical and Medicinal Chemistry, Institute of Pharmacy, University of Leipzig Leipzig, Germany; ^4^Institute of Forensic Medicine, University Hospital Jena Jena, Germany; ^5^Department of Ecology, University of Kaiserslautern Kaiserslautern, Germany; ^6^Department of Soil Ecology, Helmholtz Centre for Environmental Research Leipzig, Germany; ^7^Institute of Landscape Biogeochemistry, Leibniz Centre for Landscape Research Müncheberg, Germany

**Keywords:** DNA stable isotope probing, *mxaF*, bacterial 16S rRNA gene, fungal ITS, pH, high-throughput sequencing

## Abstract

Methanol is an abundant atmospheric volatile organic compound that is released from both living and decaying plant material. In forest and other aerated soils, methanol can be consumed by methanol-utilizing microorganisms that constitute a known terrestrial sink. However, the environmental factors that drive the biodiversity of such methanol-utilizers have been hardly resolved. Soil-derived isolates of methanol-utilizers can also often assimilate multicarbon compounds as alternative substrates. Here, we conducted a comparative DNA stable isotope probing experiment under methylotrophic (only [^13^C_1_]-methanol was supplemented) and combined substrate conditions ([^12^C_1_]-methanol and alternative multi-carbon [^13^C_u_]-substrates were simultaneously supplemented) to (i) identify methanol-utilizing microorganisms of a deciduous forest soil (European beech dominated temperate forest in Germany), (ii) assess their substrate range in the soil environment, and (iii) evaluate their trophic links to other soil microorganisms. The applied multi-carbon substrates represented typical intermediates of organic matter degradation, such as acetate, plant-derived sugars (xylose and glucose), and a lignin-derived aromatic compound (vanillic acid). An experimentally induced pH shift was associated with substantial changes of the diversity of active methanol-utilizers suggesting that soil pH was a niche-defining factor of these microorganisms. The main bacterial methanol-utilizers were members of the *Beijerinckiaceae* (*Bacteria*) that played a central role in a detected methanol-based food web. A clear preference for methanol or multi-carbon substrates as carbon source of different *Beijerinckiaceae*-affiliated phylotypes was observed suggesting a restricted substrate range of the methylotrophic representatives. Apart from *Bacteria*, we also identified the yeasts *Cryptococcus* and *Trichosporon* as methanol-derived carbon-utilizing fungi suggesting that further research is needed to exclude or prove methylotrophy of these fungi.

## Introduction

Methanol is an abundant volatile organic compound (VOC) in the troposphere, i.e., reaches mixing ratios of up to 10 ppb. Due to its high reactivity, methanol impacts on the oxidative capacity, the formation of ozone, and pools of important organic reactants in the atmosphere (Galbally and Kirstine, 2002; Wohlfahrt et al., 2015). Atmospheric methanol mainly originates from growing plants and decaying plant material but originates to a smaller extent from reactions of methyl peroxy radicals in the troposphere (Fall and Benson, 1996; Warneke et al., 1999; Galbally and Kirstine, 2002; Millet et al., 2008; Wohlfahrt et al., 2015). Large sinks for atmospheric methanol are reactions with hydroxyl radicals and oceanic uptake, including contributions of microorganisms (Millet et al., 2008). The role of methylotrophic microorganisms in terrestrial ecosystems as global sinks of methanol is nonetheless undisputed (Kolb, 2009; Stacheter et al., 2013; Wohlfahrt et al., 2015). However, their environmental controls, their distribution in the phyllo- and rhizosphere, and their diversity in different climate zones are largely unresolved (Kolb, 2009; Kolb and Stacheter, 2013; Stacheter et al., 2013; Wohlfahrt et al., 2015).

Microbial methanol-utilizers were discovered in the late 19th century and can be aerobic or anaerobic (Loew, 1892; Chistoserdova et al., 2009; Kolb, 2009; Chistoserdova and Lidstrom, 2013; Hedderich and Withman, 2013). Aerobic methanol-utilizers are phylogenetically diverse and affiliate with gram-negative *Alpha*-, *Beta*-, and *Gammaproteobacteria* and *Verrucomicrobia* and with gram-positive *Actinobacteria* and *Firmicutes*, as well as with some fungi, i.e., ascomycetous yeasts and molds (Kolb, 2009; Gvozdev et al., 2012; Chistoserdova and Lidstrom, 2013; Kolb and Stacheter, 2013; Sharp et al., 2014).

Although methanol-utilizers were among the first microorganisms that were targeted in the environment using molecular tools two decades ago, an understanidng of their global biogeography has only just started to grow (Holmes et al., 1995; Kolb and Stacheter, 2013). The sensitivity of the environmental detection of low-abundant, one-carbon (C_1_) compound converting microorganisms has been improved by the detection of key genes of methylotrophy (Holmes et al., 1995; McDonald and Murrell, 1997; Kolb and Stacheter, 2013). The initial enzymatic step of microbial methanol utilization is the oxidation of methanol to formaldehyde. For this reaction, at least three different enzymes occur in *Bacteria* (Kolb and Stacheter, 2013). In methylotrophic fungi, methanol is oxidized by alternative oxidoreductases (Gvozdev et al., 2012). The most prominent methanol oxidoreductase is the pyrroloquinoline quinone (PQQ)-dependent methanol dehydrogenase (MDH) encoded by the genes *mxaFI*. *Burkholderiales* possess an isoenzyme encoded by *mdh2* (Kalyuzhnaya et al., 2008). In many *Bacteria, xoxF* (synonymous to *mxaF’*) is present, and a subtype (belonging to clade *xoxF4*) encodes a functional MDH that has been recently used to detect *xoxF*-possessing *Bacteria* in coastal environments (Chistoserdova, 2011; Taubert et al., 2015). Based on this broad spectrum of known enzymes, several gene markers have been developed. However, only *mxaF* and *xoxF* are currently well-established functional gene markers that have been used in environmental surveys of methanol-utilizers, whereby no universal primers covering all five *xoxF* clades exist (McDonald and Murrell, 1997; Moosvi et al., 2005; Neufeld et al., 2007; Stacheter et al., 2013; Taubert et al., 2015). Knowing the limitations of available functional markers, we complemented the detection of *mxaF/xoxF* and bacterial 16S rRNA genes in combination with a stable isotope labeling apporach to identify methanol-utilizers in a forest soil.

The taxonomic diversity of methanol-utilizers in temperate forest soils is affected by soil pH and the presence of trees, but a detailed understanding of driving factors in these ecosystems is lacking (Kolb, 2009; Degelmann et al., 2010; Stacheter et al., 2013). Soil-derived methanol-utilizers often grow on multi-carbon compounds (Kolb, 2009). Thus, one may speculate that such facultatively methylotrophic microorganisms might occupy different ecological niches with regard to their alternative substrates in the organic compound-rich top layer of forest soils, which would enable them to permanently establish in a complex soil community along with other methanol-utilizers and non-methylotrophic heterotrophs.

Few studies have begun to gain a more detailed understanding of methanol-utilizers in soils (Radajewski et al., 2000, 2002; Lueders et al., 2004; Stacheter et al., 2013). The limited molecular detection based on gene markers in most of the previous studies on soil methylotrophs might have led to an underestimation of the taxonomic biodiversity of these methylotrophs. Thus, the current scientific view on terrestrial methanol-utilizers is still largely based on pure cultures and few stable isotope probing (SIP) experiments (Radajewski et al., 2002; Lueders et al., 2004; Kolb, 2009).

In our study, comparative DNA SIP experiments with several ^13^C-isotopologs of potential alternative multi-carbon substrates and with [^13^C_1_]-methanol in separate treatments were conducted in an acidic aerated forest soil. Thereby, we focussed on the substrate utilization of methanol-utilizing methylotrophs under mixed substrate conditions (i.e., the presence of methanol and an alternative [^13^C_u_]-substrate). To better understand the role of soil pH for niche partitioning of detectable soil methanol-utilizers, we conducted a methanol-supplemented SIP experiment under the acidic *in situ* and artificially induced pH-neutral conditions, at which most soil-derived isolates have their growth optimum. The specific objectives of this study were (i) to identify aerobic bacteria and fungi that assimilated methanol-derived carbon, (ii) to evaluate the significance of soil pH on indigenous methanol-utilizers, and (iii) to resolve alternative substrate spectra of active methanol-utilizers using common soil organic compounds.

## Materials and Methods

### Study Site and Soil Sampling

The study site was located in a temperate German forest (Steigerwald, 49°52′ N, 10°28′ E) dominated by European beech (*Fagus sylvatica*). The characteristics of the soil are described elsewhere (Degelmann et al., 2009). Soil samples were taken from the upper part of the soil (10 cm, O plus A horizons) without the litter layer. Five sampling sites were chosen that reflected the general characteristics of the sampling area (saplings, dead wood, clearing, shady, and old beeches). Samples were taken in August 2013 (for the substrate SIP experiment) and in September 2014 (for the pH shift SIP experiment). Fresh forest soil samples were sieved (2 mm) and equally pooled to further prepare the soil slurries.

### [^13^C_u_]-Substrates for SIP Experiments

Filter-sterilized 40 mM stock solutions of methanol and multi-carbon substrates (i.e., acetate, glucose, xylose, and vanillic acid) were prepared with either the [^13^C]-isotopolog (‘labeled,’ 99 atom% C) or the [^12^C]-isotopolog (i.e., ‘unlabeled,’ natural abundance of ^13^C). All multi-carbon substrate stock solutions also included 40 mM [^12^C]-methanol. The isotopologs were fully labeled (i.e., [^13^C_u_]) except for vanillic acid, in which only the aromatic ring carbon atoms were [^13^C]-labeled (i.e., [^13^C_1-6_]). CO_2_ treatments were set up with either [^13^C]-CO_2_ (‘labeled,’ 99 atom% C; <3 atom% ^18^O) or [^12^C]-CO_2_. For more detailed information, refer to the Supplementary Information on Materials and Methods.

### Substrate SIP Experiment under Mixed Substrate Conditions

Soil slurries were prepared by mixing 50 g of freshly sieved soil with 40 ml of trace element solution (in 1 L of sterile water: HCl, 50 μM; FeCl_2_, 5 μM; ZnCl_2_, MnCl_2_, CoCl_2_, 50 μM; Na_2_MoO_4_, 0.15 μM; H_3_BO_3_, NiCl_2_, 0.10 μM; CuCl_2_, 0.01 μM; after Whittenbury et al., 1970) and initially homogenized by hand shaking. Incubations were conducted as soil slurries to achieve homogenous physicochemical conditions (e.g., substrate concentrations, redox conditions, and pH) and to provide a sufficent distribution of supplemented substrates as well as a balanced distribution of microorganisms to minimize heterogeneity in collected sub-samples.

Incubations were performed in duplicates for each approach (control, ^12^C, and ^13^C) on an end-over-end shaker at 20°C in the dark. Oxic conditions were maintained by placing a large gas phase inside the flasks (the ratio of gas phase to slurry volume was 12:1) and by daily opening for few minutes before re-sealing, allowing gas phase exchange. The O_2_ concentrations were monitored to ensure oxic conditions.

Substrates (i.e., methanol, acetate, and sugars; 1 ml) and methane were daily supplemented to a final concentration of 1 mM and 200 ppm, respectively. Vanillic acid (1 ml, 1 mM final concentration) was supplemented if it was no longer detected. In order to obtain mixed substrate conditions methanol (^12^C) was supplemented in combination with alternative multi-carbon substrates (^12^C or ^13^C) at the same concentrations (1 mM, final concentration). Thus, treatments solely supplemented with methanol served as multi-carbon substrate controls. Unsupplemented control treatments (i.e., solely 1 ml trace element solution was supplemented) served as methanol control treatments and lacked any other substrate supplementation besides methane. Methane was supplemented to support also methanotrophic organisms in the soil, which might also be important methanol-utilizers (such as the methanotrophic USCα group, Degelmann et al., 2010). CO_2_ incubations were supplemented with 10% CO_2_ in the headspace (approximately 7 mM total concentration) and opened if the O_2_ concentration was below 10%. The purpose of the CO_2_ treatments was to evaluate the cross-feeding effects through the assimilation of [^13^C]-CO_2_.

For more detailed information and an overview over the experimental set-up (Supplementary Figure S1), refer to the Supplementary Information on Materials and Methods.

### pH Shift SIP Experiment under Methylotrophic Conditions

Two treatments were conducted, mimicking acidic *in situ* and elevated pH-neutral conditions. Soil slurries were prepared according to the substrate SIP experiment. The treatments for *in situ* pH 4 were prepared by mixing 50 g freshly sieved soil and 40 ml of trace element solution in one incubation flask. The treatments for pH 7 were prepared by mixing 300 g freshly sieved soil with 240 ml of trace element solution. Then, the pH was adjusted to 7 with sterile NaOH, and the solution was mixed until the pH remained constant. A total of 90 ml of the pH adjusted slurry (corresponding with the volume of a slurry consisting of 50 g soil and 40 ml trace element solution) was placed into each incubation flask. Methanol was supplemented daily to a final concentration of 1 mM per pulse. Control treatments were only supplemented with the same volume of trace element solution. Daily aliquots were taken, and the pH was monitored to avoid changes and re-adjusted when necessary. The pH shift SIP experiment treatments lacked methane supplementation. Thus, a putatively supporting effect of methane on methylotrophs under *in situ* conditions could be evaluated by the comparison of all methanol treatments of both SIP experiments conducted, i.e., substrate SIP and pH shift SIP experiment. For more detailed information, refer to the Supplementary Information on Materials and Methods.

### Chemical Analytics

The pH value was determined in soil slurry aliquots. Gases were measured by gas chromatography using thermal conductivity (O_2_, CO_2_) and a flame ionization detector (methane). The amount of [^13^C]-CO_2_ was determined using GC mass spectrometry. The conversion of supplemented multi-carbon compounds was monitored by high-performance liquid chromatography (HPLC) using the refractive index and a diode array detector. Details can be found in the Supplementary Information on Materials and Methods.

### Nucleic Acid Extraction and Separation of ‘Heavy’ (H), ‘Middle’ (M), and ‘Light’ (L) DNA by Density Gradient Centrifugation

Nucleic acids were extracted from two 0.5 g soil slurry samples of each replicate according to Griffiths et al. (2000). DNA was precipitated, purified from co-extracted RNA by RNase treatment and quantified with Quant-iT-Pico Green (Invitrogen, Carlsbad, CA, United States).

DNA SIP was performed according to the protocol of Neufeld et al. (2007). Equally pooled DNA from the t_0_, ^12^C and ^13^C treatments (5–10 μg) was added to CsCl-containing gradients [buoyant density (BD) 1.732 ± 0.0006 g ml^-1^]. Isopycnic centrifugation was performed (44 100 rpm, i.e., 177 000 g_av_, at 20°C for 40 h; rotor VTi65.2; Beckmann, Fullerton, CA, United States) to separate DNA by its BDs. Gradients were separated into 10 fractions (450 μl each), and the BD of each fraction was determined by repeated weighing at 20°C. DNA was precipitated with glycogen (10 mg ml^-1^) and polyethylene glycol and quantified.

According to the reported BD for non-labeled and fully labeled DNA (Lueders et al., 2004), fractions 1 to 10 were separately pooled into ‘heavy’ (H) fractions (BD ≥ 1.730 g ml^-1^), ‘middle’ (M) fractions (BD between 1.730 and 1.715 g ml^-1^), and ‘light’ (L) fractions (BD ≤ 1.715 g ml^-1^).

### Barcoded Amplicon Pyrosequencing of 16S rRNA Genes, *mxaF/xoxF*, and ITS

DNA from the pooled fractions was used for amplicon pyrosequencing. For bacterial genes (i.e., 16S rRNA and *mxaF/xoxF*), a two-step PCR approach was performed to decrease bias (Berry et al., 2011). In brief, amplicons derived from a first PCR with ‘conventional’ primers (i.e., untagged primers) were subsequently amplified with ‘barcoded’ primers (i.e., primers with an additional barcode sequences at the 5′ terminus) to obtain amplicons with distinguishable nucleotide tags (details in Supplementary Information on Materials and Methods).

Bacterial 16S rRNA gene fragments were amplified using the primers 341f and 785-805r, which had the best overall bacterial sequence and phylum coverage (Muyzer et al., 1998; Herlemann et al., 2011; Klindworth et al., 2013). In order to amplify *mxaF/xoxF* gene sequences the primer pairs ‘mxaF1’ (1003f/1555r; McDonald and Murrell, 1997; Neufeld et al., 2007) and ‘mxaF2’ (mxaF_for/mxaF_rev; Moosvi et al., 2005) were used. Simultaneous amplification of *mxaF* and *xoxF* is assumed since in a previous study *xoxF* gene sequences were also amplified using the primer pair ‘mxaF1’ (Stacheter et al., 2013). Trials to amplify *pmoA* (encodes the beta-subunit of particulate methane monooxygenase) genes were not sufficiently successful, i.e., only a sparse smear or weak bands were visible after PCR and purification led to loss of amplicons. Thus, this gene was no longer analyzed. Barcoded amplicon pools were pyrosequenced at the Göttingen Genomics Laboratory using a Roche GS-FLX 454 Sequencer and GSL FLX Titanium series reagents (Roche Diagnostics GmbH, Mannheim, Germany) as previously described (Stacheter et al., 2013). Fungal ITS fragments (internal transcibed spacer) were amplified using the primers ITS1F and ITS4 (White et al., 1990; Gardes and Bruns, 1993). The amplicons obtained were equimolarly pooled and pyrosequenced at the Department of Soil Ecology (UFZ, Halle, Germany) as previously described (Wubet et al., 2012). For detailed information on the primer sequences, PCR conditions, strategies and performance, please refer to the Supplementary Information on Materials and Methods.

### Read Filtering and Clustering

The reads of bacterial genes were trimmed to nearly equal sequence lengths (446 bp for 16S rRNA, 440 bp for *mxaF*), amplicon pyrosequencing errors were corrected using ACACIA, and potential 16S rRNA chimeric sequences were sorted out using the UCHIME algorithm implemented in USEARCH and the latest RDP Gold database for high-quality 16S rRNA gene reference sequences (Edgar et al., 2011; Bragg et al., 2012). Using JAguc v2.1, the sequences were clustered into operational taxonomic units (OTUs) applying the UPGMA model (Nebel et al., 2011). The OTUs of 16S rRNA were clustered at the family level using 90.1% as the pairwise similarity cut-off value (to ensure sufficient sampling depth), and *mxaF* OTUs were clustered with a cut-off value of 90%, which was higher than that previously reported, to obtain a higher diversity (Yarza et al., 2010; Stacheter et al., 2013). Representative sequences (i.e., longest sequence, in the case of identical lengths the sequence was randomly chosen by the program) of each OTU were used for further taxonomic affiliation. 16S rRNA phylotypes were primarily affiliated using a local nucleotide BLAST and affiliation was checked by a online megaBLAST that uses a nucelotide database updated daily and a phylogenetic tree generated using MEGA Version 6.06 (Tamura et al., 2013). The phylogenetic affiliation of *mxaF* phylotypes was determined using manual BLAST (megaBLAST) and phylogenetic treeing.

For the detailed resolution of OTU_16S_438 the filtered 16S rRNA gene dataset (also used in the JAguc analysis) and all sequences of OTU_16S_438 were combined and clustered using QIIME at a species-level cut-off. For more detailed information refer to the Supplementary Information on Materials and Methods.

Reads of fungal ITS genes were demultiplexed and quality trimmed using MOTHUR, normalized (1503 counts per sample), and checked for chimeric sequences using UCHIME (Schloss et al., 2009; Edgar et al., 2011). The sequences were clustered into OTUs using CD-HIT-EST at a 97% pairwise similarity cut-off value (Fu et al., 2012). Representative sequences were classified against the dynamic UNITE database (v7 release 01.08.2015; Kõljalg et al., 2013) using a MOTHUR-implemented classifier of Wang et al. (2007). For detailed information, please refer to the Supplementary Information on Materials and Methods.

### Identification of ‘^13^C-Labeled’ Phylotypes

The ‘^13^C-label’ of phylotypes was determined by analyzing the relative abundances of phylotypes in the amplicon read libraries of the H, M, and L fractions of the [^12^C] and [^13^C] treatments of each gene dataset (i.e., 16S rRNA gene, *mxaF*, and ITS). The phylotypes that occurred only once within the complete dataset of all amplicon libraries were considered erroneous and removed, whereas singletons in each individual amplicon library were preserved (not applied for the ITS dataset, in which phylotypes with fewer than three reads were removed in the previous read-filtering step). A comparison of the relative abundances in the H fractions of [^13^C] treatments with those in the H fractions of [^12^C] treatments and a comparison of the H and L fractions of the [^13^C] treatment were conducted. This procedure minimizes the identification of false-positive phylotypes due to the migration of light DNA into the H fractions (Lueders et al., 2004; Dallinger and Horn, 2014). The following criteria had to be met to classify a phylotype as ‘labeled’: (1) The abundance in the appropriate fraction (i.e., H or M fraction) of the [^13^C] treatment was higher than that in the corresponding fraction of the [^12^C] treatment; (2) the abundance in the L fraction was lower than that in the H or M fraction of the [^13^C] treatment; (3) the abundance in the H or M fraction of the [^13^C] treatment was ≥0.5%; and (4) the difference in the abundance in the compared fractions of the [^13^C] treatment was ≥0.1% compared to that of the [^12^C] treatment. Phylotypes that met all these criteria were considered ‘potentially labeled’ and were the basis for the calculation of the ‘labeling proportion’ (LP). The LP serves as an indicator for the relative importance of different bacterial taxa assimilating the supplemented [^13^C_u_]-substrate (directly or indirectly) and is not a proxy for the amount of incorporated ^13^C into the DNA. The LP of a certain potentially labeled phylotype x was

$$\begin{array}{c} {\mathrm{LP}}_{x}=\frac{100}{\sum_{i=1}^{n}{\mathrm{RA}}_{i}^{13C}}\times {\mathrm{RA}}_{x}^{13C} \end{array}$$

with RA is the relative abundance, n is the number of all ‘potentially labeled’ phylotypes, $\sum_{i=1}^{n}{\mathrm{RA}}_{i}^{13C}$ is the sum of all relative abundances of ‘potentially labeled’ taxa in the M or H gradient fraction of the [^13^C] treatment, and ${\mathrm{RA}}_{x}^{13C}$ is the relative abundance of a certain phylotype x in the M or H gradient fraction of the [^13^C] treatment. A threshold value of 5% was used to distinguish between labeled taxa of greater (i.e., LP_x_ ≥ 5%) or minor (i.e., LP_x_ < 5%) importance (Dallinger and Horn, 2014). The phylotypes that were identified as labeled in the M fraction were considered as ‘weakly labeled’ for two possible reasons: (i) not fully labeled DNA and (ii) fully labeled DNA of organisms with very low GC content (<40%). Nonetheless, we expected that the general genome GC content is higher than 40% and is similar for the majority of microorganisms of this environment as previous genome studies suggest (Foerstner et al., 2005).

### Quantification of 16S rRNA Genes, *mxaF*, and *mmoX* in the pH Shift SIP Experiment

The gene fragments were quantified in duplicates on an iQ5 Real-Time qPCR cycler (BioRad, Munich, Germany) with primer sets specific for *Bacteria* and *mxaF* (Moosvi et al., 2005) and *mmoX* (Kolb et al., 2005) using internal standards. According to published protocols, all qPCR measurements were inhibitor corrected because co-extracted humic acids were obvious and inhibition was well-recorded (Degelmann et al., 2010; Zaprasis et al., 2010) (detailed information in Supplementary Information on Materials and Methods).

### Nucleotide Sequence Accession Numbers

Representative sequences of labeled phylotypes derived from barcoded amplicon pyrosequencing were deposited in EMBL under accession numbers LT607885 to LT607955 (16S rRNA gene), LT607956 to LT608017 (*mxaF*), and LT608018 to LT608119 (ITS). All raw pyrosequencing datasets were deposited in the ENA Short Read Archive under the study accession number ERP016444, including the 16S rRNA gene, *mxaF* and ITS datasets.

## Results

### Identification of Microorganisms Utilizing Methanol-Derived Carbon in an Acidic Forest Soil

A successful labeling of *Bacteria* and fungi was proven by the dissimilar composition of phylotypes of [^12^C] and [^13^C] H and M fractions (Supplementary Figure S1). In addition, the carbon recovery rates for [^13^C]-CO_2_ revealed that the supplemented substrates were not only dissimilated but were also assimilated (Supplementary Figures S3, S4).

**FIGURE 1:**
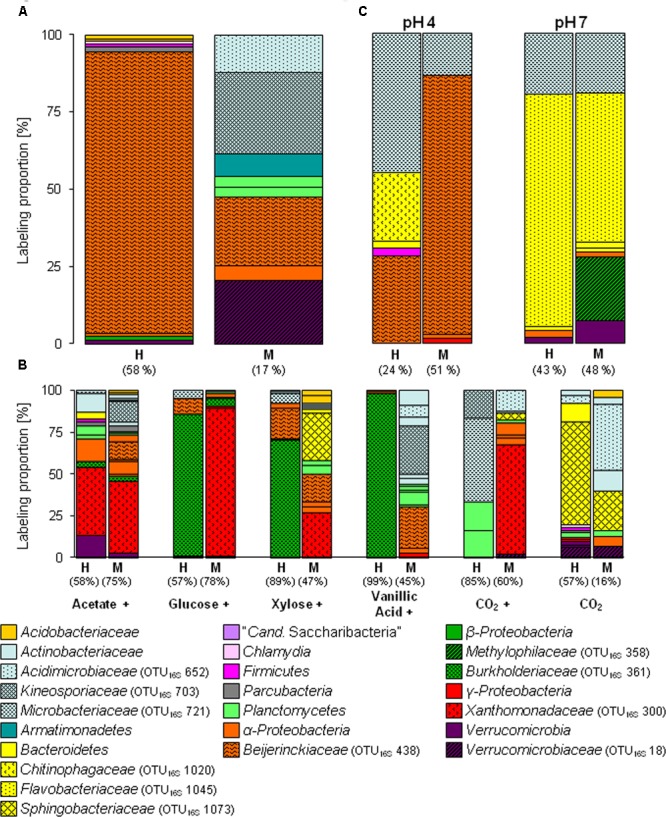
Labeling proportions (LPs) and identities of bacterial 16S rRNA phylotypes in the ‘heavy’ and ‘middle’ fractions of [^13^C_1_]-methanol **(A)** and [^13^C_u_]-substrates **(B)** treatments of the substrate SIP experiment, and treatments of the pH SIP experiment **(C)**. Cross, additional [^12^C]-methanol supplementation in substrate treatments. Equal colors, the same phylum affiliation. ‘H’ and ’M,’ ‘heavy’ and ‘middle’ fractions, respectively. Values in brackets, contribution of labeled phylotypes to the total number of sequences. The ‘labeling proportions’ are indicators for the relative importance of different taxa assimilating the supplemented ^13^C (directly or indirectly). More detailed taxonomic information can be found in Supplementary Tables S16, S17.

The [^13^C_1_]-methanol treatment of the substrate SIP experiment revealed one labeled phylotype (OTU_16S_438) dominating the H fraction. This phylotype was also highly labeled in the M fraction and was affiliated to *Beijerinckiaceae*, i.e., it was closely related to methylotrophic species such as *Methylovirgula ligni* (**Figure 1A** and Supplementary Tables S1, S2). Further minor and weakly labeled phylotypes were found among a wide range of bacterial phyla (i.e., minor labeled, LP < 2%: *Acidobacteria, Proteobacteria*, and *Verrucomicrobia;* weakly labeled: *Armatimonadetes, Planctomycetes, Alphaproteobacteria*, and *Verrucomicrobia*) (**Figure 1A** and Supplementary Table S1). The sequence identities of these labeled phylotypes to their closest related cultured species ranged from 83 to 99% (Supplementary Table S2), suggesting that hitherto unrecognized methanol-utilizers or methanol-derived carbon-utilizers occurred.

The [^13^C_1_]-methanol treatment of the pH shift SIP experiment revealed a slightly different distribution of labeled phylotypes compared to the substrate SIP experiment suggesting an influence of methane on the methanol-utilizer community in the forest soil. In addition to the *Beijerinckiaceae*-phylotype (OTU_16S_438), also phylotypes affiliated to *Microbacteriaceae* (OTU_16S_721, 99% sequence identity to *Leifsonia xyli*) and *Chitinophagaceae* (OTU_16S_1020, 96% sequence identity to *Chitinophaga* sp.) were labeled (**Figure 1C** and Supplementary Table S6). Further minor or weakly labeled phylotypes belonged to *Sphingobacteriales, Paenibacillaceae, Sphingomonadaceae*, and *Xanthomonadaceae* (*Rhodanobacter*) (**Figure 1C** and Supplementary Table S6).

### Labeled Phylotypes Based on MDH Gene Markers

The specific primers used in our study were assumed to target *mxaF* as well as *xoxF* gene sequences as a previous study on temperate soils indicated (Stacheter et al., 2013). However, no *xoxF*-affiliated phylotype was labeled in any treatment (including also [^13^C_u_]-substrate treatments of the substrate SIP experiment), and potentially further present *xoxF*-containing methylotrophs might have been overlooked. The labeled phylotypes of the [^13^C_1_]-methanol treatment of the substrate SIP experiment were dominated by three phylotypes belonging to *Methylobacterium* (OTU_mxaF_40) and *Hyphomicrobium* (OTUs_mxaF_185 and 210), whereas in the [^13^C_1_]-methanol treatment of the pH shift SIP experiment labeled *mxaF* phyloytpes belonged mainly to *Hyphomicrobium*.

**FIGURE 2:**
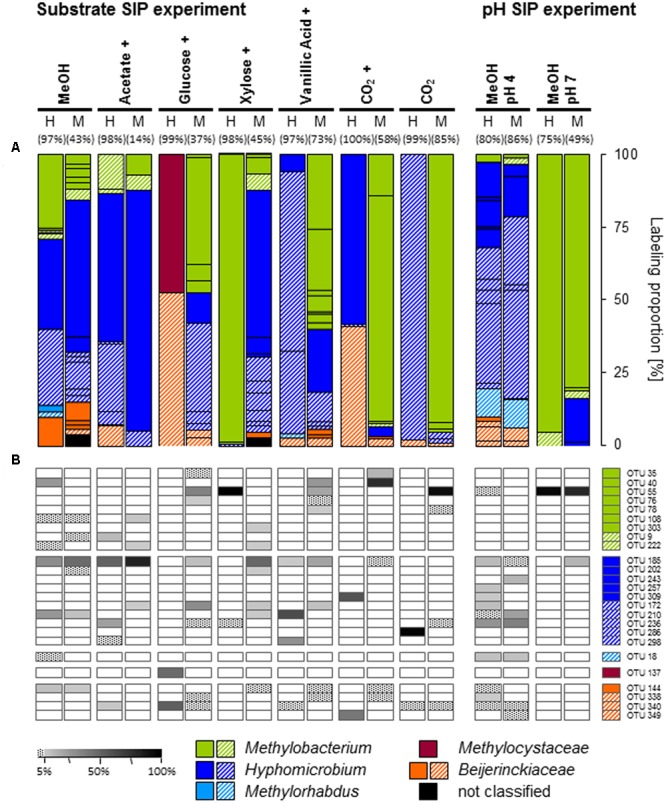
Labeling proportions and identities of *mxaF* phylotypes in the ‘heavy’ and ‘middle’ fractions of all labeled *mxaF* phylotypes in substrate or pH treatments **(A)** and the relative frequencies of labeled phylotypes (i.e., gray shades, ‘labeling proportion’ >5%; dotted, ‘labeling proportion’ <5%) **(B)**. Cross, additional [^12^C]-methanol supplementation in the substrate SIP experiment. Equal colors, the same family affiliation. Shading, ambiguous affiliation (i.e., sequence identity with BLASTn <90% and an ambiguous position in the phylogenetic tree). ‘H’ and ’M,’ ‘heavy’ and ‘middle’ fractions, respectively. Values in brackets, contribution of labeled OTUs to the total number of sequences. The ‘labeling proportions’ are indicators for the relative importance of different taxa assimilating the supplemented ^13^C (directly or indirectly). More detailed taxonomic information can be found in Supplementary Table S15.

Although *Beijerinckiaceae* appeared to be the dominantly labeled bacterial phylotype based on the 16S rRNA genes, only one *Beijerinckiaceae*-related *mxaF* phylotype (OTU_mxaF_144) was labeled in the substrate SIP experiment (Supplementary Table S3 and **Figure 2A**). Methane might have stimulated this *mxaF*-phylotype since its LP was lower in the methane-free treatment of the pH shift SIP experiment. In this treatment another *Beijerinckiaceae*-phylotype (OTU_mxaF_338) and a *Methylorhabdus*-affiliated phylotype (OTU_mxaF_18) were weakly labeled (**Figure 2A** and Supplementary Table S8, Figures S5–S7, S9).

### Fungi Assimilating Methanol-Derived Carbon

**FIGURE 3:**
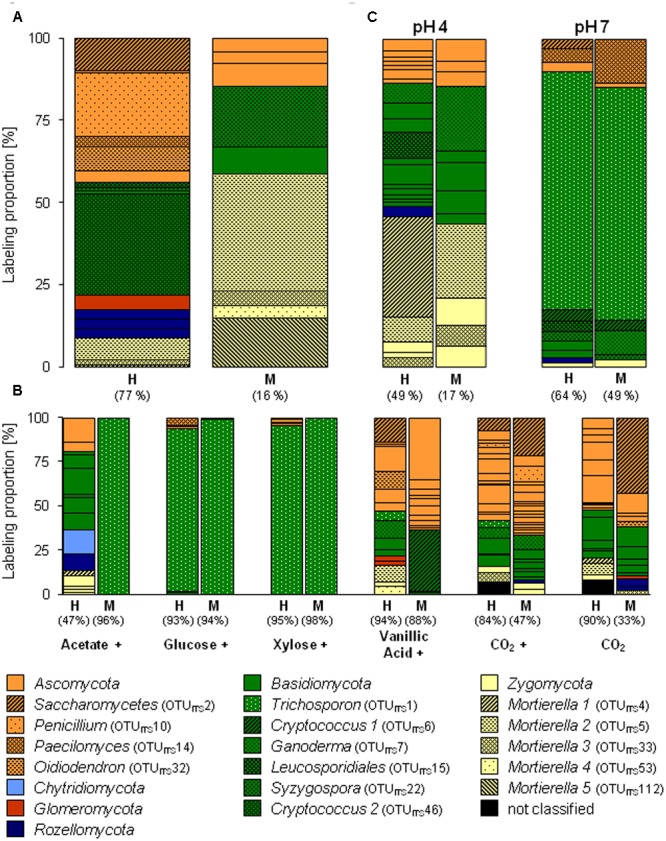
Labeling proportions and identities of fungal ITS phylotypes in the ‘heavy’ and ‘middle’ fractions of [^13^C_1_]-methanol **(A)** and different [^13^C_u_]-substrates **(B)** treatments of the substrate SIP experiment, and of the treatments of the pH SIP experiment **(C)**. Cross, additional [^12^C]-methanol supplementation in substrate treatments. Equal colors, the same phylum affiliation. ‘H’ and ‘M,’ ‘heavy’ and ‘middle’ fractions, respectively. Values in brackets, contribution of labeled OTUs to the total number of sequences. The ‘labeling proportions’ are indicators for the relative importance of different taxa assimilating the supplemented ^13^C (directly or indirectly).

Apart from *Bacteria* also fungi assimilated methanol-derived carbon (**Figure 3A** and Supplementary Table S4). In the [^13^C_1_]-methanol treatment of the substrate SIP experiment, one abundantly labeled phylotype was affiliated to the basidiomycetous yeast *Cryptococcus* (OTU_ITS_46). Further labeled phylotypes (LP ≥ 5%) were affiliated to *Ascomycota* (OTUs_ITS_10, 32, and 2) and *Zygomycota* (OTU_ITS_5; *Mortierella* sp., 98% sequence identity) (**Figure 3A** and Supplementary Tables S4, S5). A weak label was observed for phylotypes affiliated to *Zygomycota* (OTU_ITS_112), *Basidiomycota* (OTUs_ITS_22 and 27), and *Ascomycota* (OTU_ITS_135) (**Figure 3A** and Supplementary Tables S4, S5). Consistently with the [^13^C_1_]-methanol treatment of the substrate SIP experiment, the zygomycetous phylotypes of the [^13^C_1_]-methanol treatment of the pH shift SIP experiment were affiliated to the genus *Mortierella* (**Figure 3C** and Supplementary Tables S5, S10). In contrast, further labeled fungal phylotypes were affiliated to *Basidiomycota* (OTUs_ITS_30, 15, 12, and 7) (**Figure 3C** and Supplementary Table S10), and a remarkable number of weakly labeled phylotypes were affiliated to *Ascomycota, Basidiomycota*, and *Zygomycota* (**Figure 3C** and Supplementary Table S10).

### Effect of pH on the Abundance of *Bacteria* and Methylotrophs

**FIGURE 4:**
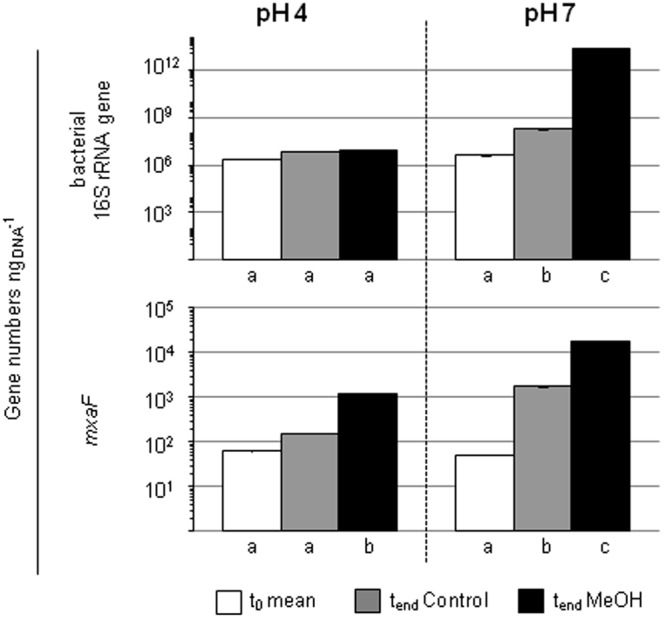
Gene numbers of 16S rRNA genes and *mxaF* of treatments with different pH in the pH shift SIP experiment. Columns, mean values of the experimental replicates. Error bars, standard deviation; if not visible, the variability between replicates was below 0.5%. Different letters, significant differences between samples (*t*-test; normal distribution was assumed based on the Shapiro-Wilk-test; *n* = 3).

The quantification of the bacterial abundance (16S rRNA gene numbers) and *mxaF* gene numbers revealed an increase in both pH 7 treatments (i.e., the unsupplemented controls and methanol treatments), demonstrating the general growth restrictions at the *in situ* pH 4 for *Bacteria* and methylotrophs (**Figure 4**). However, a decrease of *mmoX* gene numbers in the pH 7 treatment occurred, whereas the values remained constant in the pH 4 treatments (Supplementary Figure S2) suggesting that the acidic *in situ* pH conditions were advantageous for methane-utilizing *Beijerinckiaceae.* Since the amplicons were not checked for their identity, a detection of non-target sequences cannot be excluded and thus this result should be regarded with caution.

### Identification of Autochthonous Microorganisms that Utilized Methanol-Derived Carbon under Elevated pH Conditions

As expected several different phylotypes were labeled under the elevated pH condition indicating again the growth restricting conditions of the *in situ* acidic soil. In accordance with a generally increasing proportion of *Bacteroidetes* (Supplementary Figure S3A) in the pH 7 treatment, the most abundantly labeled phylotypes affiliated with *Flavobacteriaceae* (OTU_16S_1045, 99% sequence identity to *Chryseobacterium* sp.) and *Microbacteriaceae* (OTU_16S_721, 99% sequence identity to *Leifsonia xyli*) (**Figure 1C** and Supplementary Tables S2, S7). Weakly labeled phylotypes (i.e., LP < 3%) were affiliated to *Sphingobacteriaceae, Caulobacteraceae*, and *Verrucomicrobia* with partially low sequence identities to the next related sequence of a cultured isolate (Supplementary Table S2). A *Methylophilaceae*-related phylotype (OTU_16S_358) was only weakly labeled but with a high LP of approximately 20% (**Figure 1C** and Supplementary Tables S2, S7). This phylotype was not labeled in the pH 4 treatment (**Figures 1A,C**), which suggests a decreased competitiveness and initial low abundance under *in situ* conditions. Further weakly labeled phylotypes were affiliated with *Bacteroidetes, Alphaproteobacteria*, and *Verrucomicrobia* (**Figure 1C** and Supplementary Tables S2, S7, Figure S10).

In contrast to the aforementioned findings on labeled *mxaF*-phylotypes, a *Methylobacterium*-related phylotype (OTU_mxaF_55) was highly abundant indicating the preference of neutral pH conditions (**Figure 2A** and Supplementary Table S9).

A preference of a neutral pH was also observed for one fungal phylotype affiliated to the yeast *Trichosporon* (OTU_ITS_1), that was abundantly labeled under an elevated pH. Further weakly labeled fungal phylotypes were affiliated to *Ascomycota* and *Basidiomycota* (**Figure 3C** and Supplementary Table S11), which suggests that these taxa did not play a dominant role in methanol assimilation.

### The Substrate Range of Methanol-Derived Carbon-Utilizing Microorganisms

As expected, several taxa not known to include methylotrophs such as *Xanthomonadaceae* (OTU_16S_300, *Rhodanobacter*), *Burkholderiaceae* (OTU_16S_361, *Burkholderia*), and *Sphingobacteriaceae* (OTU_16S_1073, *Mucilaginibacter*) were labeled in the multi-carbon substrate treatments. If these microorganisms dissimilated methanol without assimilating carbon remains speculative. ‘True’ methanol and/or methanol-derived carbon utilizers (i.e., assimilation of carbon from methanol) were identified in the [^13^C_1_]-methanol treatment. The congruent detection of a phylotype in both, the methanol and an alternative substrate treatment, was considered to estimate the putative substrate range of methanol-derived carbon utilizers.

**FIGURE 5:**
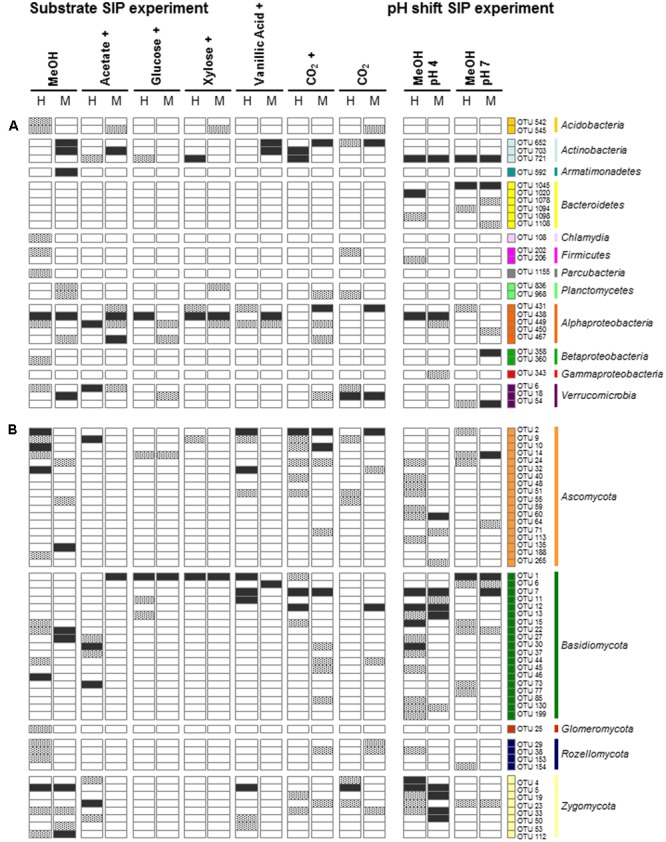
Bacterial **(A)** and fungal **(B)** phylotypes labeled in the methanol treatment of the substrate SIP experiment and their congruent labeling in other treatments of both SIP experiments. Black, LP > 5%; gray, LP < 5%; white, not labeled. The phylotypes that were only labeled in treatments with multi-carbon substrates or in the pH shift SIP experiment are not presented. Cross, additional [^12^C]-methanol supplementation in substrate treatments. Equal colors, the same phylum affiliation. ‘H’ and ‘M,’ ‘heavy’ and ‘middle’ fractions, respectively.

**FIGURE 6:**
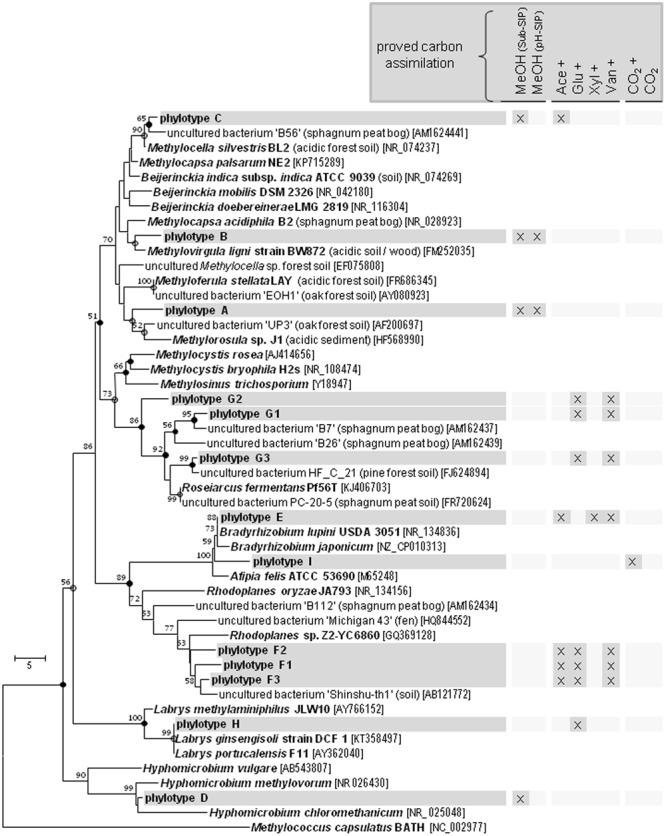
Species-Level resolution of the *Beijerinckiaceae*-affiliated phylotype OTU_16S_438. Species-level phylotypes of the *Beijerinckiaceae*-affiliated OTU_16S_438 (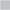, phylotype A to I based on species level similarity cut-off) and their putative substrate utilization. Outgroup, 16S rRNA sequence of *Methylococcus capsulatus* BATH. Bootstrap were based on 1000 replicated calculations. The trees was calculated with the neighborjoining method. Dots at the nodes, congruent nodes with trees caculated with the maximum likelihood and maximum parsimony method (●, true for three methods; 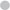, true for two methods). If known the isolation origin of each sequence is given in brackets. Accession numbers are given in squared brackets. The bar indicates 5 changes per nucleotide. ‘Sub-SIP,’ substrate SIP experiment; ‘pH-SIP,’ pH shift SIP experiment; cross, additional [^12^C]-methanol supplementation in substrate treatments of the substrate SIP experiment.

The *Beijerinckiaceae-*phylotype (OTU_16S_438) was labeled in all treatments with multi-carbon compounds, which suggests that this phylotype assimilates carbon derived from acetate, sugars and even aromatic compounds in the presence of methanol (**Figure 5**). A more detailed analysis on species-level revealed that several phylotypes were grouped together by OTU_16S_438 and that clearly different trophic types belonged to this OTU (**Figure 6**). Species-level phylotypes that were identified as obligately methylotrophic (A, B, and D) were closely affiliated with known methylotrophs of *Beijerinckiaceae* (i.e., *Methylorosula, Methyloferula*, and *Methylovirgula*) and *Hyphomicrobiaceae* (i.e., *Hyphomicrobium*). Phylotype C was identified as restricted facultatively methylotrophic and was closely affiliated with *Methylocella*. Further phylotypes (E, F, G, H, and I) were affiliated with several members of *Rhizobiales* but were apparently non-methylotrophic. Low LPs and sometimes only a weak labeling of the *Beijerinckiaceae*-affiliated phylotype (family-level) suggested a higher competition or slower growth rates with multi-carbon substrates. A potentially occuring cross-feeding via the assimilation of ^13^CO_2_ can be considered as negligible because OTU_16S_438 was not labeled in the ^13^CO_2_ treatments (**Figures 1B, 5**). However, the utilization of methanol cannot fully be excluded for the apparent non-methylotrophic phylotypes since SIP analysis cannot resolve the sole dissimilation of methanol or the indirect carbon assimilation via CO_2_.

Further alphaproteobacterial methanol-derived carbon-assimilating phylotypes exhibited both a broad substrate range (OTU_16S_449; *Sphingomonadaceae*) and a narrow substrate range (OTU_16S_467; *Acetobacteraceae*) (**Figure 5**). Interestingly, most of the 16S rRNA phylotypes that were only labeled in the pH 7 treatment were not labeled at the acidic *in situ* pH (**Figure 5**) suggesting an inhibition or even growth-restriction and thus no utilization of any multi-carbon substrate. Only the *Microbacteriaceae*-phylotype (OTU_16S_721, *Leifsonia* spp.) exhibited a broad substrate and pH range (**Figure 5**).

All *mxaF* phylotypes that were labeled in the methanol treatment of the substrate SIP experiment were also labeled in the multi-carbon substrate treatments (**Figure 2B** and Supplementary Table S3). A *Hyphomicrobium*-phylotype (OTU_mxaF_185) exhibited the broadest substrate range and was detectable in all treatments supplemented with methanol (^12^C and ^13^C). A labeling in both the acetate and vanillic acid treatments was observed with high LPs for acetate indicating a preference for this substrate (**Figure 2B**). The weak labeling in both sugar treatments suggested a low carbon assimilation rate and/or a general higher competitive pressure through non-methylotrophic heterotrophs (**Figure 2B**). OTU_mxaF_185 was also labeled in the pH shift SIP experiment, but a weaker labeling at pH 7 indicated a preference for acidic conditions (**Figure 2B** and Supplementary Tables S8, S9). The weak labeling of a *Beijerinckiaceae*-phylotype (OTU_mxaF_144) in treatments containing xylose, vanillic acid, or CO_2_ plus methanol (**Figure 2B**) suggested facultative methylotrophy with a preference for methanol and/or a slower growth rate on multi-carbon substrates. Apart from the already mentioned methylotrophic families a *Methylocystaceae*-affiliated phylotype (OTU_mxaF_137) was labeled in one multi-carbon substrate treatment (i.e., the treatments with [^13^C_6_]-glucose, **Figure 2A**). This phylotype might be only little competitve under methylotrophic conditions. Only a few *mxaF* phylotypes of *Methylobacterium* and *Hyphomicrobium* were solely labeled in multi-carbon substrate treatments. This observation suggests a preference for multi-carbon substrates over methanol by these phylotypes or a weak competiveness for methanol (**Figure 2B**). Under experimentally elevated pH another *Methylobacterium*-phylotype (OTU_mxaF_55) was abundantly labeled. Its facultatively methylotrophic lifestyle can be concluded from the fact that it was labeled in both sugar and vanillic acid treatments (**Figure 2B** and Supplementary Table S9). Obviously, OTU_mxaF_55 had a preference for more neutral pH conditions, because it was only weakly labeled in the pH 4 treatment (**Figure 2B** and Supplementary Tables S8, S9).

The fungus *Cryptococcus* (OTU_ITS_46; *Basidiomycota*) was abundant in the H fraction of labeled fungal phylotypes in the [^13^C_1_]-methanol treatment of the substrate SIP experiment (**Figures 3, 5**). Although a general increase in the relative abundance of *Cryptococcus*-related phylotypes was observed in the vanillic acid treatment (Supplementary Table S12), another *Cryptococcus* phylotype (OTU_ITS_6) was weakly labeled in the vanillic acid treatment (**Figure 5**). An assimilation of the methyl group of aromatic compounds is conceivable, as well as the possibility of utilizing carbon derived from the breakdown products of vanillic acid.

Three ascomycetous phylotypes (OTU_ITS_2, *Saccharomycetes;* OTU_ITS_32, *Oidiodendron;* and OTU_ITS_5, *Mortierella*) were labeled in the methanol and vanillic acid treatments and *Mortierella*-related phylotypes were detectable with low LPs in both methanol treatments at the *in situ* pH and the vanillic acid treatment (**Figures 3, 5**). The broadest substrate range was revealed by a *Trichosporon*-phylotype (OTU_ITS_1) labeled in acetate, sugar and vanillic acid treatments. Moreover, methanol utilization cannot be excluded for this phylotype because it was also labeled in the pH 7 treatment of the pH shift SIP experiment, which suggests an assimilation of methanol or at least the utilization of methanol-derived carbon (**Figures 3, 5**).

## Discussion

Stable isotope probing combined with high-throughput sequencing have expanded our knowledge on the phylogenetic diversity and global distribution of methylotrophs in terrestrial ecosystems (e.g., Kolb and Stacheter, 2013; Stacheter et al., 2013; Chistoserdova, 2015). However, their significant role in ecosystem-level methanol cycling has been largely neglected, although terrestrial ecosystems are strong sources and sinks. Thus, methylotrophs have a direct impact on global methanol fluxes and consequently on the global atmospheric chemistry (Galbally and Kirstine, 2002; Kolb, 2009; Stacheter et al., 2013). Furthermore, the role of methylotrophic yeasts and fungi, has hardly been investigated in soils as well as the impact of methanol-derived carbon on the soil microbial food web (Lueders et al., 2004; Kolb, 2009).

### The Dominant Methanol-Utilizing *Bacteria* Possess a Restricted Substrate Range

The main bacterial methanol-utilizers were affiliated with *Beijerinckiaceae*. This finding is consistent with studies on other acidic soils and species descriptions of acidophilic methylotrophic *Beijerinckiaceae* (Radajewski et al., 2000, 2002; Dedysh et al., 2001, 2006; Morris et al., 2002; Marín and Arahal, 2013 and references therein). The genera *Chelatococcus* and *Camelimonas* were excluded from all considerations on *Beijerinckiaceae*, since they are not capable of C_1_ compound utilization (Dedysh et al., 2016). *Beijerinckiaceae* comprises strains with remarkably different metabolic capacities including chemoheterotrophy (*Beijerinckia*), facultative methylotrophy (*Beijerinckia, Methylorosula, Methylovirgula*), ‘restricted’ facultative methanotrophy (*Methylocella, Methylocapsa*), and obligate methanotrophy (*Methylocapsa, Methyloferula*) (Marín and Arahal, 2013 and references therein; Tamas et al., 2014; Dedysh et al., 2016).

The metabolic behavior of facultative methylotrophs under *in situ* conditions has not been resolved, since multi-carbon utilization studies are often conducted as a comparison between methylotrophic (only C_1_ compounds being supplemented) and multi-carbotrophic (only multi-carbon compounds being supplemented) conditions. Such studies on pure cultures of *Methylobacterium extorquens* AM1 revealed a high methanol oxidation capacity in the presence of alternative multi-carbon substrates (i.e., multi-carbotrophic followed by methylotrophic conditions) or mixed substrate conditions suggesting that methanol is primarily used for energy conservation (Bosch et al., 2008; Skovran et al., 2010; Peyraud et al., 2011, 2012). Regrettably, such carbon flux studies are not available for members of the *Beijerinckiaceae* but we assume a similar regulation phenomenon in our experiment. We detected a *Beijerinckiaceae*-affiliated phylotype (OTU_16S_438), that comprises different trophic types on species-level resolution. However, due to the limited phylogenetic resolution of the analyzed 16S rRNA gene fragment (444 bp) and the close relation of *Rhizobiales* members, an unequivocal determination of the genus or species would still be rather vague. The species-level phylotypes A, B, C, and D were closely affiliated with known methylotrophs. The phylotypes A, B, and D were apparently limited in their substrate range to methanol only. The phylotype C revealed a restricted substrate range including methanol and acetate and was closely related with *Methylocella*, which was the first genus reported as facultatively methanotrophic (Dedysh et al., 2005a). Among the facultatively methylotrophic *Beijerinckiaceae* the documented multi-carbon substrate range is also usually limited to only a few carboxylic acids (Dedysh et al., 2005a; Vorobev et al., 2009; Marín and Arahal, 2013). There exist only two exceptions – (i) *Beijerinckia mobilis*, that is to date the only known methanol-utilizer of the metabolically versatile genus *Beijerinckia*, and (ii) *Methylorosula polaris*, which exhibits a broad substrate range including sugars and polysaccharides (Dedysh et al., 2005b; Berestovskaya et al., 2012; Marín and Arahal, 2013). However, in our substrate SIP experiment none of these facultative methylotrophs assimilated carbon from multi-carbon substrates.

Apart from a direct substrate utilization several methylotrophic *Beijerinckiaceae* assimilate carbon at the level of CO_2_ such as *Methyloferula, Methylorosula*, and *Methylovirgula* (Marín and Arahal, 2013 and references therein; Vorobev et al., 2011). Thus, the oxidation of endogenous or supplemented substrates to CO_2_ by chemoheterotrophic microorganisms might have supported methylotrophic *Beijerinckiaceae* and could have led to a dilution of a conceivable ^13^C-signal, although we tried to minimize such an effect by several experimental measures.

A potentional to utilize methane was somewhat likely for some of the labeled *Beijerinckiaceae*-associated phylotypes since *mmoX* genes (encoding for *Beijerinckiaceae*-associated sMMO) were detectable by qPCR (Dedysh et al., 2005a; Vorobev et al., 2011; Marín and Arahal, 2013). However, a final proof of their identities by sequencing of the amplicon is missing. Additionally, a growth stimulating effect of methane was also observed when comparing both methanol treatments of both SIP experiments (i.e., the substrate SIP and the pH shift SIP experiment), which revealed a higher LP when methane was available. Taken together, our findings suggest that (i) *Beijerinckiaceae*-affiliated taxa were important methanol-utilizers in the forest soil, (ii) that their substrate range might be strongly limited to methanol under *in situ* conditions, and (iii) that the capability of methanol utilization defines their ecological niche in a complex forest soil microbiome.

All apparently non-methylotrophic species-level members of the OTU_16S_438 (phylotypes E to I) affiliated with different members of *Rhizobiales.* For these phylotypes an assimilation of carbon derived from methanol could not be confirmed.

The phylotypes E and I were affiliated to *Bradyrhizobiaceae* that are trophically versatile. Methylotrophy cannot be fully excluded since *Bradyrhizobium* species posses *xoxF* genes and MDH activity but exhibit only weak growth (Kaneko et al., 2002; Sudtachat et al., 2009; Fitriyanto et al., 2011; de Souza et al., 2013). Thus, the low concentrations of methanol (i.e., 1 mM per pulse) led likely to a low ^13^C incorporation and might have prevented the verification of methanol utilization. The substrate range of the phylotype E included acetate, xylose, and vanillic acid. Utilization of methylated aromatic compounds causes an upregulated expression of C_1_ metabolism genes such as *xoxF* (Ito et al., 2006). The observed ability of *Bradyrhizobium* species to fix CO_2_ is in accordance with the detection of phylotype I in the CO_2_ treatments (Masuda et al., 2010). Thus, CO_2_ fixation might have caused a dilution of the ^13^C signal and thus prevented the proof of methanol utilization.

Phylotype H was affiliated to *Labriaceae* (*Labrys*). Isolates of this family possess *xoxF-like* genes (Beck et al., 2015). The only methylotrophic species reported to date is *Labrys methylaminiphilus* that utilizes methylamine and several monosaccharides, but not methanol (Miller et al., 2005). However, also *L. monachus* can utilize methanol to a certain extent (Miller et al., 2005). Thus, the genus *Labrys* might comprise hitherto unknown methanol utilizers.

The phylotypes F and G were closely affiliated to environmental sequences. Classification revealed an affiliation with *Rhodoplanes* (*Hyphomicrobiaceae*). Nonetheless, several reads of phylotype F were closely related with *Methylocystaceae* (*Methylosinus*) and *Xanthobacteraceae* (*Variibacter, Pseudolabrys*) rendering an unambiguous affiliation hardly possible. Interestingly, studies in peat bogs addressing methanotrophs identified also sequences somehow affiliated to *Rhodoplanes* by using methanotrophic specific probes (i.e., *Methylosinus* specific) but were also not able to verify methylotrophy of these taxa (McDonald et al., 1996, 1999). Thus, this cluster of *Rhodoplanes*-affiliated sequences remains enigmatic regarding methylotrophy. Another still enigmatic taxon is phylotype G that was affiliated with several environmental sequences that were in turn affiliated with *Methylocystaceae* and *Beijerinckiaceae*. Within this cluster, only one species (*Roseiarcus fermentans*) has been taxonomically described. However, this species cannot utilize C_1_ compounds (Kulichevskaya et al., 2014).

### Discrepancy of Identified Methanol-Utilizers Based on 16S rRNA Gene and *mxaF*

Most *mxaF* phylotypes were affiliated with *Methylobacteriaceae* and *Hyphomicrobiaceae* instead of *Beijerinckiaceae*, and no significant similarity to *mxaF* from a previous study on a forest soil or *mxaF* genes of *Methylovirgula ligni* was evident (Radajewski et al., 2002; Vorobev et al., 2009). *Beijerinckiaceae* can harbor *mxaF* sequences, which are similar to that of *Methylobacterium* or *Hyphomicrobium* due to horizontal gene transfer (HGT) events that occurred during the evolution of this family (Lau et al., 2013; Tamas et al., 2014).

Interestingly, one *mxaF* phylotype affiliated with *Methylocystaceae* (OTU_mxaF_137) was labeled under mixed substrate conditions with glucose although *Methylocystaceae* were not abundant in the bacterial community based on both gene markers (16S rRNA and *mxaF*, Supplementary Figures S2, S8). *Methylocystaceae* comprise restricted facultative methanotrophs, i.e., strains of the *Methylocystis* slowly grow on acetate or ethanol (Belova et al., 2011). However, the utilization of glucose has never been reported. Thus, it is possible that OTU_mxaF_137 is a hitherto unknown glucose-utilizing member of *Methylocystaceae* or the specific *mxaF* genotype has been horizontally transferred to the methanotrophic sister genera.

Another aspect is the lack of detection of *xoxF* genes. Both genes possess different functions and phylogenetic distributions. At the time conducting our experiments an unequivocal grouping between *mxaF* genes and five distinct clades of *xoxF* genes (*xoxF1* to *xoxF5*) was known (Chistoserdova, 2011; Keltjens et al., 2014). Only recently, *xoxF* gene sequences were analyzed in detail, which enabled the development of divergent *xoxF* primers for the different clades (Taubert et al., 2015). Thus, targeting both *mxaF* and *xoxF* genes with only one primer pair might result in biased amplification results toward *mxaF*.

### Putative Fungal Methanol-Utilizers

Until now, only a limited number of methylotrophic fungi belonging to yeasts or molds (mainly *Ascomycota*), have been reported (Kolb, 2009; Kolb and Stacheter, 2013). In our study, fungal phylotypes that assimilated methanol-derived carbon were basidiomycetous yeasts (*Tremellomycetes*), in particular *Cryptococcus* and *Trichosporon*, and the zygomycetous genus *Mortierella*. These genera are globally abundant in soil and are saprotrophs that utilize degradation products of plants (Botha, 2011; Voříšková and Baldrian, 2013). *Cryptococcus* species might be involved in methane cycling (Takishita et al., 2006) and can be associated with bacterial methylotrophs (*Methylorosula* sp.) (Delavat et al., 2013). For *Trichosporon* methylotrophy has been documented (Kaszycki et al., 2006). The zygomycetous *Mortierella* are widespread and are generalistic saprotrophic fungi with the ability to degrade complex plant material (Dix and Webster, 1995; Kjøller and Struwe, 2002; Hanson et al., 2008; Buée et al., 2009). Since *Cryptococcus* and *Mortierella* are saprotrophic and have a broad range of substrates, a labeling by cross feeding on labeled bacteria might have been an alternative route of carbon assimilation. Nevertheless, an assimilation of methanol cannot be excluded rendering these fungi new candidate methylotrophs that need further experimental attention.

### Influence of an Elevated pH on the Indigenous Methanol-Utilizing Microbiome

Soil is not homogeneous and microscale habitats exist. For example, within few millimetres of soil, pH values can differ up to one pH unit (Or et al., 2007). Thus, we wanted to address if and how the indigenous methanol-utilizers might have been affected to elevated pH values. The total bacterial community was significantly influenced by the increased pH as expected (Supplementary Table S13), and the active methanol-utilizing taxa shifted compared to those under *in situ* pH.

We identified phylotypes of the phyla *Bacteroidetes, Actinobacteria*, and *Betaproteobacteria* as methanol-utilizers at a neutral pH. Among the *Bacteroidetes*, a small number of methylotrophs belonging to *Flavobacteriia* and *Sphingobacteriia* have been reported but none among *Flavobacteriaceae* (Boden et al., 2008; Madhaiyan et al., 2010; Kolb and Stacheter, 2013). Among the *Actinobacteria*, strains of *Leifsonia* are methanol utilizers (Hung et al., 2011). The detected *Betaproteobacteria-*affiliated phylotype was weakly labeled at neutral pH, but was affiliated to the well-known methylotrophic *Methylophilaceae*, of which isolates are neutro- to alcaliphilic (Doronina et al., 2013). Very likely, this phylotype had a lower competitiveness compared with the main bacterial methanol utilizers – i.e., *Beijerinckiaceae*. Although *Methylophilaceae* have been reported to be trophically versatile (Doronina et al., 2013), we did not detect this phylotype in any multi-carbon substrate treatment, which supports the hypothesis that this phylotype thrived *in situ* under unfavorable conditions.

Although the pH shift did not significantly affect the total fungal community (Supplementary Table S14), a reducing effect of the elevated pH on the alpha diversity of active methanol-derived carbon-utilizing fungi was likely based on the observation of a lower number of labeled phylotypes at neutral pH. *Cryptococcus* and *Mortierella* comprise several acid-tolerant species, which likely can also grow under neutral pH conditions (Gross and Robbins, 2000). However, their pH optima seem to be restricted to values < 7, which may explain why *Trichosporon* outcompeted *Cryptococcus* and *Mortierella* species at pH 7 (Gross and Robbins, 2000). The only known methylotrophic *Trichosporon* strain has a growth optimum of pH 8 (Kaszycki et al., 2006). Methylotrophy was not tested at an acidic pH. Since the *Trichosporon* phylotype also incorporated carbon from [^13^C_u_]-glucose and [^13^C_u_]-xylose at pH 4, a broader pH optimum of this taxon of potentially methylotrophic soil yeasts is likely.

### A Methanol-Driven Microbial Food Web in the Investigated Acidic Forest Soil

The detection of several other bacterial and fungal organisms assimilating methanol or methanol-derived carbon suggested a tight trophic link between *Beijerinckiaceae* and other microorganims in the soil. On the one hand *Beijerinckiacea* might have provided carbon sources for several bacterial taxa (i.e., *Acidobacteria, Planctomycetes*, non-methanotrophic *Verrucomicrobia* and *Actinobacteria*), resulting in a weak label in methanol treatments. *Planctomycetes* and *Verrucomicrobia* can degrade extracellular polysaccharides (EPSs) produced by *Beijerinckiaceae* (Wang et al., 2015). Peat-derived *Acidobacteria* were enriched with methanol, glucose, or xylan; but isolation on solely methanol was unsuccessful (Pankratov et al., 2008) suggesting an indirect stimulation. *Edaphobacter aggregans* that grows only in co-culture with *Methylocella silvestris* suggesting methylotrophic *Beijerinckiaceae* as effective suppliers of carbon through EPS formation (Koch et al., 2008). *Actinobacteria* might also utilize other compounds of *Beijerinckiaceae*. *Kineosporia* spp. (OTU_16S_703, *Actinomycetales*) can grow on DNA (Kudo et al., 1998) and therefore both DNA and EPS from *Beijerinckiaceae* might have served as carbon sources. Furthermore, autotrophic growth has been reported for some *Acidimicrobiales* (Johnson et al., 2009) and methanotrophic *Verrucomicrobia* (Khadem et al., 2011; Sharp et al., 2014). Since we detected *Verrucomicrobia* by SIP in treatments that were supplemented with ^13^CO_2_, cross labeling through ^13^CO_2_ was somewhat likely, although we regularly exchanged the headspace atmosphere to minimize this experimental artifact.

On the other hand, soil *Beijerinckiaceae* might feed on methanol released by fungi during lignin decomposition (Messner et al., 2003). An example, for which such a trophic link has been suggested is *Methylovirgula ligni* isolated from a decaying wood that was substantially colonized by a white-rot fungus (Folman et al., 2008; Vorobev et al., 2009). The same might be true for members of the *Actinobacteria* (i.e., *Actinomycetes*) and *Planctomycetes* (i.e., *Phycisphaerae*) of which both members of both phyla might be involved in biopolymer degradation (McCarthy, 1978; Suneetha and Khan, 2010; Bienhold et al., 2013). Thus, it is conceivable that the fungal and bacterial activity increased the local concentration of methanol when degrading plant residues and thus support methylotrophic *Beijerinckiaceae*.

## Conclusion

Our study revealed acidotolerant *Beijerinckiaceae* as the main bacterial methanol sink in a decidous forest soil and highlights their importance for the conversion of methanol in forest soils. These methanol-utilizing *Bacteria* revealed a clear preference for C_1_ compounds that likely enabled them to establish in a complex soil microbiome. The utilization of methanol as sole energy source of various taxa of this family cannot be excluded. We also detected soil yeasts, such as *Cryptococcus* and *Trichosporon*, and saprotrophic *Mortierella*, which suggests that these fungi need to be carefully checked if they are indeed not able to grow on methanol. A putative carbon cross-feeding due to secondarily ^13^C-assimilation especially for fungal species (saprotrophic fungi) cannot be excluded. Nonetheless, the headspace of experimental flasks were reguarly flushed with fresh air and ^12^CO_2_ was added to dilute formed to prevent ^13^CO_2_ labeling through this compound. The observed and discussed aspects of the interaction of *Beijerinckiaceae*, yeasts, fungi, and non-methylotrophic heterotrophic *Bacteria* suggests that these microorganisms are tightly trophically linked through methanol release from plant organs and residues in the surface soil horizons of deciduous temperate forests. Eventually, we provided evidence that soil pH and the substrate spectrum are crucial factors that define the ecological niches of soil methanol utilizers.

## Author Contributions

MM conducted the experiments and wrote the first draft of the manuscript. HH, DKW, GL, and TW analyzed ITS data and contributed to interretation of fungi-associated results. EK supported bioinformatic amplicon analysis and was involved in the interpretation of the data. SK conceptualized the study and wrote the manuscript.

## Conflict of Interest Statement

The authors declare that the research was conducted in the absence of any commercial or financial relationships that could be construed as a potential conflict of interest.
