# Supplementary material for: Acidotolerant Bacteria and Fungi as a Sink of Methanol-Derived Carbon in a Deciduous Forest Soil
Source: Front Microbiol. 2017 Jul 24;8:1361. doi: 10.3389/fmicb.2017.01361 (PMC5523551; doi:10.3389/fmicb.2017.01361)
Supplement: Supplementary file 1 [file Presentation_1.pdf]

Supplementary Information on Materials and Methods

[^{13}C]-substrates for SIP experiment

Filter sterilized 40 mM stock solutions of methanol and multi-carbon substrates (i.e., acetate, glucose, xylose and vanillic acid) were prepared with either the [^{13}C]-isotopologue ('labeled', 99 atom% C) or the [^{12}C]-isotope (i.e., 'unlabeled', natural abundance of ^{13}C). All multi-carbon substrate stock solutions also included 40 mM [^{12}C]-methanol.

All isotopologues were fully labeled (i.e., [$^{13}C_u$]), except for vanillic acid, where only the aromatic ring carbon was [^{13}C]-labeled (i.e., [$^{13}C_{1-6}$]); methyl and carboxyl groups possessed [^{12}C]-carbon. For the CO_2 incubations, either gaseous [^{13}C]-CO_2 ('labeled', 99 atom% C; <3 atom% ^{18}O) or [^{12}C]-CO_2 was used. [^{13}C]-isotopes were purchased from Campro Scientific (Berlin, Germany) and Sigma-Aldrich (Steinheim, Germany), [^{12}C]-isotopes were purchased from Roth (Karlsruhe, Germany), Sigma-Aldrich (Steinheim, Germany), Panreac Applichem (Darmstadt, Germany), Merck (Darmstadt, Germany) and Rießner (Lichtenfels, Germany).

Substrate SIP experiment

Soil slurries were prepared by mixing 50 g of sieved soil (fresh weight) with 40 ml of trace element solution (in 1 L of sterile water: HCl (i.e. fuming HCl, 37%), 50 µM; $FeCl_2$ x 4 H_2O, 5 µM; $ZnCl_2$, $MnCl_2$ x 2 H_2O, $CoCl_2$ x 6 H_2O, 50 µM; Na_2MoO_4 x 2 H_2O, 0.15 µM; H_3BO_3, $NiCl_2$ x 6 H_2O, 0.10 µM; $CuCl_2$ x 2 H_2O, 0.01 µM; after Whittenbury *et al.*, 1970). Slurries were initially homogenized by hand shaking. Soil slurry incubations were performed in duplicate for each approach (control, ^{12}C or ^{13}C) in a sterile screw-capped butyl-rubber-stopped 0.5 L flask (Glasgerätebau Ochs, Bovenden, Germany; Müller + Krempel, Bülach, Switzerland) on an end-over-end shaker at 20°C in the dark. Oxic atmosphere was offered by a large gas phase inside the flasks (i.e., a thin slurry layer onto the flask inner surface) and by daily opening, allowing for acclimatization before re-sealing. In addition, the O_2 concentrations were monitored.

Substrates (i.e., methanol, acetate, and sugars; 1 ml) and CH_4 were supplemented daily to a final concentration of 1 mM and 200 ppm, respectively. Vanillic acid was supplemented if it was no longer detectable. Slurry aliquots were taken before and after supplementation and kept at -20°C or -80°C for analytical and molecular analyzes, respectively. Unsupplemented control slurry incubations served as methanol control treatments and lacked any substrate treatment besides CH_4. The additional supplemented aqueous volume per substrate pulse in the substrate treatments (i.e., 1 ml) was compensated through supplementation of the same volume of trace element solution (i.e., 1 ml) to the control. Because multi-carbon substrate treatments were additionally supplemented with methanol (1 mM, final concentration), methanol treatments served as multi-carbon substrate controls. CO_2 incubations were supplemented with 10% CO_2 in the headspace (approx. 7 mM total amount) and opened if the O_2 concentration was below 10%. The purpose of CO_2 treatments was (i) to analyze cross-feeding effects and (ii) to identify potential CO_2-assimilating taxa.

A summarizing overview of the experimental set-up of both SIP experiments (substrate SIP and pH shift SIP experiment) is given in Figure S1.

Chemical analyzes – HPLC and GC measurements

The pH was determined using a pH electrode (InLab R422, Mettler Toledo GmbH, Gießen, Germany) and a pH meter (WTW pH 330, Wissenschaftlich-Technische Werkstätten, Weilheim, Germany). Gases were measured by gas chromatography using TCD (O_2, CO_2) and FID (CH_4) as described elsewhere (Küsel & Drake, 1995). The total amount of gases (i.e., gases in the headspace and liquid phase) was calculated using the ideal gas law and considering the actual temperature, pressure, slurry volume and solubility of gases (Blachnik, 1998). The amount of [^{13}C]-CO_2 was determined with a GC-MS (see details below).

The conversion of supplemented substrates was monitored in a filtered aqueous supernatant of slurry aliquots by high-performance liquid chromatography (HPLC) using RID (1090 series II, Hewlett Packard, Paolo Alto, CA, USA; Küsel & Drake, 1995) and DAD (1200 Series, Agilent Technologies, Santa Clara, CA, USA; Liu *et al.*, 2011). Online absorption spectra from 210 to 350 nm were used to confirm the purity, and the absorption at 264 nm was used for the quantification of vanillic acid.

Chemical analyzes – GC-MS of [^{13}C]-CO_2

For analyzes of the [^{13}C]-CO_2 concentrations in the gas samples, a GC-MS analysis was performed using a Perkin–Elmer GC Clarus 600 system with an Rtx®-1 capillary column (60 m x 320 µM). For GC-MS detection, an electron ionization system was operated with an ionization energy of 70 eV. Mass spectra were taken from 14 to 70 Da. Helium was used as carrier gas at a constant flow of 300 kPa, and an injection volume of 10 µl (split ratio 10:1) was administered manually using a gastight syringe. Each sample was measured five times. The total amount of [^{12}C]-CO_2 and [^{13}C]-CO_2 was analyzed by the extraction of m/z values 44 and 45 followed by peak integration. The peak areas were corrected with [^{12}C]-CO_2 and [^{13}C]-CO_2 indoor air values, and finally, the ratio of the m/z values 45/44 was calculated.

Calculation of the carbon recovery rates of [^{13}C]-CO_2

The carbon recovery for [^{13}C]-CO_2 was calculated by calculating the percentage of the formed [^{13}C]-CO_2 in relation to the total amount of [^{13}C]-isotopologues supplemented for each timepoint.The following equation was applied:

$$^{13}CR = \frac{100\%}{c_{13_{isotopologue}}} x\ c_{13_{CO_2}}$$

^{13}CR = carbon recovery of the supplemented ^{13}C

$c_{13_{isotopologue}}$ = concentration of the supplemented ^{13}C isotopologue

$c_{13_{CO_2}}$ = concentration of the detected $^{13}CO_2$

DNA – SIP, isopycnic centrifugation and formation of different comparable gradients

DNA SIP was performed according to Neufeld *et al.* (2007). Equally pooled DNA from the t_0, ^{12}C and ^{13}C treatments (i.e., 5 µg (t_0 and substrates treatments) up to 10 µg (methanol treatments)) was added to each CsCl-containing gradient (buoyant density 1.732±0.0006

g/ml) and filled into OptiSeal Tubes (Beckmann, Fullerton, CA, USA). Isopycnic centrifugation was performed (44 100 rpm / ~ 177 000 g_{av} at 20°C for 40 hours; rotor VTi65.2; Beckmann, Fullerton, CA, USA) to separate DNA by its buoyant densities (BD).

Due to the number of different gradients, independent centrifugation runs were conducted. Comparability was still given by gradient solutions with a density of 1.732 ± 0.0006 g/ml used for all runs and the isopycnic centrifugation of corresponding DNA from [^{12}C]- and [^{13}C]-treated samples of the corresponding treatments (i.e., DNA derived from the [^{12}C]- and [$^{13}C_1$]-methanol treatments was subjected to the same centrifugation run). For the substrate SIP experiment, gradients loaded with DNA for bacterial analyzes ranged in general from 1.750 ± 0.003 g/ml to 1.697 ± 0.007 g/ml and those for fungal analyzes from 1.750 ± 0.004 g/ml to 1.696 ± 0.004 g/ml. For the pH SIP experiment, the gradients ranged in general from 1.744 ± 0.004 g/ml to 1.699 ± 0.004 g/ml.

The gradients were separated into 10 fractions (450 µl each), and the buoyant density of each fraction was determined by weighing at 20°C. DNA was precipitated with glycogen (10 mg/ml) and polyethylenglycol and quantified.

According to the reported BDs for non-labeled and fully labeled DNA (i.e., 1.69 up to 1.725 g/ml and 1.76 g/ml, respectively (Carter *et al.*, 1983; Lueders *et al.*, 2004)), fractions 1 to 10 were separately pooled into ‘heavy’ (fractions with a buoyant density ≥1.730 g/ml), ‘middle’ (fractions with a buoyant density between 1.730 and 1.715 g/ml), and ‘light’ (fractions with a buoyant density ≤1.715 g/ml) fractions.

Barcoded amplicon pyrosequencing of 16S rRNA genes, *mxaF*, *pmoA* and ITS

According to Berry *et al.* (2011), a two-step PCR approach was conducted and included a PCR with the ‘conventional’ primer set (step-1-PCR) followed by a PCR with barcoded primers (step-2-PCR). Barcoded primers (i.e., conventional primers including an oligonucleotide at the 5’-end) were necessary to re-identify amplicons in multiplex sequencing and to create different pyrosequencing libraries. In general, all PCR reactions (i.e., step-1-PCR and step-2-PCR) were conducted in duplicate.

Bacterial 16S rRNA gene sequences were amplified with the primer pair 341f/785-805r (Muyzer *et al.*, 1998; Herlemann *et al.*, 2011), the marker gene *mxaF* was amplified with the primer pairs “mxaF1” (1003f/1555r (McDonald & Murrell, 1997; Neufeld *et al.*, 2007)) and “mxaF2” (mxaF_for/mxaF_rev (Moosvi *et al.*, 2005)), and the marker gene *pmoA* was amplified with the primer pair A189f/A682r (Holmes *et al.*, 1995). For detailed information on the primer sequences, see Table S1.

The amplification of *mxaF* fragments with “mxaF1” in step-1-PCR for Substrate SIP experiment was insufficient and led to a second amplification with “mxaF2” targeting a region within the previously amplified segment. Amplicons from the first PCR were purified with OMEGA Bio-Tek (Norcross, GA, USA) and re-amplified in a nested PCR (for the reaction mixture, see Table S2, and for the thermo protocol, see Table S3). PCR products from this nested PCR were templates for the step-2-PCR with barcoded primers.

The amplification of *pmoA* was insufficient and resulted in only weak bands for t_0 samples and the corresponding L fractions. Thus, the barcoding (i.e., step-2-PCR) of *pmoA* was not conducted.

In all DNA extracts of the pH SIP experiment, huge amounts of humic acids were detected, and PCR inhibitory effects were still present after isopycnic centrifugation. Thus, step-1-PCR

was conducted with different PCR reagents showing increased inhibitor tolerance than the reagents for the substrate SIP experiment (for further details see Table S2).

The step-1-PCR products (i.e., 16S rRNA gene and *mxaF* fragments) were barcoded in step-2-PCR (for further details, see Table S3), and the amplicons were subsequently gel-purified with the montage Gel Extraction Kit (Millipore GmbH, Schwalbach, Germany) and equimolarly pooled corresponding to their original sample. The amplicon pools were treated with PreCRepair MIX (NEB, Frankfurt am Main, Germany) according to the manufacturer's protocol to reduce or eliminate possible DNA damage that might have occurred during the gel purification or amplicon storage. The repaired amplicon pools were subsequently purified, concentrated by vaporizing (still resolved in 10 mM TRIS, pH 8) and then pyrosequenced at the Göttingen Genomics Laboratory using Roche GS-FLX 454 Sequencer and GSL FLX Titanium series reagents according to the manufacturer's protocol (Roche Diagnostics GmbH, Mannheim, Germany) as described elsewhere (Stacheter *et al.*, 2013).

Fungal ITS gene fragments were amplified with the primer pair ITS1F/ITS4 (White *et al.*, 1990; Gardes & Bruns, 1993) (for primer sequences, see Table S1). In general, all PCR reactions were conducted in triplicate. The amplification of ITS in samples derived from the substrate SIP experiment followed two different strategies. ITS gene fragments of pooled DNA of the 'middle' and 'light' fractions were amplified in one PCR step (similar to step-1-PCR) with an ITS fusion primer pair. Fusion primers possess an adaptor sequence and barcodes at the 5'-end (for general fusion primer sequences, see Table S4). The ITS gene fragments of pooled DNA of the 'heavy' fractions were amplified in the two-step PCR approach (Berry *et al.*, 2011) with the conventional primer pair in step-1-PCR and the fusion primer pair in step-2-PCR (for the reaction mixture, see Table S5; for the thermo protocol, see Table S6). In accordance with the amplification of bacterial genes, the amplification of the ITS fragments of the pH SIP experiment was also conducted in a two-step PCR approach but with different PCR reagents than for the substrate SIP experiment caused by inhibitory effects of humic acids (for further details see Table S5).

Before pyrosequencing, all ITS amplicon-replicates were pooled, and fragments between 400 bp and 1200 bp were gel-purified with QIAquick Gel Extraction Kit (Qiagen, Hilden, Germany) on a Dark Reader Transilluminator (Clare Chemical Research Inc, Dolores, CO, USA) using blue light instead of UV light. Thus, no amplicon damage was assumed, making the DNA repair kit redundant. Amplicons were equimolarly pooled and sequenced at the Department of Soil Ecology (UFZ, Halle, Germany) as previously described (Wubet *et al.*, 2012).

Sequence filtering, clustering of amplicon pyrosequencing reads and analyzes

Recovered reads of bacterial genes were trimmed to 446 nt (for 16S rRNA gene amplicons) and 461 nt (for *mxaF* amplicons) so that the reverse primer sequence was mostly removed. Amplicon pyrosequencing errors were corrected using ACACIA, i.e., homopolymer error-correction and low quality reads were discarded (Bragg *et al.*, 2012). Potential chimeric 16S rRNA gene sequences were filtered out using the UCHIME algorithm implemented in USEARCH and the latest RDP Gold database for high-quality 16S rRNA gene reference sequences (Edgar *et al.*, 2011). Before sequence clustering, initial barcode sequences were modified to re-assign amplicons (see Table S6 for details). Using JAguc v2.1 (Nebel *et al.*, 2011), sequences were clustered into operational taxonomic units (OTUs) using a pairwise sequence alignment before creating a distance matrix and clustering with the average

similarity method. Only sequences with the correct forward primer sequence were further analyzed. OTUs of 16S rRNA gene sequences were clustered at the family level with a 90.1% pairwise similarity cut-off value (Yarza *et al.*, 2010), and *mxaF* OTUs were clustered with a cut-off value of 90%. The *mxaF* cut-off was higher than previously reported (Stacheter *et al.*, 2013) to obtain a greater diversity while still exhibiting a relatively constant number of retrieved OTUs (Figure S2). An overview of the total number of sequences derived from pyrosequencing and after clustering is given in Table S7. The phylogenetic affiliation of ribosomal sequences was determined by a local nucleotide BLAST using the latest NCBI GenBank release (GenBank release 209, 14.08.2015). Affiliation was verified by the manual BLAST of the OTU's representative sequences and by a phylogenetic tree that was generated using MEGA version 6.06 (Tamura *et al.*, 2013). The affiliation of *mxaF* OTUs was determined by a manual BLAST and phylogenetic trees.

A more detailed resolution on species level was conducted for OTU_{16S}438. Therefore the filtered dataset of 16S rRNA genes (used in the previously conducted JAguc analysis, possessing the 5 nt-barcodes, see Table S6) and all sequences of OTU_{16S}438 (with an artificial barcode, 5'-AAAAA-3') were merged and subsequently re-clustered. Raw fastq reads were joined and then demultiplexed using QIIME (Caporaso et al., 2010). Quality filtering, length truncating and converting the fastq files to fasta files as well as dereplication, abundance sort and discard of singletons were done with USEARCH v7 (Edgar et al., 2010). Operational taxonomic unit (OTU) clustering was carried out with UPARSE within USEARCH v7. Chimeras were removed using UCHIME within USEARCH v7 and the 16S rRNA gene Gold database (http://drive5.com/uchime/gold.fa). Taxonomy was assigned and sequences aligned with QIIME using Greengenes for reference-based assignment and alignment, respectively (DeSantis et al., 2006).

Fungal ITS reads were demultiplexed and quality trimmed using MOTHUR (Schloss *et al.*, 2009). The reads that met the following criteria were further analyzed: holding one of the expected barcodes (1 mismatch allowed; for the barcode sequences, see Table S8), a sequenced forward primer (i.e., ITS4, 4 mismatches allowed), with a minimum length of 355 nt, a minimum average quality of a 29 Phred score over the 355 first nucleotides, a maximum homopolymer length of 8 nt, and no ambiguous nucleotides. The reads were trimmed to their 355 first nucleotides to avoid low-quality ends and length sorting in the following clustering step. The normalized reads (1503 counts per sample) were checked for chimeric sequences using UCHIME (Edgar *et al.*, 2011) as implemented in MOTHUR. Dereplicated sequences were sorted by decreasing abundance and were clustered into OTUs using CD-HIT-EST (Fu *et al.*, 2012) at a 97% pairwise similarity cut-off value. Low-abundant OTUs with 3 or fewer reads were removed because they potentially originated from artificial sequences (Kunin *et al.*, 2010). Representative OTU sequences were classified against the dynamic UNITE database (v7 release 01.08.2015; Kõljalg *et al.*, 2013) using the MOTHUR implementation of Wang et al. (2007) classifier. The sequences that could not be assigned further than to the kingdom Fungi were classified for a second time against a previous database including non-fungal ITS sequences retrieved from GenBank (release 207, accessed on 06.05.2015; Benson *et al.*, 2008) to detect and remove non-fungal sequences. Subsequently, the remaining sequences assigned only to the Fungi kingdom were classified against the full UNITE database to improve the taxonomic affiliation. In addition, the reference sequences of the selected OTUs (representative sequence) were manually identified by 'massBLASTer analyzes' of the UNITE database to confirm affiliation. An overview of the total number of sequences derived from pyrosequencing and after clustering is given in Table S7.

Quantification of 16S rRNA genes, *mxaF* and *mmoX* genes in pH shift SIP experiment-derived samples (qPCR)

The gene fragments were quantified in duplicate (for details of reaction mixtures, see Table S9) with an iQ5 Real-Time qPCR cycler (BioRad, Munich, Germany) with specific primer sets for Bacteria and marker genes *mxaF* and *mmoX* (see Table S10). The internal standards were prepared by the specific amplification of targeted gene fragments derived from the genomic DNA of *Escherichia coli* JM109 (for 16S rRNA gene), *Methylobacterium extorquens* CM4 (for *mxaF*) and *Methylocella capsulatus* BATH (for *mmoX*) (for the reaction mixture see S9; for the thermal protocol, see S11). Amplicons were gel-purified with the montage Gel Extraction Kit (Millipore GmbH, Schwalbach, Germany) and cloned into the pJET1.2 vector System (ThermoScientific, Waltham, MA, USA). Clone inserts with expected size were multiplied (for the reaction mixture, see S9; for the thermal protocol, see S11), gel-purified, quantified with QUANT-iT-Pico Green (Invitrogen, Carlsbad, CA, USA) and stored as stock solutions (10^{-9} target genes per 5 µl). Standard curves were prepared from serially diluted stock solutions ranging from 10^{-7} to 10^{-1} target genes per qPCR reaction. In every qPCR run, distilled water (DNase/RNase free, from Gibco (Paisley, United Kingdom)) served as a negative control to assure no contamination. Slightly modified established qPCR assays were applied (for details on the assays, see Table S12).

According to Degelmann *et al.* (2010), all qPCR measurements were inhibitor corrected because coextracted humic acids were obvious in pH-SIP DNA extracts, and inhibition was well recorded (Tsai & Olson, 1992; Wilson, 1997; von Wintzingerode *et al.* 1997; Watson & Blackwell, 2000; Rådström *et al.,* 2004). In brief, DNA templates were spiked with artificial DNA so that 10^{-4} target molecules per qPCR reaction were expected. The multiple cloning site region of plasmid pCR 2.1-TOPO (Invitrogen, Karlsruhe, Germany), flanked by M13 priming sites without any insert, served as artificial DNA. qPCR assays were conducted (for details, see Table S9 and S12), inhibition factors were calculated, and gene-specific qPCR assays were corrected.

Community analyzes and statistics

All filtered and clustered amplicon pyrosequencing datasets (i.e., libraries of 'light', 'middle' and 'heavy' fractions) of ^{12}C and ^{13}C treatments were combined into an entire dataset for each treatment. Community analyzes were always based on the family level for phylotypes and a 90% similarity cut-off for *mxaF* genotypes. All statistical analyzes were performed using PAST version 1.85 and version 3.08 (Hammer *et al.*, 2001).

References

Benson, D.A., Karsch-Mizrachi, I., Lipman, D.J., Ostell, J., and Wheeler, D.L. (2008). GenBank. *Nucleic Acids Res* 36, D25–D30. doi: 10.1093/nar/gkm929

Berry, D., Ben Mahfoudh, K., Wagner, M., and Loy, A. (2011). Barcoded Primers used in multiplex amplicon Pyrosequencing Bias amplification. *Appl Environ Microbiol* 77, 7846–7849. doi: 10.1128/AEM.05220-11

Blachnik, R. (1998). D'Ans Lax Taschenbuch für Chemiker und Physiker, Vol. 3, 4th ed. Springer Verlag: Berlin.

Bragg, L., Stone, G., Imelfort, M., Hugenholtz, P., and Tyson, G.W. (2012). Fast, accurate error-correction of amplicon pyrosequences using Acacia. *Nat Methods* 9, 425-426. doi:10.1038/nmeth.1990

Caporaso, J.G., Kuczynski, J., Stombaugh, J., Bittinger, K., Bushman, F.D., Costello, E.K., *et al.* (2010). QIIME allows analysis of high-throughput community sequencing data. *Nat Methods* 7, 335-336. doi: 10.1038/nmeth.f.303. Epub 2010 Apr 11.

Carter, C., Britton, V.G., and Haff, L. (1983). CsTFA: a centrifugation medium for nucleic acid isolation. *BioTechniques* 1, 142–147.

Degelmann, D.M., Borken, W., Drake, H.L., and Kolb, S. (2010). Different atmospheric methane-oxidizing communities in European beech and Norway spruce soils. *Appl Environ Microbiol* 76, 3228–3235. doi: 10.1128/AEM.02730-09

DeSantis, T.Z., Hugenholtz, P., Larsen, N., Rojas, M., Brodie, E.L., Keller, K., et al. (2006). Greengenes, a chimera-checked 16S rRNA gene database and workbench compatible with ARB. *Appl Environ Microbiol* 72, 5069–5072. doi: 10.1128/AEM.03006-05

Edgar, R.C., Haas, B.J., Clemente, J.C., Quince, C., and Knight, R. (2011). UCHIME improves sensitivity and speed of chimera detection. *BioInformatics* 27, 2194–2200. doi: 10.1093/bioinformatics/btr381

Fu, L., Niu, B., Zhu, Z., Wu, S., and Li, W. (2012). CD-HIT: accelerated for clustering the next-generation sequencing data. *BioInformatics* 28, 3150–3152. doi:10.1093/bioinformatics/bts565

Gardes, M., and Bruns, T.D. (1993). ITS primers with enhanced specificity for basidiomycetes - application to the identification of mycorrhizae and rusts. *Mol Ecol* 2, 113–118. doi: 10.1111/j.1365-294X.1993.tb00005.x

Hammer, Ø., Harper, D.A.T., and Ryan, P.D. (2001). PAST: palaeontological statistics software package for education and data analysis. *Palaeontol Electron* 4, 1–9.

Herlemann, D.P., Labrenz, M., Jürgens, K., Bertilsson, S., Waniek, J.J., and Andersson, A.F. (2011). Transitions in bacterial communities along the 2000 km salinity gradient of the Baltic Sea. *ISME J* 5,1571–1579. doi: 10.1038/ismej.2011.41

Holmes, A.J., Costello, A., Lidstrom, M.E., and Murrell, J.C. (1995). Evidence that particulate methane monooxygenase and ammonia monooxygenase may be evolutionarily related. *FEMS Microbiol Lett* 132, 203-208.

Kõljalg, U., Nilsson, R.H., Abarenkov, K., Tedersoo, L., Taylor, A.F., Bahram, M. *et al.* (2013). Towards a unified paradigm for sequence-based identification of fungi. *Mol Ecol* 22, 5271–5277. doi: 10.1111/mec.12481

Kunin, V., Engelbrektson, A., Ochman, H., and Hugenholtz, P. (2010). Wrinkles in the rare biosphere: pyrosequencing errors can lead to artificial inflation of diversity estimates. *Environ Microbiol* 12, 118–123. doi: 10.1111/j.1462-2920.2009.02051.x

Küsel, K., and Drake, H.L. (1995). Effects of environmental parameters on the formation and turnover of acetate by forest soils. *Appl Environ Microbiol* 61, 3667–3675.

Liu, Y.J., Liu, S.J., Drake, H.L., and Horn, M.A. (2011). Alphaproteobacteria dominate active 2-methyl-4- chlorophenoxyacetic acid herbicide degraders in agricultural soil and drilosphere. *Environ Microbiol* 13, 991–1009. doi: 10.1111/j.1462-2920.2010.02405.x

Lueders, T., Manefield, M., and Friedrich, M.W. (2004). Enhanced sensitivity of DNA- and rRNA-based stable isotope probing by fractionation and quantitative analysis of isopycnic centrifugation gradients. *Environ Microbiol* 6, 73–78. doi: 10.1046/j.1462-2920.2003.00536.x

McDonald, I.R., and Murrell, J.C. (1997). The methanol dehydrogenase structural gene mxaF and its use as a functional gene probe for methanotrophs and methylotrophs. *Appl Environ Microbiol* 63, 3218–3224.

Moosvi, S.A., McDonald, I.R., Pearce, D.A., Kelly, D.P., and Wood, A.P. (2005). Molecular detection and isolation from Antarctica of methylotrophic bacteria able to grow with methylated sulfur compounds. *Syst Appl Microbiol* 28, 541-554.

Muyzer, G., Brinkhoff, T., Nuebel, U., Santegoeds, C., Schaefer, H., and Waver, C. (1998). “Denaturing gradient gel electrophoresis (DGGE) in microbial ecology.” In: Akkermans, A.D.L, van Elsas, J.D., and de Bruijn, F.J. (eds). Molecular Microbial Ecology Manual. Kluwer Academic Publishers: Dordrecht, pp 1–27.

Nebel, M.E., Wild, S., Holzhauser, M., Hüttenberger, L., Reitzig, R., Sperber, M. *et al*. (2011). Jaguc—a software package for environmental diversity analyzes. *J Bioinform Comput Biol* 9, 749–773.

Neufeld, J.D., Vohra, J., Dumont, M.G., Lueders, T., Manefield, M., Friedrich, M.W. *et al.* (2007). DNA stable-isotope probing. *Nat Protoc* 2, 860-866. doi: doi:10.1038/nprot.2007.109

Rådström, P., Knutsson, R., Wolffs, P., Lövenklev, M., and Löfström, C. (2004). Pre-PCR processing: strategies to generate PCR-compatible samples. *Mol Biotechnol* 26, 133–146. doi:10.1385/MB:26:2:133

Schloss, P.D., Westcott, S.L., Ryabin, T., Hall, J.R., Hartmann, M., Hollister, E.B. *et al.* (2009). Introducing mothur: open-source, platform-independent, community-supported software for describing and comparing microbial communities. *Appl Environ Microbiol* 75, 7537–7541. doi: 10.1128/AEM.01541-09

Stacheter, A., Noll, M., Lee, C.K., Selzer, M., Glowik, B., Ebertsch, L. *et al.* (2013). Methanol oxidation by temperate soils and environmental determinants of associated methylotrophs. *ISME J* 7, 1051-1064. doi:10.1038/ismej.2012.167

Tamura, K., Stecher, G., Peterson, D., Filipski, A., and Kumar, S. (2013). MEGA6: molecular evolutionary genetics analysis version 6.0. *Mol Biol Evol* 30, 2725-2729. doi: 10.1093/molbev/mst197

Tsai, Y.L., and Olson, B.H. (1992). Rapid method for separation of bacterial DNA from humic substances in sediments for polymerase chain reaction. *Appl Environ Microbiol* 58, 2292–2295.

von Wintzingerode, F., Göbel, U.B., and Stackebrandt, E. (1997). Determination of microbial diversity in environmental samples: pitfalls of PCR-based rRNA analysis. *FEMS Microbiol Rev* 21, 213–229.

Wang, Q., Garrity, G.M., Tiedje, J.M., and Cole, J.R. (2007). Naive Bayesian classifier for rapid assignment of rRNA sequences into the new bacterial taxonomy. *Appl Environ Microbiol* 73, 5261–5267. doi: 10.1128/AEM.00062-07

Watson, R.J., and Blackwell, B. (2000). Purification and characterization of a common soil component which inhibits the polymerase chain reaction. *Can J Microbiol* 46, 633–642. doi: 10.1139/w00-043

White, T.J., Bruns, T.D., Lee, S.B., and Taylor, J.W. (1990). “Amplification and direct sequencing of fungal ribosomal RNA genes for phylogenetics”. In: Innis, M.A., Gelfand, D.H., Sninsky, J.J., White, T.J. (eds). PCR Protocols - a Guide to Methods and Applications. Academic Press: Zürich, pp 315–322.

Whittenbury, R., Philips, K. C., and Wilkinson J. F. (1970). Enrichment, isolation, and some properties of methane-utilizing bacteria. *J Gen Microbiol* 61, 205-218. doi: 10.1099/00221287-61-2-205

Witthoff, S., Mühlroth, A., Marienhagen, J., Bott, M. (2013). C1 Metabolism in *Corynebacterium glutamicum*: an Endogenous Pathway for Oxidation of Methanol to Carbon Dioxide. *Appl Environ Microbiol* 79, 6974–6983. doi:10.1128/AEM.02705-13

Wilson, I.G. (1997). Inhibition and facilitation of nucleic acid amplification. *Appl Environ Microbiol* 63, 3741–3751.

Wubet, T., Christ, S., Schöning, I., Boch, S., Gawlich, M., Schnabel, B. *et al.* (2012). Differences in Soil fungal communities between European beech (Fagus sylvatica L.) Dominated Forests Are related to Soil and understory vegetation. *PLOS ONE* **7**:e47500. doi: 10.1371/journal.pone.0047500.

Yarza, P., Ludwig, W., Euzéby, J., Amann, R., Schleifer, K.-H., Glöckner, F.O. *et al.* (2010). Update of the All-species living tree project based on 16S and 23S rRNA sequence analyzes. *Syst Appl Microbiol* 33, 291–299. doi: 10.1016/j.syapm.2010.08.001

List of supplementary Figures

Figure S1. Overview on the experimental set-up of both SIP experiments.

Figure S2. Correlation between the number of detected genotypes and the nucleotide sequence similarities of *mxaF* gene sequences.

List of supplementary Tables

Table S1. Primer sequences of the ‘conventional’ primers used to amplify 16S rRNA gene, *mxaF* and ITS gene fragments in Step-1-PCR for amplicon pyrosequencing.

Table S2. Composition of the reagents for PCR reactions in the two-step approach PCR of barcoded amplification for bacterial gene fragments (16S rRNA gene, mxaF, pmoA).

Table S3. PCR programs to amplify 16S rRNA gene, *mxaF*, *pmoA* and ITS gene fragments in step-1-PCR and step-2-PCR of barcoded amplification.

Table S4. Structure of the fusion primers used for amplicon pyrosequencing.

Table S5. Composition of the reagents for PCR reactions in the two-step approach PCR of barcoded amplification for fungal gene fragments (ITS).

Table S6. Sequences of the barcodes used to identify individual samples in the pyrosequencing amplicon libraries for 16S rRNA gene and mxaF sequences of both SIP experiments.

Table S7. Number of all sequences obtained from the pyrosequencing amplicon libraries of both SIP experiments.

Table S8. Sequences of the barcodes used to identify individual samples in the pyrosequencing amplicon libraries for ITS gene fragments.

Table S9. Composition of the reagents for PCR reactions of the qPCR standard preparation, cloning and qPCR assays.

Table S10. Primer sequences of the primers used for qPCR and to prepare the qPCR standards.

Table S11. PCR programs to amplify gene fragments for qPCR standards.

Table S12. Modified qPCR programs to amplify 16S rRNA gene, *mxaF*, *mmoX* and artificial DNA (Inhibit).

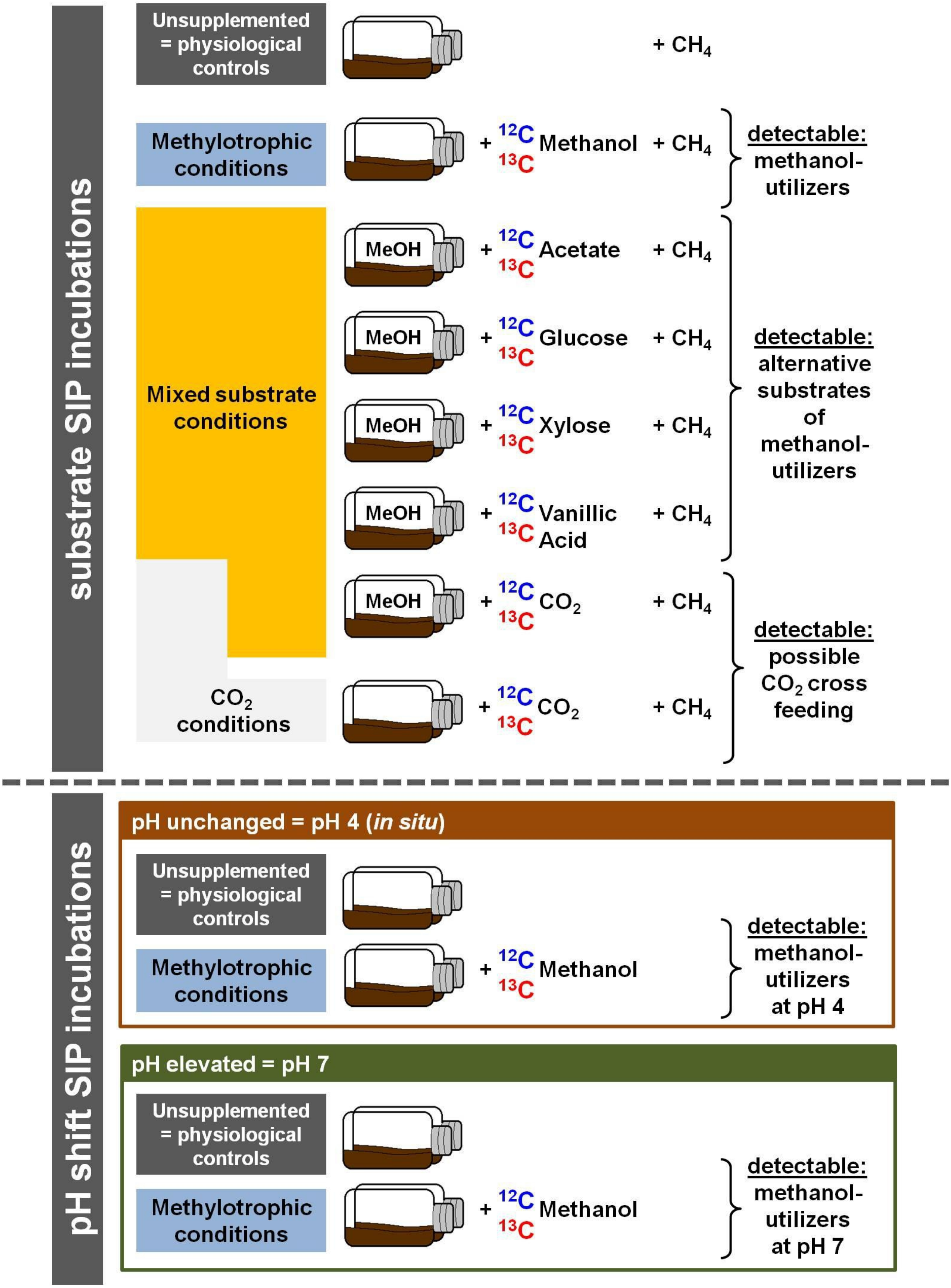

Figure S1. Overview on the experimental set-up of both SIP experiments. Substrate SIP and pH shift SIP experiment were conducted independent from each other with different soil samples as starting material. In the substrate SIP experiment several substrates (C_1 and C_n) were supplemented as ^{12}C- (blue font) or ^{13}C-isotopologue (red font). Additionally, [^{12}C]-methane (black font) was supplemented. In the pH shift SIP experiment solely [^{12}C]- or [^{13}C]-methanol was supplemented. For all pH 4 treatments (i.e. *in situ*) no adjustment of the soil slurry pH was conducted; for pH 7 treatments the initial soil slurry pH was elevated.

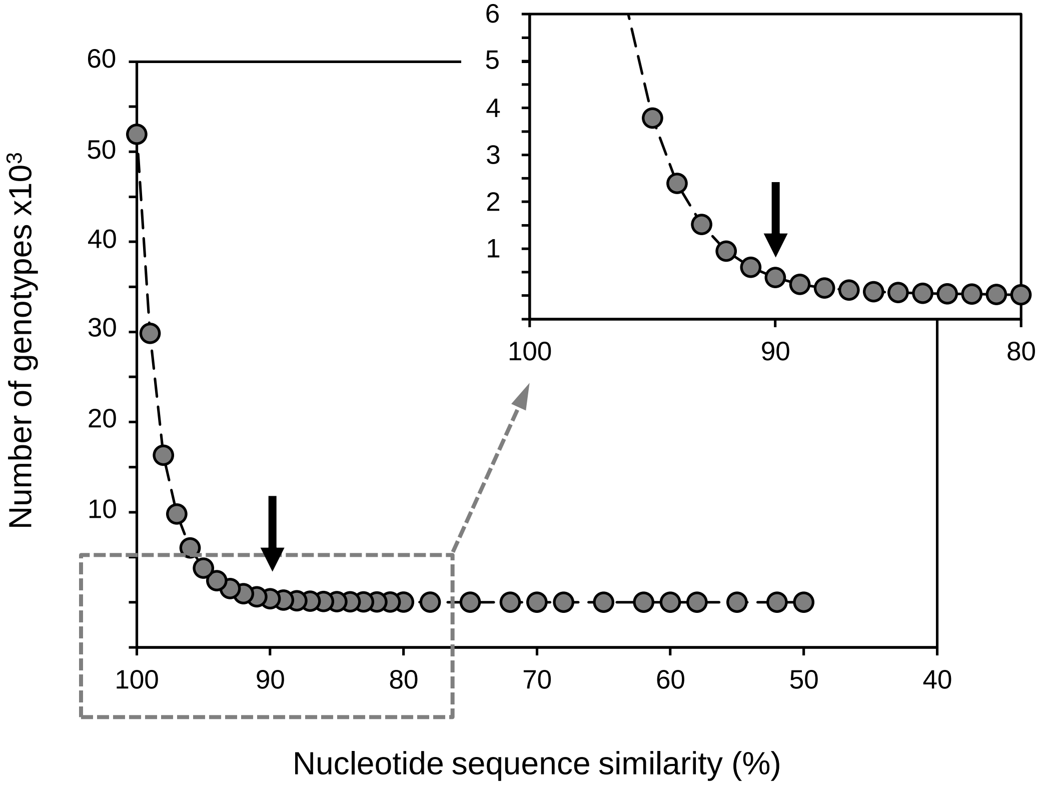

Figure S2. Correlation between the number of detected genotypes and the nucleotide sequence similarities of *mxaF* gene sequences. Shown is the result of the analysis of all detected *mxaF* gene sequences (i.e. 113 689 sequences) in all pyrosequencing amplicon libraries of both SIP experiments. Arrows indicate the similarity threshold value of 90% chosen for clustering and further analyses. Inset focuses on a sequence similarity range between 80% and 100%.

List of supplementary Figures

Figure S1. Overview on the experimental set-up of both SIP experiments.

Figure S2. Correlation between the number of detected genotypes and the nucleotide sequence similarities of *mxaF* gene sequences.

List of supplementary Tables

Table S1. Primer sequences of the ‘conventional’ primers used to amplify 16S rRNA gene, *mxaF* and ITS gene fragments in Step-1-PCR for amplicon pyrosequencing.

Table S2. Composition of the reagents for PCR reactions in the two-step approach PCR of barcoded amplification for bacterial gene fragments (16S rRNA gene, mxaF, pmoA).

Table S3. PCR programs to amplify 16S rRNA gene, *mxaF*, *pmoA* and ITS gene fragments in step-1-PCR and step-2-PCR of barcoded amplification.

Table S4. Structure of the fusion primers used for amplicon pyrosequencing.

Table S5. Composition of the reagents for PCR reactions in the two-step approach PCR of barcoded amplification for fungal gene fragments (ITS).

Table S6. Sequences of the barcodes used to identify individual samples in the pyrosequencing amplicon libraries for 16S rRNA gene and mxaF sequences of both SIP experiments.

Table S7. Number of all sequences obtained from the pyrosequencing amplicon libraries of both SIP experiments.

Table S8. Sequences of the barcodes used to identify individual samples in the pyrosequencing amplicon libraries for ITS gene fragments.

Table S9. Composition of the reagents for PCR reactions of the qPCR standard preparation, cloning and qPCR assays.

Table S10. Primer sequences of the primers used for qPCR and to prepare the qPCR standards.

Table S11. PCR programs to amplify gene fragments for qPCR standards.

Table S12. Modified qPCR programs to amplify 16S rRNA gene, *mxaF*, *mmoX* and artificial DNA (Inhibit).

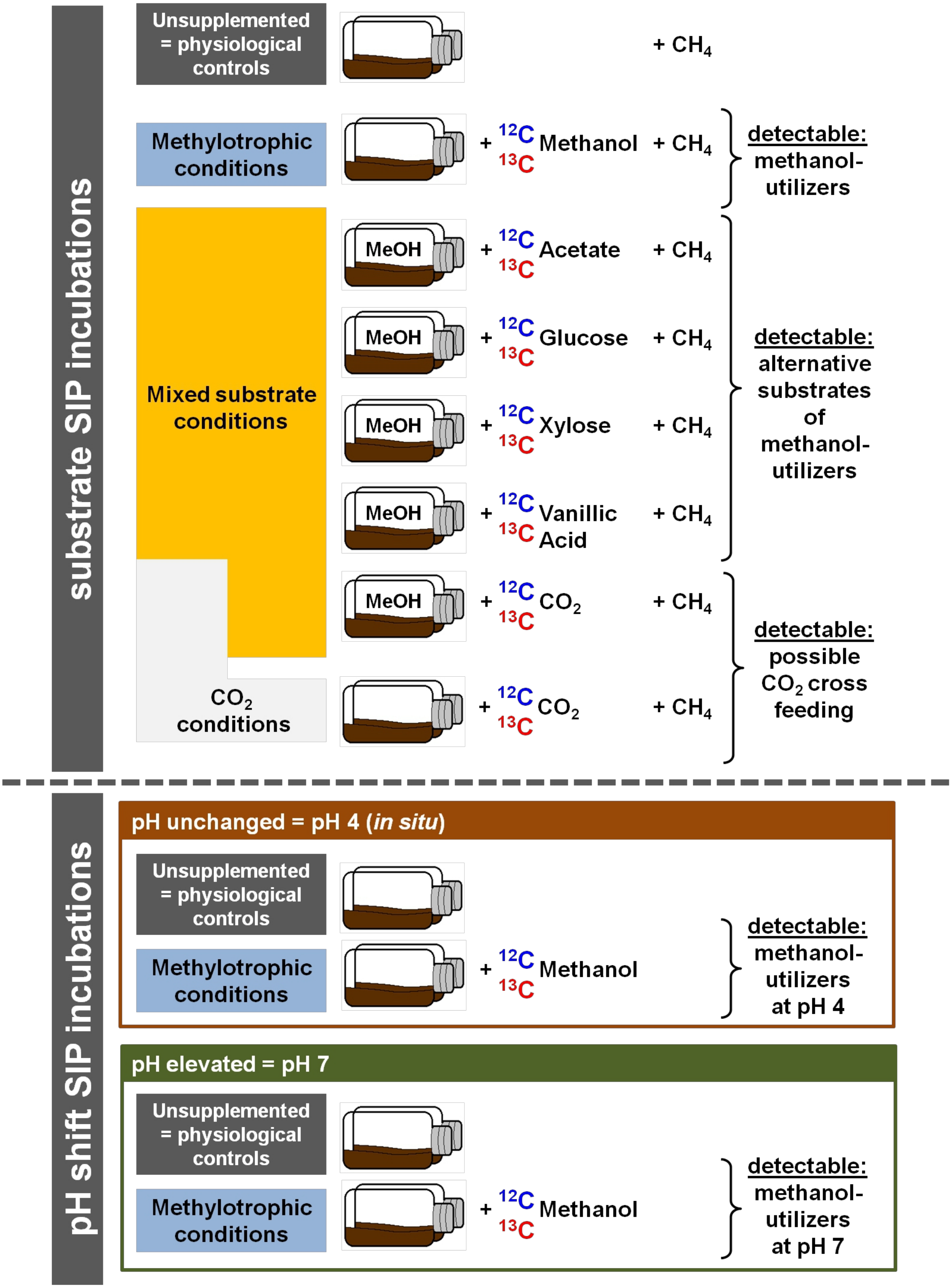

Figure S1. Overview on the experimental set-up of both SIP experiments. Substrate SIP and pH shift SIP experiment were conducted independent from each other with different soil samples as starting material. In the substrate SIP experiment several substrates (C_1 and C_n) were supplemented as ^{12}C- (blue font) or ^{13}C-isotopologue (red font). Additionally, [^{12}C]-methane (black font) was supplemented. In the pH shift SIP experiment solely [^{12}C]- or [^{13}C]-methanol was supplemented. For all pH 4 treatments (i.e. *in situ*) no adjustment of the soil slurry pH was conducted; for pH 7 treatments the initial soil slurry pH was elevated.

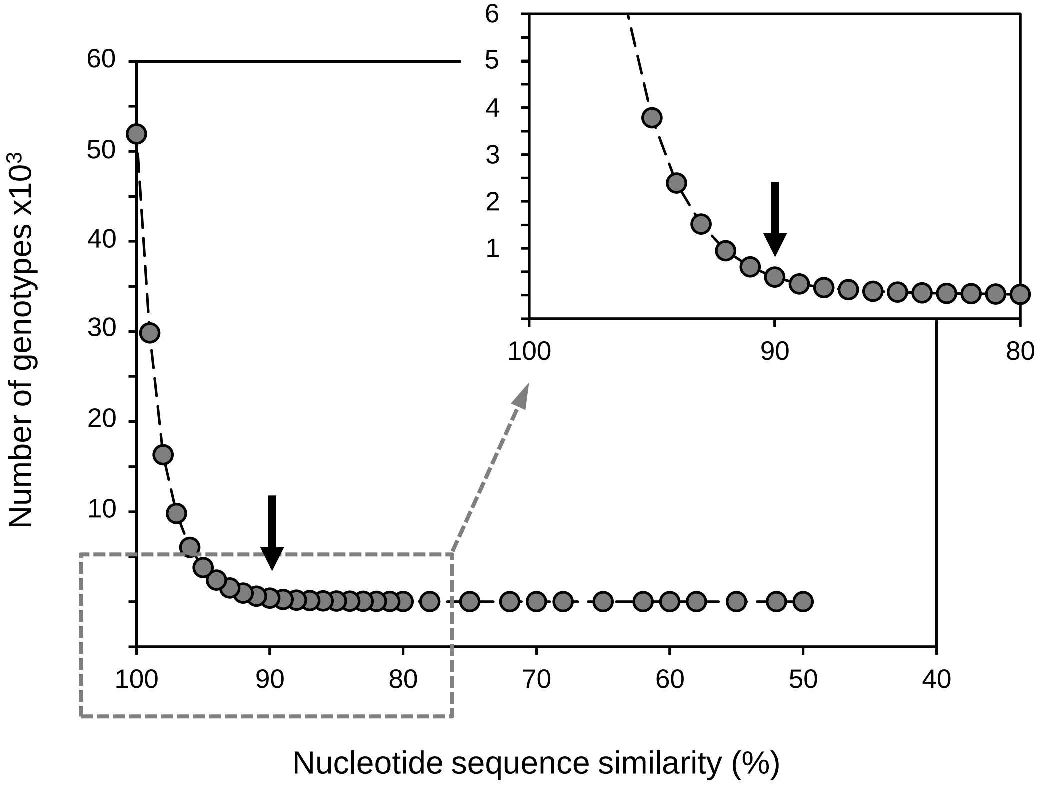

Figure S2. Correlation between the number of detected genotypes and the nucleotide sequence similarities of *mxaF* gene sequences. Shown is the result of the analysis of all detected *mxaF* gene sequences (i.e. 113 689 sequences) in all pyrosequencing amplicon libraries of both SIP experiments. Arrows indicate the similarity threshold value of 90% chosen for clustering and further analyses. Inset focuses on a sequence similarity range between 80% and 100%.

Table S1. Primer sequences of the 'conventional' primers used to amplify 16S rRNA gene, *mxaF* and ITS gene fragments in Step-1-PCR for amplicon pyrosequencing.

gene	primer[a]		sequence (5' – 3')[b]	amplicon	reference
16S rRNA	341f	(17 nt)	CCT ACG GG**N** GGC **W**GC AG	444 – 464 bp	Muyzer et al., 1993
	785/805 r	(21 nt)	GAC TAC **HV**G GGT ATC TAA TCC		Herlemann et al., 2011
mxaF	"mxaF1"				
	1003f	(21 nt)	GCG GCA CCA ACT GGG GCT CGT	552 bp	McDonald and Murrell 1997
	1555r	(21 nt)	CAT GAA **B**GG CTC CCA **R**TC CAT		Neufeld et al., 2007
	"mxaF2"				
	mxaF_for	(19 nt)	TGG AAC GAG ACC ATG CGT C	455 bp	Moosvi et al., 2005
	maxF_rev	(20 nt)	CAT GCA GAT GTG GTT GAT GC		Moosvi et al., 2005
pmoA	A189f	(18 nt)	GG**N** GAC TGG GAC TTC TGG	525 bp	Holmes et al., 1995
	A682r	(18 nt)	GAA **S**GC **N**GA GAA GAA **S**GC		Holmes et al., 1995
ITS	ITS1F	(22 nt)	CTT GGT CAT TTA GAG GAA GTA A	700 – 900 bp	Gardes and Bruns, 1993
	ITS4	(20 nt)	TCC TCC GCT TAT TGA TAT GC		White et al., 1990

[a] Primer notation according to the reference with primer length in brackets. 'f' or 'F' indicate the forward primers that are listed at first.
[b] Primer sequence with wobble bases in bold faces.

Table S2. Composition of the reagents for PCR reactions in the two-step approach PCR of barcoded amplification for bacterial gene fragments (16S rRNA gene, *mxaF, pmoA*).

Step-1-PCR (conventional primer pair)						Step-2-PCR (barcoded primer pair)		
Substrate SIP experiment			pH SIP experiment					
reagent (conc.)	Vol. [µl]	final conc.	reagent (conc.)	Vol. [µl]	final conc.	reagent (conc.)	Vol. [µl]	final conc.
5PRIME[a,b] (2.5 x)	8	1 x	KAPA2G Ready Mix[c] (2 x)	12.5	1 x	Buffer[a,d] (10x)	5	1 x
						dNTPs[e] (2 mM)	5	0.2 mM
						Taq Poly.[a] (5U/µl)	0.25	0.025 U
TaqMaster PCR Enhancer[a] (5 x)	1	1 x				TaqMaster PCR Enhancer[a] (5 x)	10	1 x
BSA[f] (3 %)	1.5	0.2 %	BSA[e] (3 %)	1.5	0.2 %			
Primer reverse (10 µM)	1	500 nM	Primer reverse (10 µM)	1.25	500 nM	Primer reverse (10 µM)	2,5	500 nM
Primer forward (10 µM)	1	500 nM	Primer forward (10 µM)	1.25	500 nM	Primer forward (5 µM)	5	500 nM
PCR-H_20[g]	4.5		PCR-H_20[g]	3.5		PCR-H_20[g]	17.25	
DNA template	3		DNA template	5		DNA template	5	
∑	20		∑	25		∑	50	

[a] Reagents from 5PRIME (Hamburg, Germany)
[b] Master mix (2.5 x) contains Taq DNA polymerase (62.5 U ml-1), 125 mM KCl, 75 mM Tris-HCl (pH 8.3), 4 mM Mg^{2+}, 0.5% Igepal®-CA630[+], 500 µM of each dNTP, stabilizers.
[c] Reagents from KAPABIOSYSTEM (Boston, MA, USA).
[d] Buffer (10 x) contains 500 mM KCl, 100 mM Tris-HCl pH 8.3 (at 25°C), 15 mM Mg^{2+}
[e] Equimolar mixture from 100 mM stock solutions from ROTH (Karlsruhe, Germany)
[f] Stock solution (3 mg ml^{-1}) from crystallised BSA (Bovine Serum Albumin) from Merck (Darmstadt, Germany).
[g] destilled Water (DNase/RNase free) from gibco (Paisley, United Kingdom)

Table S3. PCR programs to amplify 16S rRNA gene, *mxaF*, *pmoA* and ITS gene fragments in step-1-PCR and step-2-PCR of barcoded amplification.

Bacterial genes (16S rRNA gene, *mxaF, pmoA*)

	**Step-1-PCR** (conventional primer pair)						**Step-2-PCR** (barcoded primer pair)		
	Substrate SIP exp.			pH SIP experiment					
step	temp.	time		temp.	time		temp.	time	
initial denaturation	95°C	5 min		95°C	5 min		95°C	5 min	
amplification cycles[a]	95°C<br>55°C<br>72°C	60 sek<br>60 sek<br>60 sek	25 x [c]<br>30 x [d]<br>20 x [e]	95°C<br>55°C<br>72°C	30 sek<br>30 sek<br>60 sek	25 x [c]<br>30 x [e]	95°C<br>55°C<br>72°C	60 sek<br>60 sek<br>60 sek	15 x
terminal elongation	72°C	8 min		72°C	8 min		72°C	8 min	
storage	10°C	∞		10°C	∞		10°C	∞	

Fungal genes (ITS gene)

	**Step-1-PCR** (conventional primer pair or fusion primer pair)			**Step-2-PCR** (fusion primer pair)		
	both SIP experiments					
step	temp.	time				
initial denaturation	95°C	6 min		95°C	6 min	
amplification cycles[b]	94°C<br>60°C<br>(-1°C /cycle)<br>72°C	30 sek<br>30 sek<br>90 sek	10 x	94°C<br>50°C<br>72°C	45 sek<br>45 sek<br>60 sek	10 x
amplification cycles[a]	94°C<br>50°C<br>72°C	45 sek<br>45 sek<br>60 sek	20 x [f]<br>30 x [g]			
terminal elongation	72°C	10 min		72°C	10 min	
storage	10°C	∞		10°C	∞	

[a] Amplification cycles include denaturation, annealing and elongation in repeated cycles.
[b] Amplification cycles include denaturation, annealing and elongation in repeated cycles. PCR is a touchdown PCR with decreasing annealing temperatures by 1°C per cycle.
[c] Number of repeated cycles for 16S rRNA gene fragment amplification.
[d] Number of repeated cycles for *mxaF* gene fragment amplification with primer pair "mxaF1" and for *pmoA* gene fragment.
[e] Number of repeated cycles for *mxaF* gene fragment amplification with primer pair "mxaF2".
[f] Number of repeated cycles for step-1-PCR with the conventional primer pair (subsequently step-2-PCR conducted).
[g] Number of repeated cycles for step-1-PCR with the fusion primer pair (no step-2-PCR conducted).

Table S4. Structure of the fusion primers used for amplicon pyrosequencing.

	fusion primer structure (5' – 3')			
	**adaptor sequence[a]**	**key**	**barcode[b]**	**gene specific primer[c]**
forward fusion primer				
	CCATCTCATCCCTGCGTGTCTCCGAC	TCAG	NNNNNNN (6 or 10 nt)	'conventional' primer
reverse fusion primer				
	CCTATCCCCTGTGTGCCTTGGCAGTC	TCAG		'conventional' primer

[a] Adaptor sequences are provided by Roche (Mannheim, Germany). Forward fusion primer contains adaptor sequence A, reverse fusion primer contains adaptor sequence B.
[b] Barcode sequences are given in Table S6 for bacterial genes and S8 for fungal genes and were used to create amplicon libraries and assign sequences to different samples.
[c] Primer sequences of gene specific primers are given in Table S1. Forward fusion primer were constructed with the 'conventional' forward primer (bacterial genes) and the 'conventional' reverse primer (fungal bacterial genes), respectively. Reverse fusion primer were constructed with the 'conventional' reverse primer (bacterial genes) and the 'conventional' forward primer (fungal bacterial genes), respectively.

Table S5. Composition of the reagents for PCR reactions in the two-step approach PCR of barcoded amplification for fungal gene fragments (ITS).

Step-1-PCR (conventional primer pair or fusion primer pair)						Step-2-PCR (fusion primer pair)		
Substrate SIP experiment			pH SIP experiment					
reagent (conc.)	Vol. [µl]	final conc.	reagent (conc.)	Vol. [µl]	final conc.	reagent (conc.)	Vol. [µl]	final conc.
GoTaq Green Mastermix (2 x)[a]	25	1 x	KAPA2G Ready Mix[b] (2 x)	12.5	1 x	GoTaq Green Mastermix (2 x)[a]	25	1 x
			BSA[c] (3 %)	1.5	0.2 %			
Primer reverse (10 µM)[d]	1	200 nM	Primer reverse (10 µM)	1.25	500 nM	Primer reverse (10 µM)	1	200 nM
Primer forward (10 µM)[d]	1	200 nM	Primer forward (10 µM)	1.25	500 nM	Primer forward (10 µM)	1	200 nM
PCR-H_20[a]	to 50		PCR-H_20[e]	3.5		PCR-H_20a	18	
DNA template[f]	1 to 10		DNA template	5		DNA template	5	
∑	50		∑	25		∑	50	

[a] Reagents from Promega (Madison, WI, USA)
[b] Reagents from KAPABIOSYSTEM (Boston, MA, USA).
[c] Stock solution (3 mg ml^{-1}) from crystallised BSA (Bovine Serum Albumin) from Merck (Darmstadt, Germany).
[d] The conventional primer pair ITS1F and ITS4 or the fusion primer pair of these primers were used. Fusion primers possess at the 5'-end of the primer sequence an additional adaptor and barcode sequences (see Table S4)
[e] Destilled Water (DNase/RNase free) from gibco (Paisley, United Kingdom)
[f] DNA template was added in different volumes ranging from 1 to 10 µl dependent of PCR result.

Table S6. Sequences of the barcodes used to identify individual samples in the pyrosequencing amplicon libraries for 16S rRNA gene and mxaF sequences of both SIP experiments.

	treatm.[a]	lib[b]	Pool[c]	barcode (5'-3') initial (6 nt)	barcode (5'-3') modified[d] (5 nt)
Substrate SIP experiment	$t0_1$	H	1	TACTAG	ACTAG [e]
		M	1	TACGTG	ACGTG [e]
		L	1	ATAGTG	TAGTG [e]
	$t0_2$	H	2	TACTAG	TACTA [f]
		M	2	TACGTG	TACGT [f]
		L	2	ATAGTG	ATAGT [f]
	$t0_3$	H	2	TATCAG	TATCA [f]
		M	2	TATATG	TATAT [f]
		L	2	TAGCTG	TAGCT [f]
	$t0_4$	H	2	AGTATG	AGTAT [f]
		M	2	AGCACG	ACACG [h]
		L	2	ACACTG	ACACT [f]
	^{12}C Methanol	H	1	TATCAG	ATCAG [e]
		M	1	TATATG	ATATG [e]
		L	1	TAGCTG	AGCTG [e]
	^{13}C Methanol	H	1	AGTATG	GTATG [e]
		M	1	AGCACG	GCACG [e]
		L	1	ACACTG	CACTG [e]
	^{12}C Acetate +	H	2	TAGTGC	TGTGC [h]
		M	2	TAGCAC	TAGCA [f]
		L	2	TACAGC	TACAG [f]
	^{13}C Acetate +	H	2	ACTCGC	ACTCG [f]
		M	2	ACTATC	ACTAT [f]
		L	2	ACGAGC	ACGAG [f]
	^{12}C Glucose +	H	1	TAGTGC	AGTGC [e]
		M	1	TAGCAC	AGCAC [h]
		L	1	TACAGC	ACAGC [e]
	^{13}C Glucose +	H	1	ACTCGC	CTCGC [e]
		M	1	ACTATC	CTATC [e]
		L	1	ACGAGC	CGAGC [e]
	^{12}C Xylose +	H	2	TCTCAC	TCTCA [f]
		M	2	TCTAGC	TCTAG [f]
		L	2	TATCGC	TATCG [f]
	^{13}C Xylose +	H	2	AGTCAC	AGTCA [f]
		M	2	AGCTAC	AGCTA [f]
		L	2	AGCGTC	AGCGT [f]

	treatm.[a]	lib[b]	Pool[c]	barcode (5'-3') initial (6 nt)	barcode (5'-3') modified[d] (5 nt)
Substrate SIP experiment	^{12}C Vanillic Acid +	H	2	TGTGAC	TGTGA [f]
		M	2	TGTCTC	TGTCT [f]
		L	2	TGCATC	TGCAT [f]
	^{13}C Vanillic Acid +	H	2	ATCTGC	ATCTG [f]
		M	2	ATCATC	ATCAT [f]
		L	2	ATACTC	ATACT [f]
	^{12}C CO_2 +	H	1	TCTCAC	CTCAC [e]
		M	1	TCTAGC	CTAGC [e]
		L	1	TATCGC	ATCGC [e]
	^{13}C CO_2 +	H	1	AGTCAC	GTCAC [e]
		M	1	AGCTAC	GCTAC [e]
		L	1	AGCGTC	GCGTC [e]
	^{12}C CO_2	H	1	TGTGAC	GTGAC [e]
		M	1	TGTCTC	GTCTC [e]
		L	1	TGCATC	GCATC [e]
	^{13}C CO_2	H	1	ATCTGC	TCTGC [e]
		M	1	ATCATC	TCATC [e]
		L	1	ATACTC	TACTC [e]
pH SIP experiment	t0 pH 4	H	3	ACTATC	ACATC [g]
		M	3	TAGTGC	TATGC [g]
		L	3	TAGCAC	TACAC [g]
	^{12}C Methanol pH 4	H	3	ATATAC	ATTAC [g]
		M	3	ATCATC	ATATC [g]
		L	3	ATACTC	ATCTC [g]
	^{13}C Methanol pH 4	H	3	AGCTAC	AGTAC [g]
		M	3	AGCGTC	AGGTC [g]
		L	3	AGCACG	AGACG [g]
	t0 pH 7	H	3	ATAGTG	ATGTG [g]
		M	3	TCTAGC	TCAGC [g]
		L	3	TATCGC	TACGC [g]
	^{12}C Methanol pH 7	H	3	TGTGAC	TGGAC [g]
		M	3	TGTCTC	TGCTC [g]
		L	3	TGCATC	TGATC [g]
	^{13}C Methanol pH 7	H	3	TACAGC	TAAGC [g]
		M	3	TACTAG	TATAG [g]
		L	3	TACGTG	TCGTG [h]

[a] Abbreviation for ‘treatment’; indicates supplemented substrate. ‘^{12}C’ indicates [^{12}C]-substrate; ‘^{13}C’ indicates [$^{13}C_u$]-substrate. A cross indicates additionally supplemented [^{12}C]-methanol in Substrate SIP experiment treatments
[b] Abbreviation for ‘library’; indicates pyrosequencing amplicon library. ‘H’, library of heavy fraction; ‘M’, library of middle fraction; ‘L’, library of light fraction.
[c] Amplicons were pooled for pyrosequencing to minimize number of barcodes required. Number 1 to 3 indicates different ‘amplicon sequence pools’.
[d] Barcode sequence after manually modification.
[e] Modification: First base of initial barcode was removed to create a unique barcode.
[f] Modification: Last base of initial barcode was removed to create a unique barcode.
[g] Modification: Third base of initial barcode was removed to create a unique barcode.
[h] Modification: Second base of initial barcode was removed to create a unique barcode.

Table S7. Number of all sequences obtained from the pyrosequencing amplicon libraries of both SIP experiments. The dash indicates no sample / no data / no amplicon library.

	treatm.[a]	lib[b]	16S rRNA gene raw	16S rRNA gene filtered[c]	*mxaF* raw	*mxaF* filtered[c]	ITS gene raw	ITS gene filtered[c]
	**total**		**200785**	**105689**	**139329**	**113689**	**237495**	**95065**
Substrate SIP experiment	$t0_1$	**ALL**	**2939**	**1492**	**3479**	**2521**	**11282**	**4303**
		**H**	1225	760	1223	898	3315	1477
		**M**	22	10	1049	773	4093	1440
		**L**	1692	722	1207	850	3874	1386
	$t0_2$	**ALL**	**5014**	**2882**	**6423**	**5756**	**11298**	**4316**
		**H**	495	292	2605	2302	3391	1489
		**M**	2152	1133	1890	1734	4097	1456
		**L**	2367	1457	1928	1720	3810	1371
	$t0_3$	**ALL**	**6763**	**4376**	**2277**	**2040**	-	-
		**H**	2277	1506	1025	945	-	-
		**M**	2323	1496	710	621	-	-
		**L**	2163	1374	542	474	-	-
	$t0_4$	**ALL**	**5354**	**3283**	**3741**	**3285**	-	-
		**H**	260	185	2197	1995	-	-
		**M**	2650	1615	752	600	-	-
		**L**	2444	1483	792	690	-	-
	^{12}C Methanol	**ALL**	**4270**	**2103**	**1513**	**1081**	**10970**	**4263**
		**H**	1060	528	719	551	3694	1466
		**M**	1117	586	694	474	3632	1437
		**L**	2093	989	100	56	3644	1360
	^{13}C Methanol	**ALL**	**4770**	**2328**	**1495**	**955**	**11043**	**4272**
		**H**	1134	571	694	497	3122	1460
		**M**	1810	868	566	328	3980	1435
		**L**	1826	889	235	130	3941	1377
	^{12}C Acetate +	**ALL**	**7034**	**4454**	**3666**	**3220**	**13117**	**4348**
		**H**	3061	2128	1543	1384	3750	1474
		**M**	1685	960	865	717	4827	1449
		**L**	2288	1366	1258	1119	4540	1425
	^{13}C Acetate +	**ALL**	**9039**	**5666**	**4291**	**3743**	**11431**	**4322**
		**H**	3854	2536	1743	1484	3552	1397
		**M**	3028	1902	1173	1067	3955	1499
		**L**	2157	1228	1375	1192	3924	1426

	treatm.[a]	lib[b]	16S rRNA gene raw	16S rRNA gene modified[c]	*mxaF* raw	*mxaF* modified[c]	ITS gene raw	ITS gene modified[c]
Substrate SIP experiment	^{12}C Glucose +	ALL	**5281**	**2536**	**3513**	**2767**	**13211**	**4427**
		H	1820	903	1136	973	3776	1469
		M	1272	543	1261	994	5154	1497
		L	2189	1090	1116	800	4281	1461
	^{13}C Glucose +	ALL	**5306**	**2217**	**2525**	**1837**	**13270**	**4406**
		H	1961	661	397	266	4616	1481
		M	2407	1097	1197	930	4105	1477
		L	938	459	931	641	4549	1448
	^{12}C Xylose +	ALL	**7588**	**4667**	**3300**	**2923**	**12712**	**4420**
		H	2823	1864	1045	945	3984	1457
		M	2203	1415	782	710	4902	1489
		L	2562	1388	1473	1268	3826	1474
	^{13}C Xylose +	ALL	**8039**	**4525**	**5194**	**4645**	**12067**	**4440**
		H	3352	2004	2967	2676	3977	1491
		M	2206	1117	561	469	4201	1501
		L	2481	1404	1666	1500	3889	1448
	^{12}C Vanillic Acid +	ALL	**9161**	**5476**	**3248**	**2871**	**12947**	**4246**
		H	3677	2498	1296	1125	2706	1467
		M	2952	1414	1017	913	6294	1424
		L	2532	1564	935	833	3947	1355
	^{13}C Vanillic Acid +	ALL	**7890**	**4675**	**2910**	**2501**	**11384**	**4270**
		H	2783	2035	1061	852	3499	1428
		M	2638	1327	893	805	3852	1444
		L	2469	1313	956	844	4033	1398
	^{12}C CO2 +	ALL	**4311**	**2094**	**2835**	**2103**	**11783**	**4340**
		H	827	522	1180	935	2696	1493
		M	1454	731	774	501	5393	1471
		L	2030	841	881	667	3694	1376
	^{13}C CO2 +	ALL	**4453**	**2120**	**3830**	**2759**	**12435**	**4307**
		H	705	393	1558	1166	3329	1454
		M	1886	923	1033	707	4691	1452
		L	1862	804	1239	886	4415	1401
	^{12}C CO2	ALL	**5642**	**2338**	**2390**	**1780**	**12351**	**4258**
		H	1818	795	1008	759	3808	1413
		M	2008	754	662	483	4365	1436
		L	1816	789	720	538	4178	1409

	treatm.[a]	lib[b]	16S rRNA gene raw	16S rRNA gene modified[c]	*mxaF* raw	*mxaF* modified[c]	ITS gene raw	ITS gene modified[c]
Sub. SIP exp	^{13}C CO_2	**ALL**	**5941**	**2704**	**1661**	**1224**	**16841**	**4268**
		**H**	2347	1122	703	526	3534	1488
		**M**	1877	884	342	239	8430	1390
		**L**	1717	698	616	459	4877	1390
pH SIP experiment	t0 pH 4	**ALL**	**9096**	**3917**	**10014**	**8364**	**5940**	**4301**
		**H**	2852	1243	2797	2443	1767	1440
		**M**	3217	1397	3435	2880	2030	1441
		**L**	3027	1277	3782	3041	2143	1420
	^{12}C Methanol pH 4	**ALL**	**19781**	**12097**	**10795**	**9110**	**6340**	**4326**
		**H**	4334	2587	2314	1928	2329	1422
		**M**	4866	3107	3533	3003	1986	1455
		**L**	10581	6403	4948	4179	2025	1449
	^{13}C Methanol pH 4	**ALL**	**23530**	**13979**	**10348**	**7716**	**6383**	**4286**
		**H**	6775	3737	1698	1306	2324	1429
		**M**	8454	4757	3588	2949	1654	1434
		**L**	8301	5485	5062	3461	2405	1423
	t0 pH 7	**ALL**	**10662**	**4460**	**13726**	**11167**	**6430**	**4251**
		**H**	3117	1310	5407	4360	2114	1408
		**M**	3281	1480	2860	2370	2269	1416
		**L**	4264	1670	5459	4437	2047	1427
	^{12}C Methanol pH 7	**ALL**	**13412**	**4968**	**17830**	**14387**	**6947**	**4348**
		**H**	4556	1741	5734	4591	2525	1459
		**M**	5189	1701	4889	4008	2153	1451
		**L**	3667	1526	7207	5788	2269	1438
	^{13}C Methanol pH 7	**ALL**	**15509**	**6332**	**18325**	**14934**	**7313**	**4347**
		**H**	6848	2980	8171	6776	2551	1462
		**M**	4277	1744	7173	5811	2288	1438
		**L**	4384	1608	2981	2347	2474	1447

[a] Abbreviation for 'treatment'; indicates supplemented substrates. '^{12}C' indicates [^{12}C]-substrate; '^{13}C' indicates [$^{13}C_u$]-substrate. A cross indicates additionally supplemented [^{12}C]-methanol in Substrate SIP experiment.

[b] Abbreviation for 'library'; indicates pyrosequencing amplicon library. 'ALL', library of combined data set of 'H', 'M' and 'L'; 'H', library of heavy fraction; 'M', library of middle fraction; 'L', library of light fraction.

[c] Number of all remained sequences after quality and chimera check, clustering with specific cut-off values and detection of the correct forward primer sequence.

Table S8. Sequences of the barcodes used to identify individual samples in the pyrosequencing amplicon libraries for ITS gene fragments.

	treatm.[a]	lib[b]	10 nt barcode[c] (5'-3')	treatm.[a]	lib[b]	10 nt barcode[c] (5'-3')
Substrate SIP	$t0_1$	H	CTCGCGTGTC	^{12}C Xylose +	H	AGCACTGTAG
		M	CGTCTAGTAC		M	CATAGTAGTG
		L	TCGTCGCTCG		L	CGTAGACTAG
	$t0_2$	H	CACGCTACGT	^{13}C Xylose +	H	AGACTATACT
		M	TCTAGCGACT		M	TACAGATCGT
		L	AGTATACATA		L	ACAGTATATA
	^{12}C Methanol	H	ACGAGTGCGT	^{12}C Vanillic Acid +	H	AGACGCACTC
		M	TAGTATCAGC		M	TACTGAGCTA
		L	TCTACGTAGC		L	ACGACTACAG
	^{13}C Methanol	H	ACATACGCGT	^{13}C Vanillic Acid +	H	ACTGTACAGT
		M	CAGTAGACGT		M	TACACGTGAT
		L	TCTATACTAT		L	TGTGAGTAGT
	^{12}C Acetate +	H	ACGCTCGACA	^{12}C CO_2 +	H	CGTGTCTCTA
		M	TGATACGTCT		M	TCACGTACTA
		L	TGTACTACTC		L	TAGAGACGAG
	^{13}C Acetate +	H	ACGCGAGTAT	^{13}C CO_2 +	H	ATAGAGTACT
		M	TGAGTCAGTA		M	TCGCACTAGT
		L	TGACGTATGT		L	AGCTCACGTA
	^{12}C Glucose +	H	ATCAGACACG	^{12}C CO_2 +	H	ATATCGCGAG
		M	CGAGAGATAC		M	ATACGACGTA
		L	TACGAGTATG		L	TACTCTCGTG
	^{13}C Glucose +	H	AGCGTCGTCT	^{13}C CO_2 +	H	AGTACGCTAT
		M	TACGCTGTCT		M	TCGATCACGT
		L	ACGCGATCGA		L	ACTAGCAGTA
pH SIP	t0 pH 4	H	TCGATCACGT	t0 pH 7	H	TCTATACTAT
		M	ACAGTATATA		M	AGCTCACGTA
		L	CGATCGTATA		L	CGTACAGTCA
	^{12}C Methanol pH 4	H	TCGCACTAGT	^{12}C Methanol pH 7	H	TGACGTATGT
		M	ACGCGATCGA		M	AGTATACATA
		L	CGCAGTACGA		L	CGTACTCAGA
	^{13}C Methanol pH 4	H	TCTAGCGACT	^{13}C Methanol pH 7	H	TGTGAGTAGT
		M	ACTAGCAGTA		M	AGTCGAGAGA
		L	CGCGTATACA		L	CTATAGCGTA

[a] Abbreviation for 'treatment'; indicates supplemented substrate. '^{12}C' indicates [^{12}C]-substrate; '^{13}C' indicates [$^{13}C_u$]-substrate. A cross indicates additionally supplemented [^{12}C]-methanol in substrate SIP treatments

[b] Abbreviation for 'library'; indicates pyrosequencing amplicon library. 'H', library of heavy fraction; 'M', library of middle fraction; 'L', library of light fraction.

[c] 10 nt barcodes are internal provided by Roche (Roche Applied Science).

Table S9. Composition of the reagents for PCR reactions of the qPCR standard preparation, cloning and qPCR assays.

qPCR standard preparation and cloning

reagent (conc.)	**16S rRNA gene / *mxaF*** Vol. [µl]	final conc.	***mmoX*** Vol. [µl]	final conc.	**M13[a] / pJET[b]** Vol. [µl]	final conc.
Buffer[c] (10x)	5	1 x	5	1 x	5	1 x
dNTPs[d] (2 mM)	5	200 µM	5	200 µM	5	200 µM
$MgCl_2$[c] (25 mM)	3	1.5 mM	6	3 mM	3	1.5 mM
Taq Polym.[c] (5 U/µl)	0.25	0.025 U	0.25	0.025 U	0.25	0.025 U
Primer forward (10 µM)	2.5	500 nM	2.5	500 nM	1	200 nM
Primer reverse (10 µM)	2.5	500 nM	2.5	500 nM	1	200 nM
PCR-H_20[e]	26.75		23.75		29.75	
DNA-template[f]	5		5		5	
∑	50		50		50	

qPCR assays

reagent (conc.)	**16S rRNA gene** Vol. [µl]	final conc.	***mxaF / mmoX*** Vol. [µl]	final conc.	**Inhibit[g]** Vol. [µl]	final conc.
SensiFast SYBR[h] (2x)	10	1x	10	1x	10	1x
Primer forward (10 µM)	0.5	250 nM	1	500 nM	1	500 nM
Primer reverse (10 µM)	0.5	250 nM	1	500 nM	1	500 nM
BSA (3 %)[i]			1	0.15 %		
PCR - H_20[e]	4		2		3	
DNA-template[j]	5		5		5	
∑	20		20		20	

[a] Reaction mixture for preparing artificial DNA using primer pair “M13_for/M13_rev”
[b] Reaction mixture used to amplify clons with correct insert to prepare qPCR standards
[c] Reagents from BILATEC AG, Viernheim, Germany.
[d] Equimolar mixture from 100 mM stock solutions from ROTH (Karlsruhe, Germany)
[e] Destilled Water (DNase/RNase free) from gibco (Paisley, United Kingdom)
[f] DNA templates were genomic DNA (16S rRNA, *mxaF*, *mmoX*), circular plasmid pCR 2.1-TOPO from Invitrogen (“M13”) or aqueous clone suspension (“pJET”)
[g] Primer pair used in qPCR reaction was “M13_rev / T7-Prom”
[h] Reagents from SensiMix™ SYBR® & Fluorescein Kit (Bioline GmbH, Luckenwalde, Germany)
[i] Stocksolution (3 mg ml^{-1}) from crystallised BSA (Bovine Serum Albumin) from Merck (Darmstadt, Germany).
[j] Diluted DNA templates (1:100 or 1:100)

Table S10. Primer sequences of the primers used for qPCR and to prepare the qPCR standards.

gene	primer[a]		sequence (5' – 3')[b]	amplicon	reference
16S rRNA	341f	(17 nt)	CCT ACG GG**N** GGC **W**GC AG	193 bp	Muyzer et al., 1993
	534r	(17 nt)	ATT ACC GCG GCT GCT GG		Muyzer et al., 1993
mxaF	mxaF_for	(19 nt)	TGG AAC GAG ACC ATG CGT C	455 bp	Moosvi et al., 2005
	maxF_rev	(20 nt)	CAT GCA GAT GTG GTT GAT GC		Moosvi et al., 2005
mmoX	mmoXAf	(17 nt)	ACC AAG GA**R** CA**R** TTC AA	295 bp	Aumann et al., 2000
	McеI422r	(18 nt)	GAA GCC GCA TTG ATG GGT		Kolb et al., 2005
vector pCR 2.1	T7-Prom_f	(20 nt)	TAA TAC GAC TCA CTA TAG GG	178 bp	
	M13_rev	(18 nt)	CAG GAA ACA GCT ATG ACC		
	M13_for	(16 nt)	GTA AAA CGA CGG CCA G	199 bp[c]	
vector pJET 1.2	pJET_for	(23 nt)	CGA CTC ACT ATA GGG AGA GCG GC	variable[d]	ThermoScientific, 2011[e]
	pJET_rev	(24 nt)	AAG AAC ATC GAT TTT CCA TGG CAG		ThermoScientific, 2011[e]

[a] Primer notation according to the reference with primer length in brackets. 'f' or 'for' indicate the forward primers that are listed at first.
[b] Primer sequence with wobble bases in bold faces.
[c] Amplicon length in combination with "M13_rev"
[d] Amplicon length depends on ligated vector insert (i.e. ligated insert + 119 bp).
[e] Primer sequences available in product information for "ThermoScientific CloneJET PCR Cloning Kit"

Table S11. PCR programs to amplify gene fragments for qPCR standards.

step	temperature	time	
initial denaturation	95°C	5[b] / 8[c] min	
amplification cycles[a]	95°C<br>55°C<br>72°C	60[b] / 30[c] sek<br>60[b] / 30[c] sek<br>60 sek	30 x
terminal elongation	72°C	8 min	
storage	10°C	∞	

[a] Amplification cycles include denaturation, annealing and elongation in repeated cycles.
[b] Time applied in the PCR program for preparing gene fragments of 16S rRNA gene, *mxaF* and *mmoX* from pure cultures using specific primers as well as artificial DNA using primer pair "M13_for/M13_rev"
[c] Time applied in the PCR program for the amplification of inserts in vector pCR2.1 to prepare qPCR standards.

Table S12. Modified qPCR programs to amplify 16S rRNA gene, *mxaF*, *mmoX* and artificial DNA (Inhibit).

step	16S rRNA gene temp.	16S rRNA gene time	*mxaF* temp.	*mxaF* time	*mmoX* temp.	*mmoX* time	Inhibit temp.	Inhibit time
initial denaturation	95°C	8 min	95°C	8 min	95°C	8 min	95°C	8 min
amplification. cycles[a] 40x	3-step<br>95°C<br>55.7°C<br>**72°C**	 30 sek<br>25 sek<br>25 sek	4-step<br>95°C<br>64°C<br>72°C<br>**78°C**	 30 sek<br>20 sek<br>30 sek<br>12 sek	4-step<br>95°C<br>61°C<br>72°C<br>**78°C**	 30 sek<br>30 sek<br>30 sek<br>12 sek	3-step<br>95°C<br>61.2°C<br>**72°C**	 30 sek<br>15 sek<br>15 sek
terminal elongation	72°C	10 min	72°C	10 min	72°C	10 min	72°C	10 min
melting curve[b] 47x	72°C<br>↓<br>(+0.5°C /cycle)<br>↓<br>95°C	12 sek	72°C<br>↓<br>(+0.5°C /cycle)<br>↓<br>95°C	12 sek	72°C<br>↓<br>(+0.5°C /cycle)<br>↓<br>95°C	12 sek	72°C<br>↓<br>(+0.5°C /cycle)<br>↓<br>95°C	12 sek
storage	10°C	∞	10°C	∞	10°C	∞	10°C	∞
according to[c]	Zaprasis *et al.*, 2010				“MCEL“ Kolb *et al.*, 2005		“INHIB-CORR” Degelmann *et al.*, 2010	

[a] Amplification cycles include denaturation, annealing and elongation in repeated cycles. Bold faces indicate the step when fluorescence signal was recorded, i.e. in a 3-step protocol the last 12 seconds of elongation step and in a 4-step protocol an extra step with higher temperatures than elongation to melt small unspecific PCR products and thus reduce bias of unspecific fluorescence signals.

[b] Melting curve analysed was performed from 72°C to 95°C with increments of 0.5°C per cycle.

[c] Established qPCR assays were slightly modified in this study, the original qPCR assay references are quoted.
