# Supplementary material for: Acidotolerant Bacteria and Fungi as a Sink of Methanol-Derived Carbon in a Deciduous Forest Soil
Source: Front Microbiol. 2017 Jul 24;8:1361. doi: 10.3389/fmicb.2017.01361 (PMC5523551; doi:10.3389/fmicb.2017.01361)
Supplement: Supplementary file 2 [file Presentation_2.pdf]

# Supplementary Information on Results

## Conversion of methanol and multi-carbon substrates in the Substrate SIP experiment

Methanol was supplemented at low concentrations (1mM daily) resulting in a gradually formation of $CO_2$ evidently after five days of treatments indicating a utilization of methanol (Figure S4A). Consumption of additionally supplemented methane (200 p.p.m.) was not observed in any treatment (Figure S4B and E) suggesting no stimulation of high affinity methanotrophs. The capacity of active methylotrophs to utilize multi-carbon compounds such as the common intermediate of anaerobic organic matter degradation acetate ($C_2$) or plant-derived compounds such as xylose ($C_5$), glucose ($C_6$) and vanillic acid ($C_8$) was analysed. The disappearance of supplemented substrates correlated with the formation of $CO_2$ indicating microbial utilization of substrates (Figure S4E and F). The amount of $CO_2$ detectable in [$^{12}$C]- and [$^{13}$C]-treatments was similar suggesting no preferential utilization of [$^{12}$C]-substrates. In general, increase of [$^{13}$C]-$CO_2$ was linear for methanol treatments and exponential for multi-carbon substrate treatments (Figure S4A and F). The corresponding carbon recovery was about 20% for methanol treatments and ranged from 5% (first pulse) to 22% (last pulse) for multi-carbon substrate treatments.

## Effect of pH on methanol utilization in the pH shift SIP experiment

The pH did not change in both treatments, i.e. 'pH 4' with *in situ* pH and 'pH 7' with pH adjusted to neutral. $CO_2$ formation was higher at neutral pH in both, control and methanol treatments (Figure S4C and D). Similar amounts of $CO_2$ were detected in [$^{12}$C]- and [$^{13}$C]-treatments suggesting no preferential utilization of [$^{12}$C]-methanol. In accordance with [$^{13}$C]-methanol treatments of the Substrate SIP experiment, a continuous formation of [$^{13}$C]-$CO_2$ per [$^{13}$C]-methanol pulse was observed (Figure S4C and D) with carbon recoveries of 14% (pH4) and 30% (pH7).

## Microbial community shaping effect of multi-carbon substrates and pH

Between [$^{12}$C]-, [$^{13}$C]- and combined dataset derived sequences only minor differences were noticeable observed employing in a nMDS analysis (Figure S5). The mean coverage was 98.8±0.66% for 16S rRNA (Figure S6A), 99.08±0.64% for *mxaF* (Figure S7A), and 98.44±0.29% for ITS (Figure S8A). The amount of detected genotypes was for bacterial and fungal phylotypes >100 (Figure S6B and S8B) and for mxaF genotypes >50 (Figure S7B. Chao 1 indices indicated higher numbers (Figure S6F, S7F and S8F) and almost no domination of single taxa were observed (Figure S6C,D,E; S7C,D,E and S8C,D,E) indicative for a diverse microbial community at $t_0$ and the later treatments. Treatments affected the microbial community composition significantly whereas pH had a higher influence on the bacterial community and multi-carbon substrates (i.e. sugars and acetate) showed higher influence on fungal community (Figure S5; Table S13 for 16S, S16 for *mxaF*, S15 for ITS).

Bacterial communities with *in situ* pH ($t_0$ and treatments) were dominated by *Actinobacteria, Planctomycetes* and *Proteobacteria*, with domination of *Gammaproteobacteria* over *Alphaproteobaceteria* (abundant in methanol treatments) and *Betaproteobacteria* (abundant in sugar and vanillic acid treatments), and neutral pH communities were dominated by *Bacteroidetes* (Figure S3A, Table S17). Focussing on methylotrophs, *Methylobacterium*-related genotypes decreased and *Hyphomicrobium*-related genotypes increase in *in situ* pH

treatments and at neutral pH the initial low abundant *Methylobacterium*-related genotypes increased (Figure S9, Table S18). Abundance of *Ascomycota* and *Basidiomycota* was balanced in fungal communities with a domination of *Basidiomycota* in acetate and sugar treatments mainly contributed by *Trichosporon*-related genotypes (Figure S3B, Table S12).

## Identification of methanol-utilizing bacteria and fungi in the Forest soil – occurence of *Corynebacterium* and *Rhodanobacter* phylotypes

Analysing all fractions of Substrate SIP experiments revealed the predominance of a *Corynebacterium*-related phylotype ($OTU_{16S6}48$; sequence identity 93% (Table S2) in acetate, xylose, vanillic acid and $CO_2$+methanol treatments (Figure S10). Fractions of the treatment pH4 of the pH shift SIP experiment were dominated by a *Rhodanobacter*-related phylotype (($OTU_{16S}300$; sequence identity 99% (Table S2). Both phylotypes were highly abundant in both [$^{12}C$]- and [$^{13}C$]-fractions and thus a labelling was not likely. We considered them as artefactual and excluded them from further analyses.

## List of supplementary Figures

**Figure S1.** nMDS analyses of bacterial and fungal communities in the ‘heavy’ and ‘middle’ fractions of both SIP experiments.

**Figure S2.** Gene numbers of mmoX genes of treatments with different pH in the pH shift SIP experiment.

**Figure S3.** Bacterial (A) and fungal (B) phyla composition after different substrate or pH treatments.

**Figure S4.** $CO_2$ formation and conversion of the different multi-carbon substrates in the soil slurry treatments

**Figure S5.** nMDS analyses of the bacterial community (A), the fungal community (B) and *mxaF* sequences (C) from different substrate or pH treatments.

**Figure S6.** Diversity and richness estimators of 16S rRNA gene sequences from pyrosequencing amplicon pools at similarity level 90.1% (family level).

**Figure S7.** Diversity and richness estimators of *mxaF* sequences from pyrosequencing amplicon pools at similarity level of 90%.

**Figure S8.** Diversity and richness estimators of ITS gene sequences from pyrosequencing amplicon pools at similaritiy level of 97% (species level).

**Figure S9.** Composition of the various *mxaF* phylotypes after different substrate or pH treatments.

**Figure S10.** Bacterial phyla composition in the ‘heavy’ fractions after different substrate or pH treatments based on all detected phylotypes.

## List of supplementary Tables

**Table S1.** Relative abundances of labeled bacterial taxa (OTU) based on 16S rRNA gene sequences in all fractions (H, heavy; M, middle; L, light) of [$^{12}$C]- and [$^{13}C_1$]-methanol treatment of Substrate SIP experiment.

**Table S2.** Taxonomic affiliation of bacterial phylotypes (OTUs with family-level cut-off 90.0% based on 16S rRNA gene sequences) in numerical order.

**Table S3.** Relative abundances of labeled taxa (OTU) based on *mxaF* gene sequences in all fractions (H, heavy; M, middle; L, light) of [$^{12}$C]- and [$^{13}C_1$]- methanol treatments of Substrate SIP experiment.

**Table S4.** Relative abundances of labeled fungal taxa (OTU) based on ITS gene sequences in all fractions (H, heavy; M, middle; L, light) of [$^{12}$C]- and [$^{13}C_1$]-methanol treatments of Substrate SIP experiment.

**Table S5.** Taxonomic affiliation of fungal phylotypes (ITS gene sequences clustered at species-level 97% similarity cut-off) in numerical order.

**Table S6.** Relative abundances of labeled bacterial taxa (OTU) based on 16S rRNA gene sequences in all fractions (H, heavy; M, middle; L, light) of [$^{12}$C]- and [$^{13}C_1$]-methanol treatment at pH 4 of pH SIP experiment.

**Table S7.** Relative abundances of labeled bacterial taxa (OTU) based on 16S rRNA gene sequences in all fractions (H, heavy; M, middle; L, light) of [$^{12}$C]- and [$^{13}C_1$]-methanol treatment at pH 7 of pH SIP experiment.

**Table S8.** Relative abundances of labeled taxa (OTU) based on *mxaF* gene sequences in all fractions (H, heavy; M, middle; L, light) of [$^{12}$C]- and [$^{13}C_1$]- methanol treatments at pH 4 of pH SIP experiment.

**Table S9.** Relative abundances of labeled taxa (OTU) based on *mxaF* gene sequences in all fractions (H, heavy; M, middle; L, light) of [$^{12}$C]- and [$^{13}C_1$]- methanol treatments at pH 7 of pH SIP experiment.

**Table S10.** Relative abundances of labeled fungal taxa (OTU) based on ITS gene sequences in all fractions (H, heavy; M, middle; L, light) of [$^{12}$C]- and [$^{13}C_1$]- methanol treatments at pH 4 of pH SIP experiment.

**Table S11.** Relative abundances of labeled fungal taxa (OTU) based on ITS gene sequences in all fractions (H, heavy; M, middle; L, light) of [$^{12}$C]- and [$^{13}C_1$]- methanol treatments at pH 7 of pH SIP experiment.

**Table S12.** Relative abundance of fungal taxa based on ITS gene sequences from combined pyrosequencing data sets of [$^{12}$C]- and [$^{13}C_u$]-substrate treatments of both SIP experiments.

**Table S13.** Similarity analyses of bacterial communities (family-level with 90.1% cut-off of 16S rRNA gene sequence) of both SIP experiments based on ANOSIM (Analysis of Similarity) and NPMANOVA (non-parametric multivariate analysis of variance).

**Table S14.** Similarity analyses of fungal communities (family-level with 97.0% cut-off of ITS gene sequence) of both SIP experiments based on ANOSIM (Analysis of Similarity) and NPMANOVA (non-parametric multivariate analysis of variance).

**Table S15.** Similarity analyses of *mxaF*-possessing methylotrophic communities (90% cut-off) of both SIP experiments based on ANOSIM (Analysis of Similarity) and NPMANOVA (non-parametric multivariate analysis of variance).

**Table S16.** Relative abundance of bacterial taxa based on 16S rRNA gene sequences from combined pyrosequencing data sets of [$^{12}C$]- and [$^{13}C_u$]-substrate treatments of both SIP experiments.

**Table S17.** Relative abundance of methylotrophic taxa (OTU) based on *mxaF* gene sequences from combined pyrosequencing data sets of [$^{12}C$]- and [$^{13}C_u$]-substrate treatments of both SIP experiments.

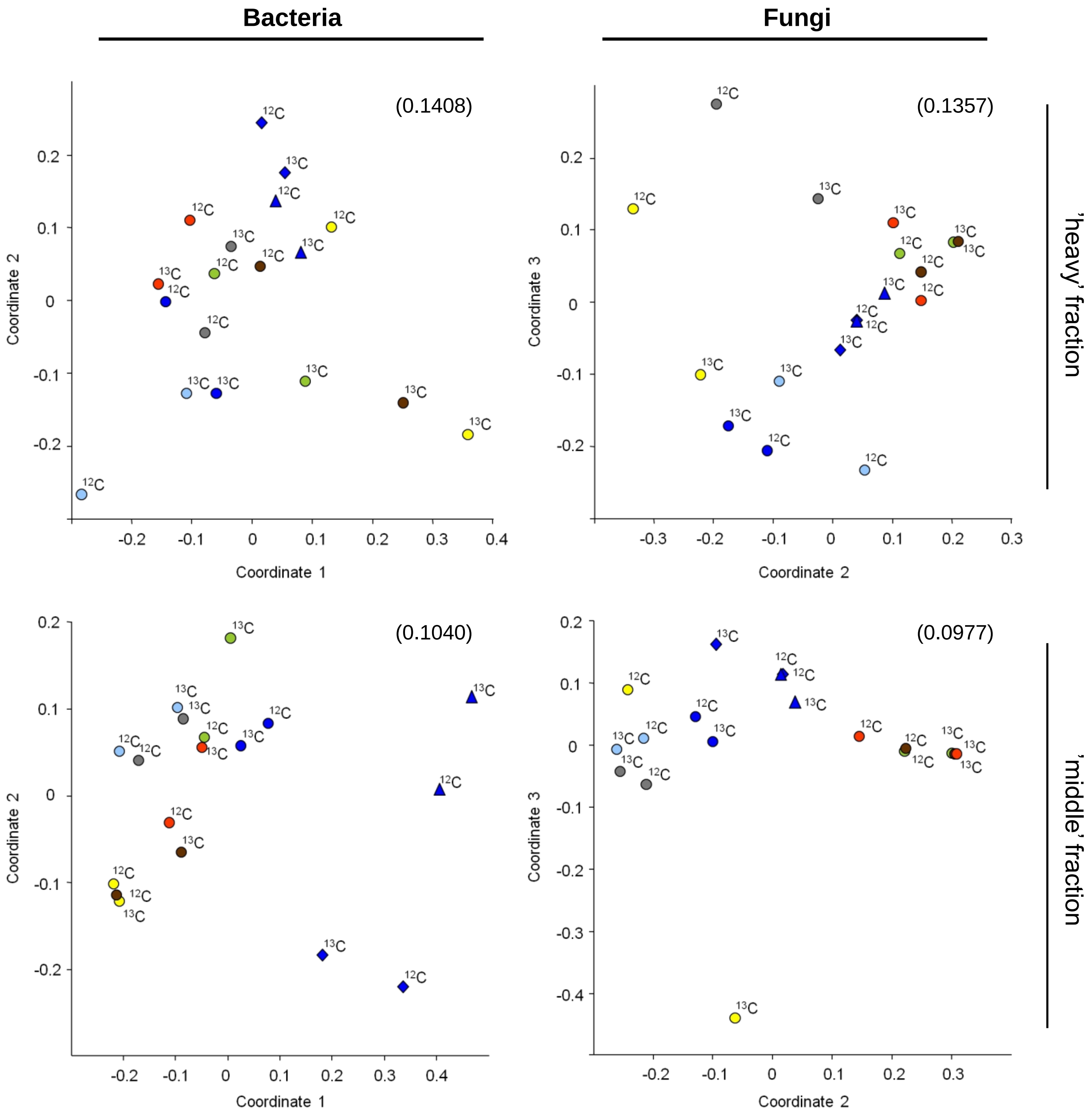


**Figure S1. nMDS analyses of bacterial and fungal communities in the 'heavy' and 'middle' fractions of both SIP experiments.** Figures show analyses of relative abundances of all sequences in 'heavy' and 'middle' fractions of [$^{12}$C]- and [$^{13}$C]-treatments with cut-off values of 90.1% for Bacteria (16S rRNA gene sequences, family-level; reduced dataset, for detailed information see Supplemental Materials and Methods) and 97% for Fungi (ITS gene sequences species-level). Stress values are given in brackets. All analyses are based on Bray-Curtis similarity index.

Symbols according to SIP experiment: ○, substrate SIP; ◇, pH 4; △, pH 7. '$^{12}$C' indicates [$^{12}$C]-substrates and '$^{13}$C' indicates [$^{13}C_u$]-substrates. Symbols according to supplemented [$^{13}C_u$]-substrate: ●, methanol; ●, acetate +; ●, glucose +; ●, xylose +; ●, vanillic acid +; ●, $CO_2$ +; ●, $CO_2$ (cross indicates additional supplementation of [$^{12}$C]-methanol in substrate SIP experiment).

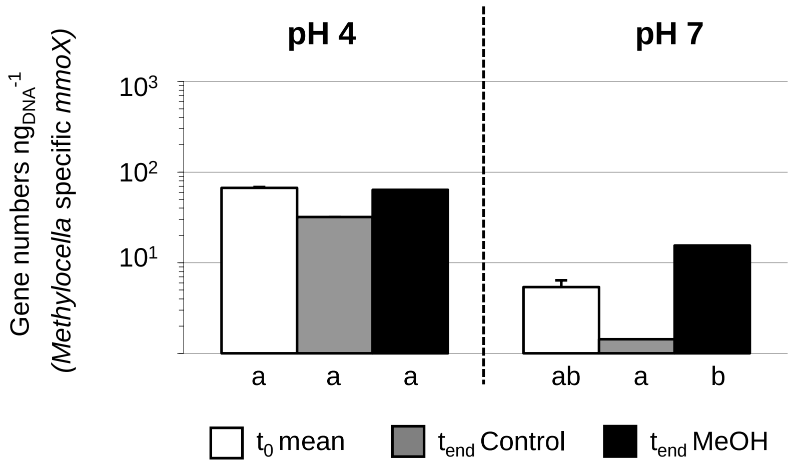


**Figure S2. Gene numbers of *mmoX* genes of treatments with different pH in the pH shift SIP experiment.** Columns, mean values of the experimental replicates. Error bars, standard deviation; if not visible, the variability between replicates was below 0.5%. Different letters, significant differences between samples (t-test; normal distribution was assumed based on the Shapiro-Wilk-test; n=3).

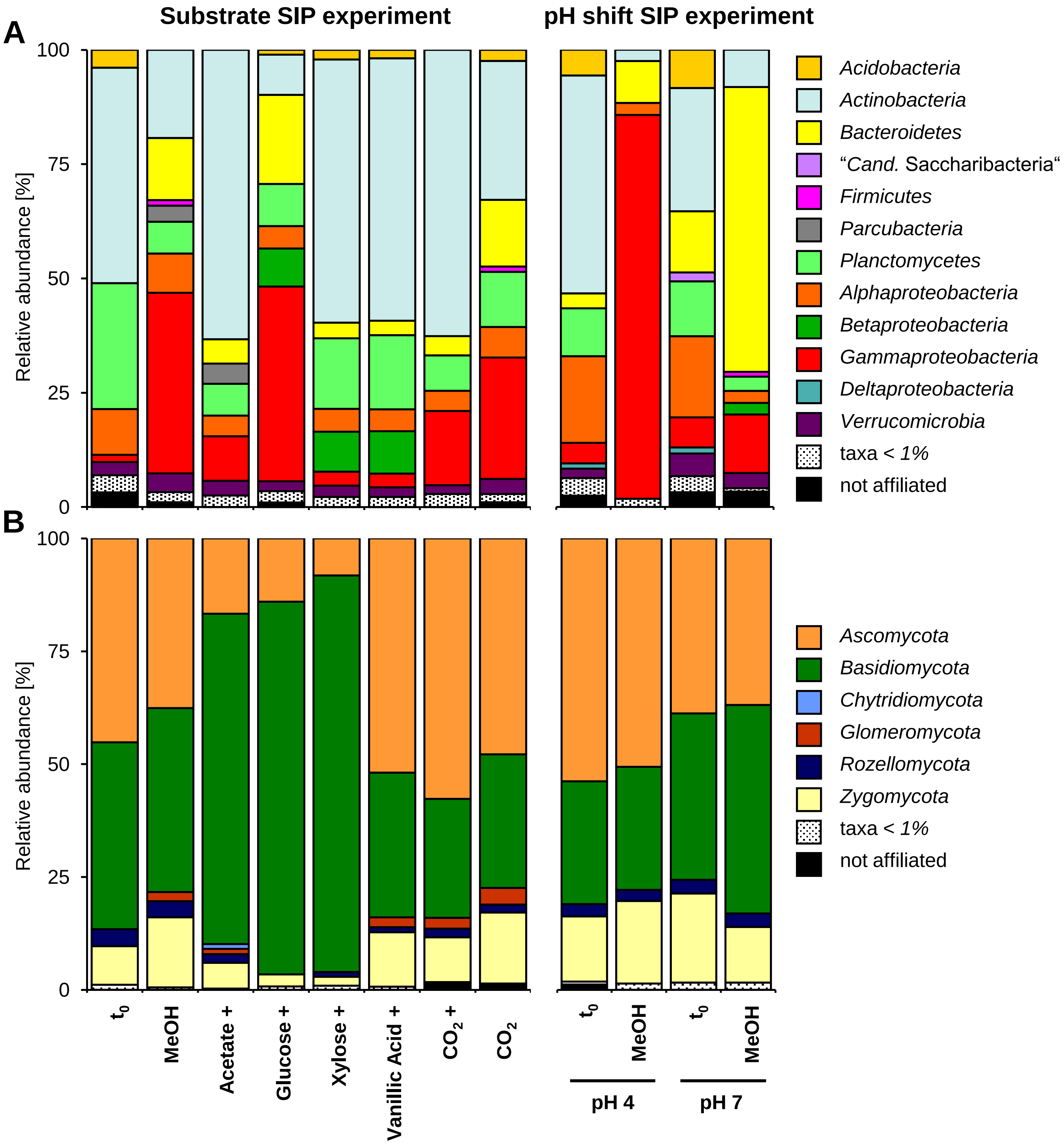


**Figure S3. Bacterial (A) and fungal (B) phyla composition after different substrate or pH treatments.** Shown are relative abundances of combined ($^{12}C$ and $^{13}C$) datasets of 16S rRNA gene sequences (A) and ITS gene sequences (B) derived OTUs from pyrosequencing pools of both SIP experiments at the beginning ($t_0$) and after treatment with substrates or different pH conditions. A cross indicates additional supplementation of methanol in substrate treatments. OTUs were clustered with 90.1 % threshold for 16S rRNA gene sequences (family-level) and 97 % threshold for ITS gene sequences (species-level), respectively. Taxonomic affiliation was confirmed with a GenBank database for 16S rRNA gene sequences and the UNITE database for ITS gene sequences, respectively.

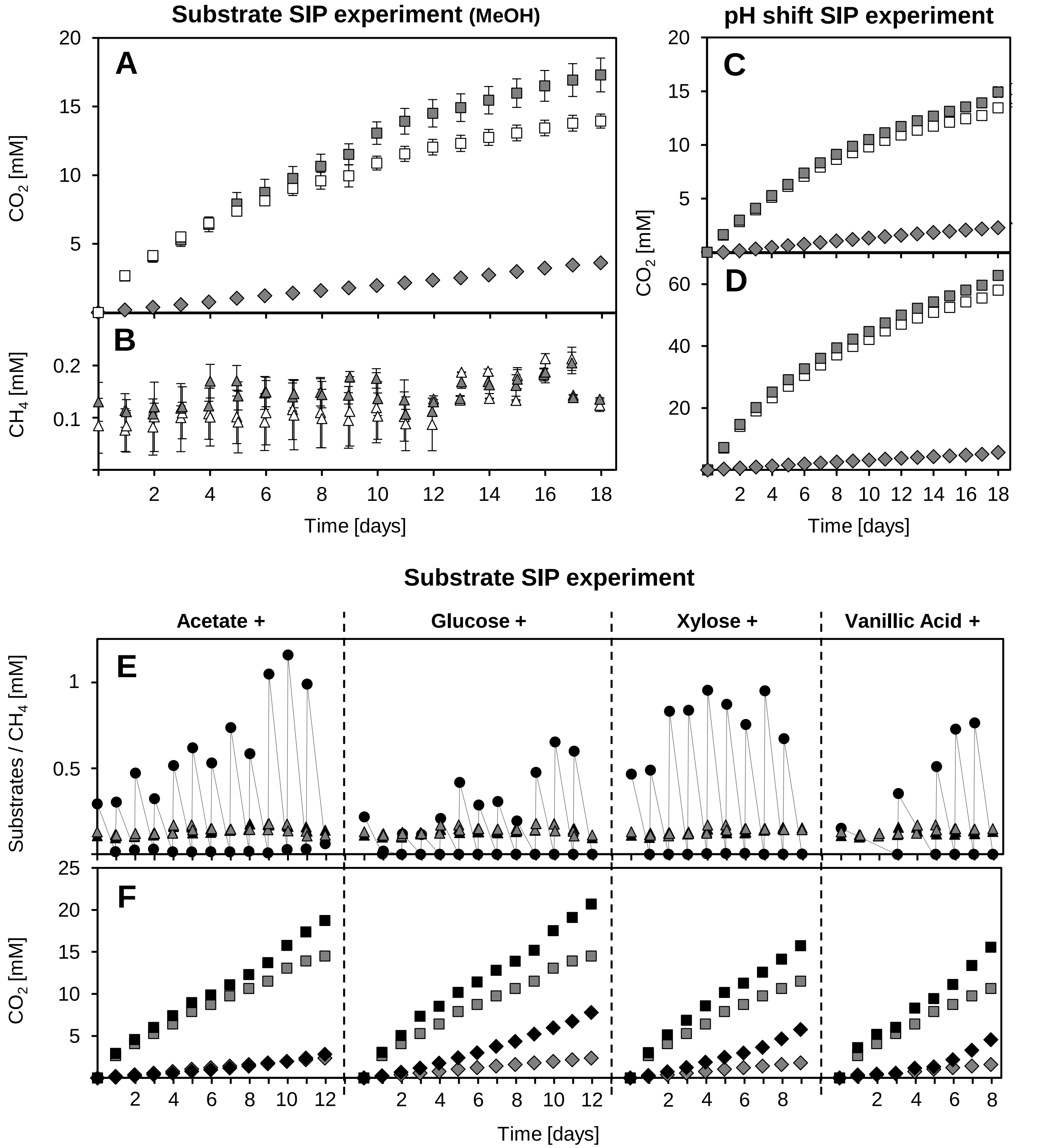


**Figure S4. $CO_2$ formation and conversion of the different multi-carbon substrates in the soil slurry treatments.** Shown are cumulative $^{12}CO_2$ and $^{13}CO_2$ concentrations of substrate SIP experiment treatments pulsed with methanol (A) and other multi-carbon substrates (F), and methanol treatments of the pH shift SIP experiment with pH 4 (C) and pH 7 (D). A cross indicates additional methanol supplementation. Substrate utilization is supposed by the conversion of supplemented substrates (E), methane utilization in the substrate SIP experiment treatments is negligible (B, E). Methanol treatments serve as control treatments for supplemented multi-carbon substrate treatments. All values are mean values of replicates; error bars represent standard deviations. White symbols, unsupplemented control; grey symbols, methanol treatments; black symbols, substrate treatments; ◇, [$^{13}$C]-$CO_2$; □, [$^{12}$C]-$CO_2$; ○, substrates; △, methane.

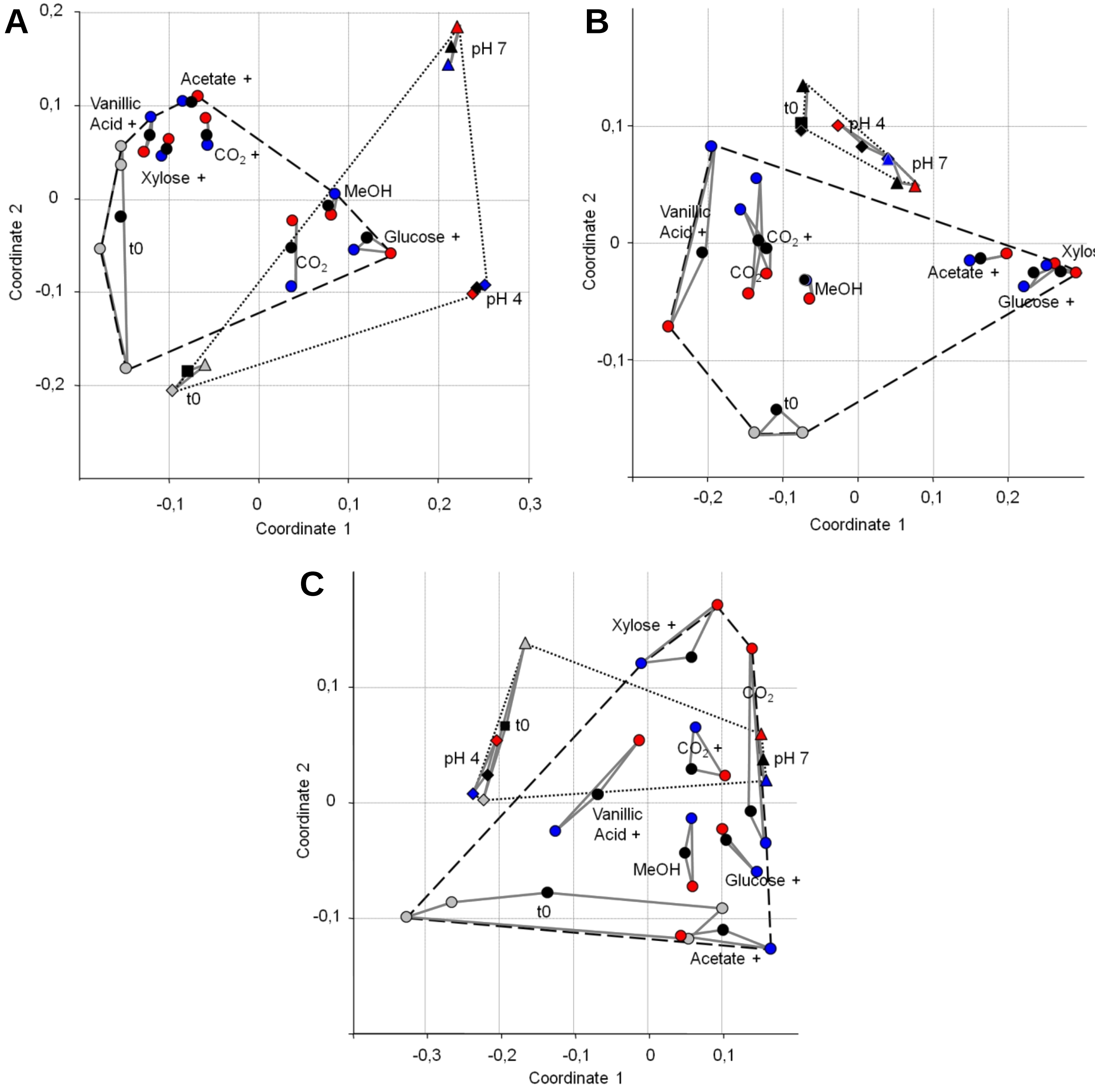


**Figure S5. nMDS analyses of the bacterial community (A), the fungal community (B) and *mxaF* sequences (C) from different substrate or pH treatments.** Figures show analyses of relative abundances of sequences with cut-off values of 90.1% for Bacteria (16S rRNA gene sequences, family-level), 97% for Fungi (ITS gene sequences species-level) and 90% for *mxaF*. Stress values are 0.1002 for bacteria, 0.1245 for fungi and 0.1868 for *mxaF*. All analyses are based on Bray-Curtis similarity index.
Blue, dataset of [$^{12}C$]-replicate; red, dataset of [$^{13}C$]-replicate; black, combined datasets of [$^{12}C$]- and [$^{13}C$]-replicates; grey, $t_0$ replicates; ○, substrate SIP experiment; □, combined $t_0$ datasets of pH shift SIP experiment; ◇, pH 4 treatment; △, pH 7 treatment. A cross indicates additional supplementation of methanol in substrate SIP experiment. Dashed lines indicate convex polygon including all data from substrate SIP experiment. Grey lines indicate convex polygons including all data for each substrate treatment. Dotted lines indicate convex polygon including all data from pH shift SIP experiment.

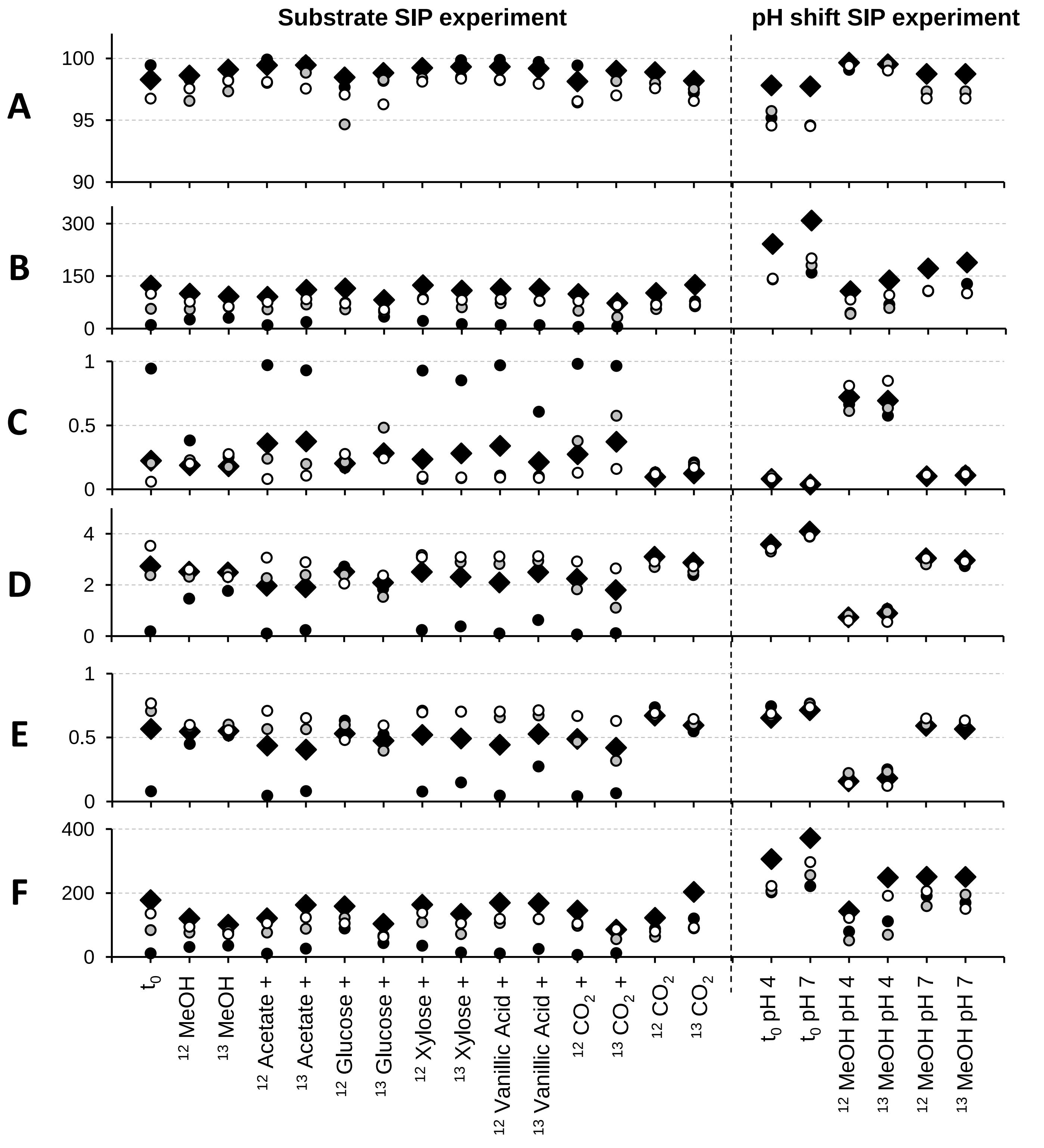


**Figure S6. Diversity and richness estimators of 16S rRNA gene sequences from pyrosequencing amplicon pools at similarity level 90.1% (family level).** Figures indicating coverage (%) (A), numbers of OTUs (B), dominance D (C), Shannon index H (D), equitability J (E) and Chao1 index (F). Shown are values of $t_0$ (no treatment, combined data sets of replicates for substrate SIP experiment $t_0$) and after treatment for both SIP experiments. A $^{12}$ indicates [$^{12}$C]-substrate, $^{13}$ indicates [$^{13}C_u$]-substrate. A cross indicates additional supplementation of methanol in substrate treatments. Symbols: ◆, combined datasets of 'heavy', 'middle' and 'light' fractions; ●, 'heavy' fraction; ●, 'middle' fraction; ○, 'light' fraction.

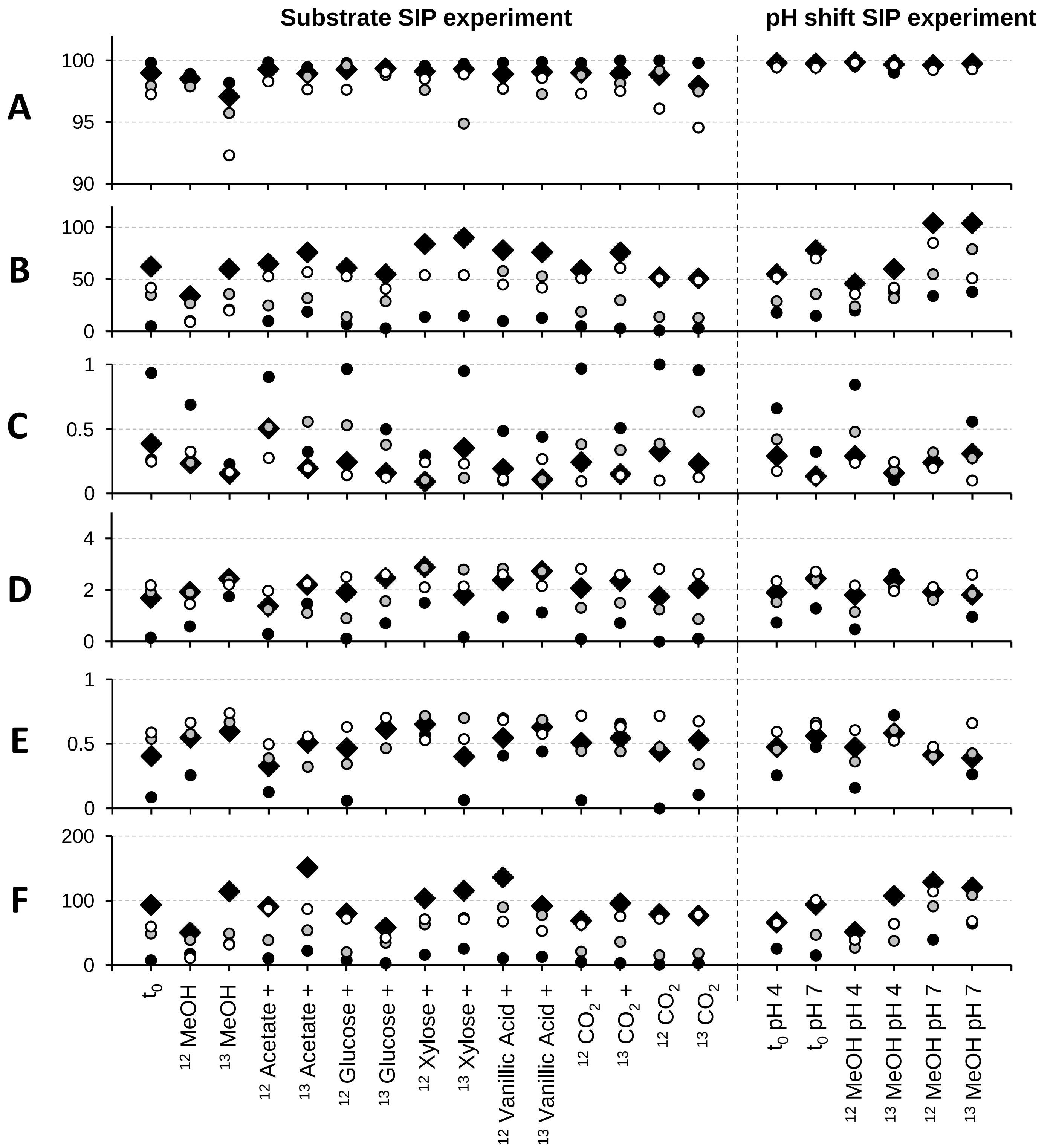


**Figure S7. Diversity and richness estimators of *mxaF* sequences from pyrosequencing amplicon pools at similarity level of 90%.** Figures indicating coverage (%) (A), numbers of OTUs (B), dominance D (C), Shannon index H (D), equitability J (E) and Chao1 index (F). Shown are values of $t_0$ (no treatment, combined datasets of replicates for substrate SIP experiment $t_0$) and after treatment for both SIP experiments. A $^{12}$ indicates [$^{12}$C]-substrate, $^{13}$ indicates [$^{13}C_u$]-substrate. A cross indicates additional supplementation of methanol in substrate treatments. Symbols: ◆, combined data sets of 'heavy', 'middle' and 'light' fractions; ●, 'heavy' fraction; ●, 'middle' fraction; ○, 'light' fraction.

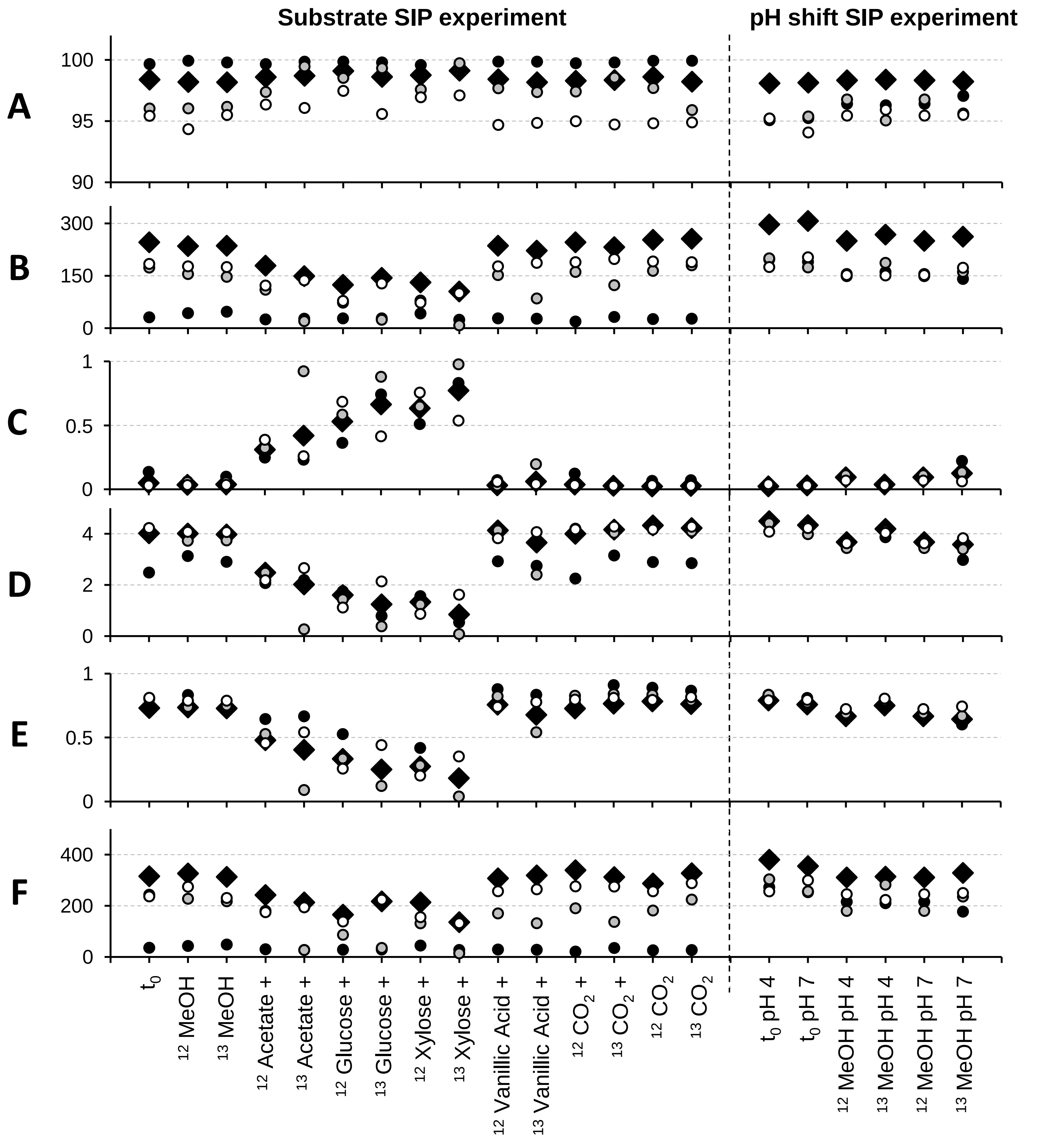


**Figure S8. Diversity and richness estimators of ITS gene sequences from pyrosequencing amplicon pools at similaritiy level of 97% (species level).** Figures indicating coverage (%) (A), numbers of OTUs (B), dominance D (C), Shannon index H (D), equitability J (E) and Chao1 index (F). Shown are values of $t_0$ (no treatment, combined datasets of replicates for substrate SIP experiment $t_0$) and after treatment for both SIP experiments. A $^{12}$ indicates [$^{12}$C]-substrate, $^{13}$ indicates [$^{13}C_u$]-substrate. A cross indicates additional supplementation of methanol in substrate treatments. Symbols: ◆, combined data sets of 'heavy', 'middle' and 'light' fractions; ●, 'heavy' fraction; ●, 'middle' fraction; ○, 'light' fraction.

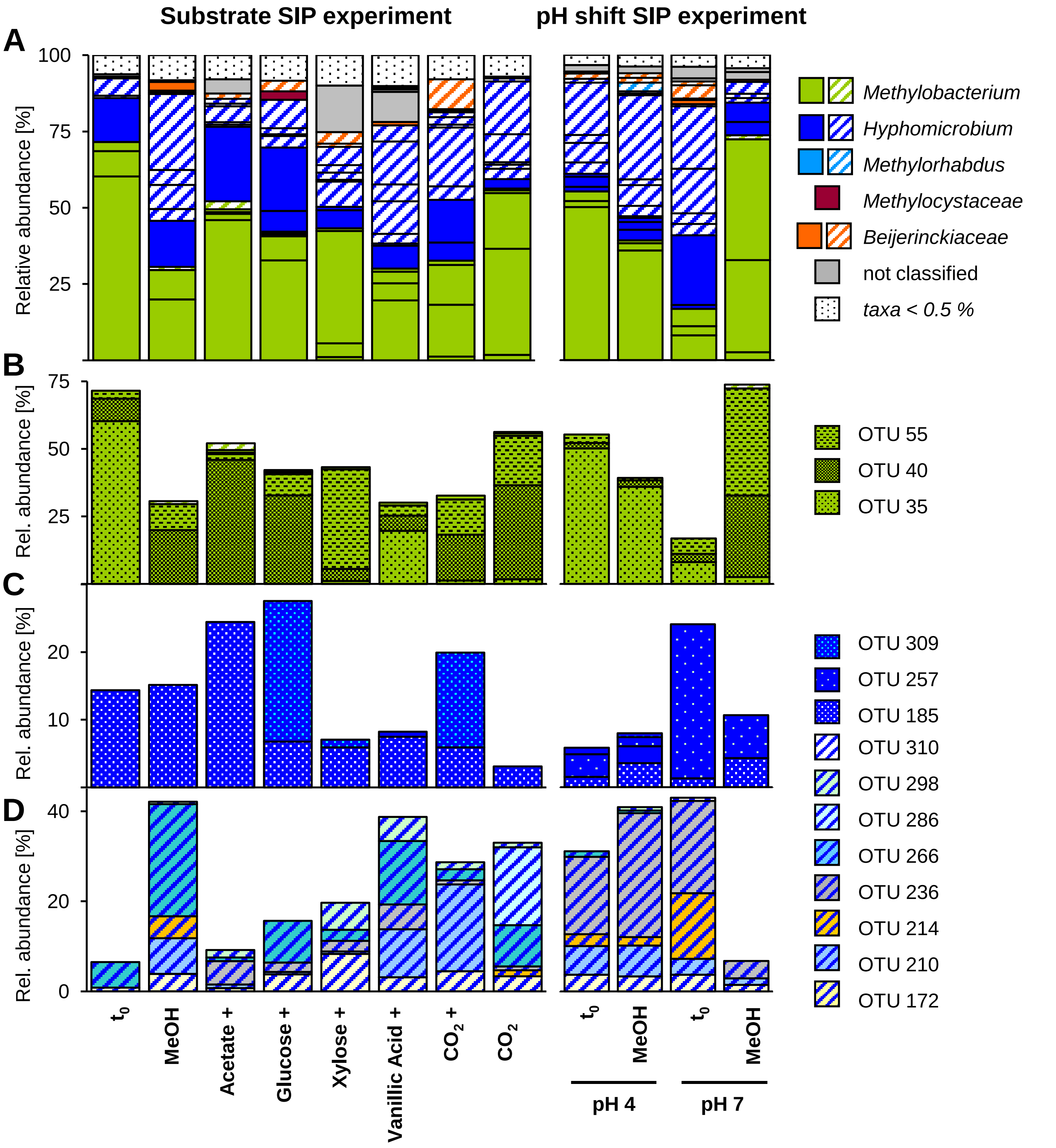


**Figure S9. Composition of the various *mxaF* phylotypes after different substrate or pH treatments.** Shown are relative abundances of combined ($^{12}C$ and $^{13}C$) datasets of all *mxaF*-affiliated phylotypes (A) and in more detail *Methylobacterium*-affilated (B) and *Hyphomicrobium*-affiliated phylotypes (C, D). Same taxonomic affiliation (family) is indicated by equal colors; ambiguous affiliation (i.e. sequence identity with BLASTn <90% as well as ambiguous position in phylogenetic tree) is indicated by shading. Additional [$^{12}C$]-methanol supplementation in substrate SIP experiment is indicated by a cross. Shown are phylotypes with relative abundances ≥0.5% in combined ($^{12}C$ and $^{13}C$) datasets.

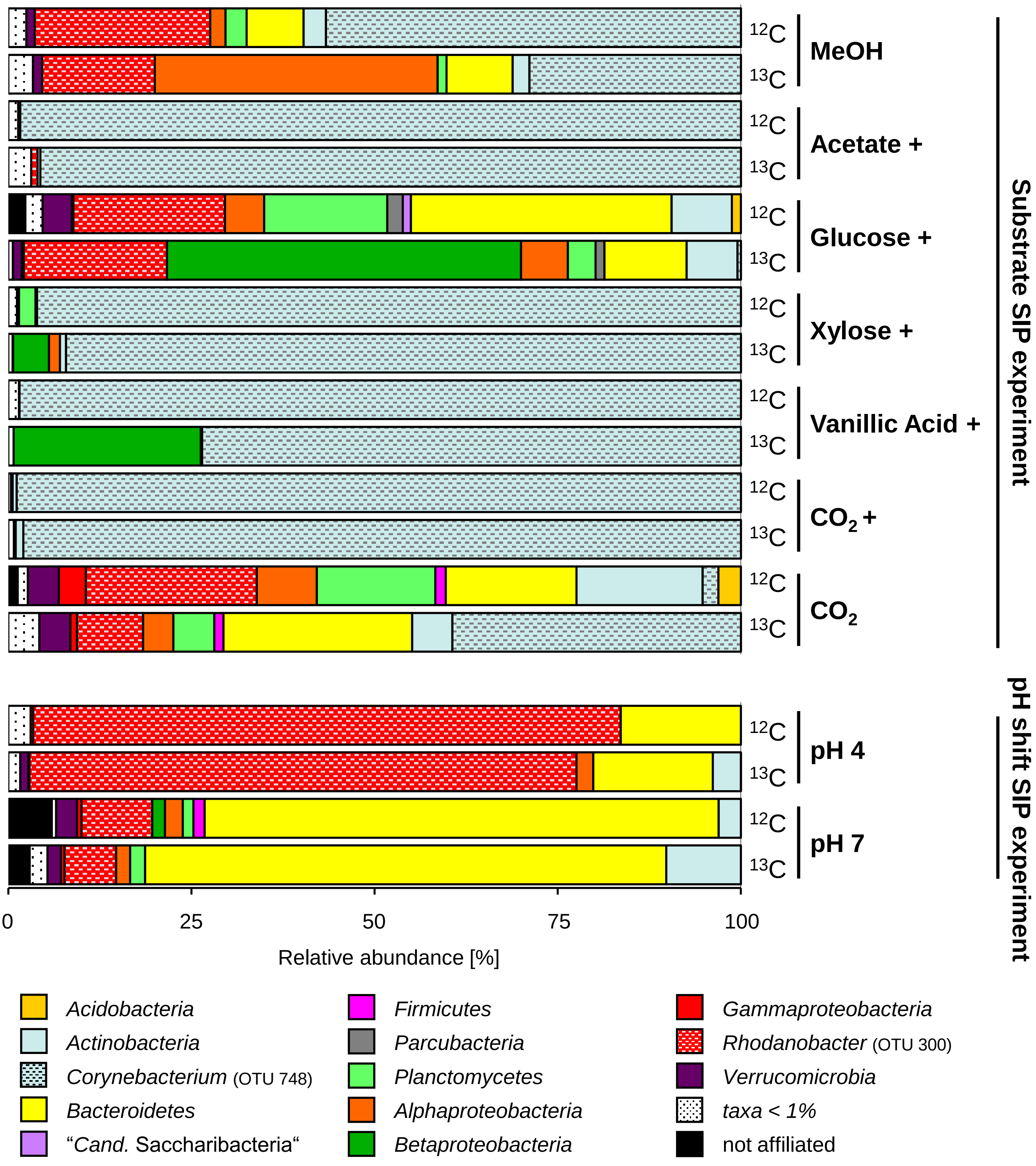


**Figure S10. Bacterial phyla composition in the 'heavy' fractions after different substrate or pH treatments based on all detected phylotypes.** Shown are relative abundances (≥1%) of bacterial phyla based on 16S rRNA gene sequences in the 'heavy' fractions of [$^{12}C$]- and [$^{13}C_u$]-substrate treatments of both SIP experiments. Hatching indicates dominant families in 'heavy' fractions that were removed for the determination of labelled phylotypes. A cross indicates additional supplementation of methanol in substrate treatments. Phylotypes were clustered with 90.1 % threshold for 16S rRNA genes (family-level) and taxonomic affiliation was confirmed with a GenBank database.

**Table S1. Relative abundances of labeled bacterial taxa (OTU) based on 16S rRNA gene sequences in all fractions (H, heavy; M, middle; L, light) of [$^{12}$C]- and [$^{13}C_1$]-methanol treatment of Substrate SIP experiment.** Black faces indicate values that were used for calculating 'labeling proportions' as indicators of relative importance. Bold faces indicate 'labeling proportions' > 5 %.

| Taxonomic affiliation [a] | Labeled OTU | Relative abundance [%] | | | | | | Labeling proportion [%] | |
|---|---|---|---|---|---|---|---|---|---|
| | | $^{12}$C | | | $^{13}$C | | | | |
| | | H | M | L | H | M | L | H | M |
| ***Acidobacteria*** | | | | | | | | | |
| *Acidobacteriaceae* [c] | OTU 542 | 0.0 | 0.2 | 0.2 | 0.7 | 0.3 | 0.4 | 1.3 | |
| *Acidobacterium* [d] | OTU 545 | 0.0 | 0.9 | 0.7 | 0.5 | 0.0 | 0.1 | 0.9 | |
| ***Actinobacteria*** | | | | | | | | | |
| *Acidimicrobiaceae* [c] | OTU 652 | 0.0 | 0.2 | 0.7 | 0.5 | 2.0 | 1.8 | | **12.3** |
| *Kineosporiaceae* [c] | OTU 703 | 0.9 | 2.8 | 1.7 | 0.7 | 4.3 | 1.3 | | **26.2** |
| ***Armatimonadetes*** | | | | | | | | | |
| *Fimbriimonadaceae* [c] | OTU 592 | 0.0 | 0.5 | 0.1 | 0.0 | 1.2 | 0.8 | | **7.4** |
| ***Chlamydiae*** | | | | | | | | | |
| *Parachlamydiaceae* [c] | OTU 108 | 0.0 | 0.0 | 0.0 | 0.5 | 0.0 | 0.0 | 0.9 | |
| ***Firmicutes*** | | | | | | | | | |
| *Bacillus* [d] | OTU 202 | 0.0 | 0.0 | 0.2 | 0.5 | 0.3 | 0.0 | 0.9 | |
| ***Parcubacteria*** [b,e] | OTU 1155 | 0.0 | 0.2 | 0.0 | 1.0 | 0.0 | 0.1 | 1.7 | |
| ***Planctomycetes*** | | | | | | | | | |
| *Planctomycetales* [b] | OTU 836 | 0.0 | 0.0 | 0.7 | 0.2 | 0.5 | 0.0 | | 3.3 |
| *Planctomycetaceae* [c] | OTU 968 | 0.0 | 0.0 | 0.2 | 0.0 | 0.5 | 0.9 | | 3.3 |
| ***Alphaproteobacteria*** | | | | | | | | | |
| *Methylovirgula* [d] | OTU 438 | 3.5 | 0.5 | 2.5 | 52.5 | 3.7 | 1.8 | **91.1** | **22.1** |
| *Sphingomonas* [d] | OTU 449 | 0.0 | 0.0 | 0.5 | 0.5 | 0.1 | 0.5 | 0.9 | |
| *Acetobacteraceae* [c] | OTU 467 | 0.0 | 0.5 | 0.2 | 0.0 | 0.8 | 0.6 | | 4.9 |
| ***Betaproteobacteria*** | | | | | | | | | |
| *Methylophilaceaea* [c] | OTU 360 | 0.0 | 0.0 | 0.0 | 0.7 | 0.0 | 0.0 | 1.3 | |
| ***Verrucomicrobia*** | | | | | | | | | |
| *Spartobacteria* [b] | OTU 6 | 0.0 | 0.9 | 0.7 | 0.7 | 0.4 | 0.7 | 1.3 | |
| *Verrucomicrobiales* [b] | OTU 18 | 1.8 | 2.5 | 1.8 | 0.7 | 3.4 | 1.3 | | **20.5** |
| Percentage of labeled taxa to total fraction [%] | | | | | **58** | **17** | | | |

[a] Taxonomic affiliation was done with BLASTn (November 2015) and is based on the next cultivated hit for each OTU (for further information see Table S8)
[b] Sequence identity of next cultured hit < 90 %, phylogenetic affiliation up to order level
[c] Sequence identity of next cultured hit < 95 %, phylogenetic affiliation up to family level
[d] Sequence identity of next cultured hit ≥ 95 %, phylogenetic affiliation up to genus level
[e] known as "Candidate phylum OD1"

**Table S2. Taxonomic affiliation of bacterial phylotypes (OTUs with family-level cut-off 90.0% based on 16S rRNA gene sequences) in numerical order.** Listed are closest related sequences including cultured and environmental hits (closest related sequences are in grey bold if they are identical with the closest cultured related sequence). BLASTn analyses are based on OTU sequence lengths of 400 – 449 nt.

| OTU | closest cultured related sequence[a] | | | | | | closest related sequence[b] (environmental samples included) | | | |
|---|---|---|---|---|---|---|---|---|---|---|
| | | Q[d] | Id[e] | Accession[c] | Phylum<br>Class<br>Order | Family | | Q[d] | Id[e] | Accession[c] |
| **6** | *Chthoniobacter flavus* | 100 | 90 | NR_115225 | Verrucomicrobia<br>Spartobacteria<br>Chthoniobacterales | Chthoniobacteraceae | uncult. *Verrucomicrobia* | 100 | 97 | JQ368535 |
| **9** | *Chthoniobacter flavus* | 100 | 89 | NR_115225 | Verrucomicrobia<br>Spartobacteria<br>Chthoniobacterales | Chthoniobacteraceae | uncult. *Verrucomicrobia* | 100 | 99 | JQ369834 |
| **17** | *Chthoniobacter flavus* | 100 | 83 | NR_115225 | Verrucomicrobia<br>Spartobacteria<br>Chthoniobacterales | Chthoniobacteraceae | uncult. *Verrucomicrobia* | 100 | 93 | JQ366335 |
| **18** | *Prosthecobacter debontii* | 100 | 84 | AJ966882 | Verrucomicrobia<br>Verrucomicrobiae<br>Verrucomicrobiales | Verrucomicrobiaceae | uncult. *Verrucomicrobia* | 100 | 99 | AM945528 |
| **54** | *Terrimicrobium sacchariphilum* | 99 | 97 | GU129926 | Verrucomicrobia<br>Verrucomicrobiae<br>Chthoniobacterales | n.a. | uncult. *Verrucomicrobia* | 99 | 98 | JF168305 |
| **93** | *Parachlamydia acanthamoebae* | 100 | 95 | JN051144 | Chlamydiae<br>Chlamydiae<br>Chlamydiales | Parachlamydiaceae | uncult. *Chlamydiae* | 96 | 99 | AB504668 |
| **108** | *Parachlamydia acanthamoebae* | 100 | 91 | NR_074972 | Chlamydiae<br>Chlamydiae<br>Chlamydiales | Parachlamydiaceae | "*Candidatus Metachlamydia lacustris*" | 99 | 93 | GQ221847 |
| **132** | *Conexibacter woesei* | 100 | 94 | NR_074830 | Actinobacteria<br>Thermoleophilia<br>Solirubrobacterales | Conexibacteraceae | uncult. *Bacteria* | 96 | 99 | LC011356 |

| OTU | closest cultured related sequence[a] | Q[d] | Id[e] | Accession[c] | Phylum<br>Class<br>Order | Family | closest related sequence[b] (environmental samples included) | Q[d] | Id[e] | Accession[c] |
|---|---|---|---|---|---|---|---|---|---|---|
| **142** | Gaiella occulta | 100 | 93 | NR_118138 | Actinobacteria<br>Rubrobacteria<br>Gaiellales | Gaiellaceae | uncult. *Bacteria* | 100 | 99 | JX100028 |
| **202** | *Bacillus* sp. | 99 | 99 | GU584205 | Firmicutes<br>Bacilli<br>Bacillales | Bacillaceae | *Bacillus* sp. | 99 | 99 | GU584205 |
| **206** | *Paenibacillus* sp. | 100 | 98 | AM162325 | Firmicutes<br>Bacilli<br>Bacillales | Paenibacillaceae | uncult. *Firmicutes* | 100 | 99 | EU810868 |
| **278** | *Povalibacter uvarum* | 100 | 92 | NR_126172 | Proteobacteria<br>Gammaproteobacteria<br>Xanthomonadales | Sinobacteraceae | uncult. *Gammaproteobacterium* | 100 | 99 | JF301246 |
| **280** | *Steroidobacter* sp. | 100 | 98 | KP185148 | Proteobacteria<br>Gammaproteobacteria<br>Xanthomonadales | Sinobacteraceae | uncult. *Gammaproteobacterium* | 100 | 99 | HF952308 |
| **300** | *Rhodanobacter* sp. | 99 | 99 | JX483760 | Proteobacteria<br>Gammaproteobacteria<br>Xanthomonadales | Xanthomonadaceae | uncult. *Gammaproteobacterium* | 99 | 99 | HF952332 |
| **343** | *Rhodanobacter* sp. | 72 | 98 | AF250415 | Proteobacteria<br>Gammaproteobacteria<br>Xanthomonadales | Xanthomonadaceae | uncult. *Gammaproteobacterium (Xanthomonadales)* | 72 | 98 | LC016556 |
| **358** | *Methylophilus methylotrophus* | 99 | 96 | KF911346 | Proteobacteria<br>Betaproteobacteria<br>Methylophilales | Methylophilaceae | uncult. *Betaproteobacterium* | 99 | 98 | JQ371286 |
| **360** | *Methylophilus* sp. | 100 | 91 | CP012020 | Proteobacteria<br>Betaproteobacteria<br>Methylophilales | Methylophilaceae | uncult. *Betaproteobacterium* | 100 | 98 | HG529147 |
| **361** | *Burkholderia* sp. | 100 | 99 | AB627009 | Proteobacteria<br>Betaproteobacteria<br>Burkholderiales | Burkholderiaceae | uncult. *Bacterium* | 100 | 99 | KP726299 |

| OTU | closest cultured related sequence[a] | Q[d] | Id[e] | Accession[c] | Phylum Class Order | Family | closest related sequence[b] (environmental samples included) | Q[d] | Id[e] | Accession[c] |
|---|---|---|---|---|---|---|---|---|---|---|
| **379** | *Acinetobacter* sp. | 100 | 95 | JF715439. | Proteobacteria Gammaproteobacteria Pseudomonadales | Moraxellaceae | uncult. *Gammaproteobacterium* | 100 | 99 | HM113016 |
| **431** | *Caulobacter mirabilis* | 98 | 95 | LN867237 | Proteobacteria Alphaproteobacteria Caulobacterales | Caulobacteraceae | uncult. *Alphaproteobacterium* | 94 | 99 | JN032148 |
| **438** | *Methylovirgula ligni* | 99 | 98 | FM252035 | Proteobacteria Alphaproteobacteria Rhizobiales | Beijerinckiaceae | uncult. *Alphaproteobacterium* | 99 | 99 | AM992784 |
| **449** | *Sphingomonas* sp. | 97 | 99 | KJ654808 | Proteobacteria Alphaproteobacteria Sphingomonadales | Sphingomonadaceae | uncult. *Alphaproteobacterium* (*Sphingobium* sp.) | 97 | 99 | JN860403 |
| **450** | *Paracoccus* sp. | 100 | 99 | LN879490 | Proteobacteria Alphaproteobacteria Rhodobacterales | Rhodobacteraceae | *Paracoccus* sp. | 100 | 99 | LN879490 |
| **460** | *Skermanella* sp. | 99 | 94 | KF561388 | Proteobacteria Alphaproteobacteria Rhodospirillales | Rhodospirillaceae | uncult. *Alphaproteobacterium* | 99 | 99 | EU849340 |
| **467** | *Acidisphaera* sp. | 100 | 94 | KM369919 | Proteobacteria Alphaproteobacteria Rhodospirillales | Acetobacteraceae | uncult. *Alphaproteobacterium* | 100 | 98 | JQ383603 |
| **479** | *Phaeospirillum* sp. | 97 | 94 | HG417062 | Proteobacteria Alphaproteobacteria Rhodospirillales | Rhodospirillaceae | uncult. *Alphaproteobacterium* | 97 | 97 | JQ386981 |
| **528** | *Solibacter usitatus* | 99 | 93 | CP000473 | Acidobacteria Solibacteres Solibacterales | Solibacteraceae | uncult. *Acidobacterium* | 99 | 99 | JX981716 |
| **542** | *Acidipila* sp. | 100 | 93 | KM083127 | Acidobacteria Acidobacteria Acidobacteriales | Acidobacteriaceae | uncult. *Acidobacterium* | 100 | 99 | GU598605 |

| OTU | closest cultured related sequence[a] | Q[d] | Id[e] | Accession[c] | Phylum / Class / Order | Family | closest related sequence[b] (environmental samples included) | Q[d] | Id[e] | Accession[c] |
|---|---|---|---|---|---|---|---|---|---|---|
| **545** | *Acidobacterium capsulatum* | 98 | 98 | AM086241 | Acidobacteria / Acidobacteria / Acidobacteriales | Acidobacteriaceae | uncult. *Acidobacterium* | 98 | 99 | JF300928 |
| **592** | *Fimbriimonas ginsengisoli* | 100 | 93 | CP007139 | Armatimonadetes / Fimbriimonadia / Fimbriimonadales | Fimbriimonadaceae | uncult. *Armatimonadetes* | 100 | 98 | JN851205 |
| **615** | *Gelria glutamica* | 99 | 85 | NR_041819 | Firmicutes / Clostridia / Thermoanaerobacterales | Thermo-anaerobacteraceae | uncult. *Bacterium* | 95 | 99 | HQ629022 |
| **652** | *Aciditerrimonas ferrireducens* | 98 | 92 | NR_112972 | Actinobacteria / Acidimicrobiia / Acidimicrobiales | Acidimicrobiaceae | uncult. *Actinobacterium* | 98 | 97 | HQ845571 |
| **654** | *Aciditerrimonas ferrireducens* | 99 | 92 | NR_112972 | Actinobacteria / Acidimicrobiia / Acidimicrobiales | Acidimicrobiaceae | uncult. *Actinobacterium* | 99 | 98 | JQ376471 |
| **656** | *Aciditerrimonas ferrireducens* | 100 | 93 | NR_112972 | Actinobacteria / Acidimicrobiia / Acidimicrobiales | Acidimicrobiaceae | uncult. *Actinobacterium* | 100 | 99 | JQ376189 |
| **668** | *Aciditerrimonas ferrireducens* | 100 | 89 | NR_112972 | Actinobacteria / Acidimicrobiia / Acidimicrobiales | Acidimicrobiaceae | uncult. *Actinobacterium* | 92 | 99 | JX204369 |
| **703** | *Kineosporia* sp. | 100 | 94 | FM886845 | Actinobacteria / Actinobacteria / Actinomycetales | Kineosporiaceae | uncult. *Actinobacterium* | 100 | 99 | FJ661525 |
| **721** | *Leifsonia xyli* | 97 | 99 | HG794292 | Actinobacteria / Actinobacteria / Micrococcales | Microbacteriaceae | *Leifsonia xyli* | 97 | 99 | HG794292 |
| **723** | *Pseudonocardia spinosispora* | 98 | 98 | NR_025367 | Actinobacteria / Actinobacteria / Pseudonocardiales | Pseudonocardiaceae | uncult. *Actinobacterium* | 98 | 98 | JN615726 |

| OTU | closest cultured related sequence[a] | | | | | | closest related sequence[b] (environmental samples included) | | | |
|---|---|---|---|---|---|---|---|---|---|---|
| | | Q[d] | Id[e] | Accession[c] | Phylum Class Order | Family | | Q[d] | Id[e] | Accession[c] |
| **748** | *Corynebacterium nuruki* | 100 | 93 | NR_117816 | Actinobacteria Actinobacteria Corynebacteriales | Corynebacteriaceae | uncult. *Actinobacterium* | 100 | 94 | FN667204 |
| **750** | *Mycobacterium branderi* | 100 | 98 | JX266696 | Actinobacteria Actinobacteria Corynebacteriales | Mycobacteriaceae | uncult. *Actinobacterium* | 100 | 98 | KF530052 |
| **752** | *Corynebacterium nuruki* | 99 | 96 | NR_117816 | Actinobacteria Actinobacteria Corynebacteriales | Corynebacteriaceae | uncult. *Actinobacterium* | 99 | 97 | JF208992 |
| **758** | *Actinospica robiniae* | 99 | 99 | NR_042364 | Actinobacteria Actinobacteria Catenulisporales | Actinospicaceae | uncult. *Actinobacterium* | 99 | 99 | AB180777 |
| **796** | *Tepidisphaera mucosa* | 99 | 92 | KM052380 | Planctomycetes Phycisphaerae | Tepidisphaeraceae | uncult. *Bacterium* | 99 | 99 | KJ407548 |
| **800** | *Tepidisphaera mucosa* | 100 | 89 | KM052380 | Planctomycetes Phycisphaerae | Tepidisphaeraceae | uncult. *Planctomycetes* | 100 | 98 | JQ663701 |
| **816** | *Actinoallomurus* sp. | 70 | 92 | KR781096 | Actinobacteria Actinobacteria Streptosporangiales | Thermomonosporaceae | uncult. *Bacterium* | 71 | 95 | FQ744029 |
| **836** | *Pirellula* sp. | 99 | 88 | X81947 | Planctomycetes Planctomycetia Planctomycetales | Planctomycetaceae | "*Candidatus* Anammoximicrobium moscowii" | 99 | 90 | KC467065 |
| **840** | *Thermogutta terrifontis* | 100 | 86 | KC867694 | Planctomycetes Planctomycetia Planctomycetales | Planctomycetaceae | uncult. *Planctomycetes* | 100 | 93 | LK025317 |
| **844** | *Thermogutta hypogea* | 99 | 88 | KC867695 | Planctomycetes Planctomycetia Planctomycetales | Planctomycetaceae | uncult. *Planctomycetes* | 99 | 90 | LK025387 |

| OTU | closest cultured related sequence[a] | | | | | | closest related sequence[b] (environmental samples included) | | | |
|---|---|---|---|---|---|---|---|---|---|---|
| | | Q[d] | Id[e] | Accession[c] | Phylum Class Order | Family | | Q[d] | Id[e] | Accession[c] |
| **857** | *Planctopirus limnophilus* | 100 | 88 | NR_074670 | Planctomycetes Planctomycetia Planctomycetales | Planctomycetaceae | *Gimesia maris* | 100 | 93 | KF228168 |
| **885** | *Nostocoida limicola* III | 100 | 94 | AF244752 | Planctomycetes Planctomycetia Planctomycetales | Planctomycetaceae | *Nostocoida limicola* III | 100 | 94 | AF244752 |
| **904** | *Singulisphaera* sp. | 100 | 92 | GQ889439 | Planctomycetes Planctomycetia Planctomycetales | Planctomycetaceae | uncult. *Planctomycetes* | 100 | 92 | HM748671 |
| **923** | *Gemmata* sp. | 99 | 90 | GQ889476 | Planctomycetes Planctomycetia Planctomycetales | Planctomycetaceae | uncult. *Planctomycetes* | 99 | 95 | AB127858 |
| **927** | *Gemmata* sp. | 100 | 89 | GQ889455 | Planctomycetes Planctomycetia Planctomycetales | Planctomycetaceae | uncult. *Planctomycetes* | 100 | 93 | LK025439 |
| **939** | *Gemmata* sp. | 100 | 90 | GQ889455 | Planctomycetes Planctomycetia Planctomycetales | Planctomycetaceae | uncult. *Planctomycetes* | 100 | 93 | LK025552 |
| **945** | *Isosphaera* sp. | 100 | 89 | GQ889426 | Planctomycetes Planctomycetia Planctomycetales | Planctomycetaceae | uncult. *Planctomycetes* | 100 | 89 | FJ405890 |
| **951** | *Gemmata* sp. | 99 | 91 | GQ889466 | Planctomycetes Planctomycetia Planctomycetales | Planctomycetaceae | *Gemmata* sp. | 99 | 91 | GQ889466 |
| **968** | *Zavarzinella formosa* | 99 | 90 | NR_042465 | Planctomycetes Planctomycetia Planctomycetales | Planctomycetaceae | uncult. *Planctomycetes* | 99 | 98 | LK024693 |
| **1014** | *Ferruginibacter lapsinanis* | 100 | 97 | NR_044589 | Bacteroidetes Sphingobacteriia Sphingobacteriales | Chitinophagaceae | *Ferruginibacter lapsinanis* | 100 | 97 | NR_044589 |

| OTU | closest cultured related sequence[a] | Q[d] | Id[e] | Accession[c] | Phylum<br>Class<br>Order | Family | closest related sequence[b] (environmental samples included) | Q[d] | Id[e] | Accession[c] |
|---|---|---|---|---|---|---|---|---|---|---|
| **1018** | *Heliimonas saccharivorans* | 100 | 95 | JX458466 | Bacteroidetes<br>Sphingobacteriia<br>Sphingobacteriales | Chitinophagaceae | *Heliimonas saccharivorans* | 100 | 95 | JX458466 |
| **1020** | *Chitinophaga* sp. | 99 | 96 | JF806524 | Bacteroidetes<br>Sphingobacteriia<br>Sphingobacteriales | Chitinophagaceae | *Chitinophaga* sp. | 99 | 96 | JF806524 |
| **1045** | *Chryseobacterium* sp. | 100 | 99 | JF700394 | Bacteroidetes<br>Flavobacteriia<br>Flavobacteriales | Flavobacteriaceae | *Chryseobacterium* sp. | 100 | 99 | JF700394 |
| **1073** | *Mucilaginibacter mallensis* | 100 | 98 | NR_116978 | Bacteroidetes<br>Sphingobacteriia<br>Sphingobacteriales | Sphingobacteriaceae | *Mucilaginibacter mallensis* | 100 | 98 | NR_116978 |
| **1078** | *Sphingobacterium* sp. | 100 | 99 | LN867309 | Bacteroidetes<br>Sphingobacteriia<br>Sphingobacteriales | Sphingobacteriaceae | *Sphingobacterium* sp. | 100 | 99 | LN867309 |
| **1094** | *Pedobacter westerhofensis* | 97 | 91 | NR_042602 | Bacteroidetes<br>Sphingobacteriia<br>Sphingobacteriales | Sphingobacteriaceae | *Pedobacter westerhofensis* | 97 | 91 | NR_042602 |
| **1098** | *Solitalea koreensis* | 100 | 86 | NR_044568 | Bacteroidetes<br>Sphingobacteriia<br>Sphingobacteriales | Sphingobacteriaceae | uncult. *Bacteroidetes* | 95 | 90 | HQ663372 |
| **1108** | *Cytophaga hutchinsonii* | 100 | 99 | NR_102866 | Bacteroidetes<br>Cytophagia<br>Cytophagales | Cytophagaceae | *Cytophaga hutchinsonii* | 100 | 99 | NR_102866 |
| **1116** | "*Candidatus Saccharimonas aalborgensis*" | 99 | 86 | CP005957 | Candidatus Saccharibacteria<br>*Candidatus Saccharimonas* | n.a. | uncult. *Bacterium* | 99 | 90 | EF197025 |
| **1136** | "*Candidatus Saccharimonas aalborgensis*" | 99 | 83 | KC495062 | Parcubacteria<br>*Candidatus Sonnebornia.* | n.a. | uncult. *Parcubacterium* | 99 | 87 | HQ290495 |

| OTU | closest cultured related sequence[a] | Q[c] | Id[d] | Accession[e] | Phylum Class Order | Family | closest related sequence[b] (environmental samples included) | Q[d] | Id[e] | Accession[c] |
|---|---|---|---|---|---|---|---|---|---|---|
| **1140** | "*Candidatus Saccharimonas aalborgensis*" | 100 | 85 | KC495062 | Parcubacteria *Campbellbacteria.* | n.a. | "*Candidatus Saccharimonas aalborgensis*" | 100 | 85 | KC495062 |
| **1155** | "*Candidatus Saccharimonas aalborgensis*" | 100 | 83 | KC495061 | Parcubacteria *Campbellbacteria.* | n.a. | uncult. *Parcubacterium* | 98 | 86 | CP011215 |
| **1158** | Parcubacterium | 100 | 84 | CP011215 | Parcubacteria | n.a. | uncult. *Parcubacterium* | 100 | 95 | JN128702 |

[a] Closest cultured sequences includes validly published names of bacterial species with known strains. No Candidates are listed here with the exception for the candidates' phyla *Saccharibacteria* ($OTU_{16S}$1116) and *Parcubateria* ($OTU_{16S}$1136, $OTU_{16S}$1140, $OTU_{16S}$1155).
[b] Closest related sequences includes uncultured hits and are affiliated at least to phylum level using NCBI and SILVA SSU databases.
[c] Query values of sequences [%].
[d] Maximum sequence identity [%] in BLASTn.
[e] Accession number of closest sequence hit.
[f] n.a. – no further details available

**Table S3. Relative abundances of labeled taxa (OTU) based on *mxaF* gene sequences in all fractions (H, heavy; M, middle; L, light) of [$^{12}$C]- and [$^{13}C_1$]-methanol treatments of Substrate SIP experiment.** Black faces indicate values that were used for calculating 'labeling proportions' as indicators of relative importance. Bold faces indicate 'labeling proportions' > 5 %.

| Taxonomic affiliation [a] | Labeled OTU | Relative abundance [%] | | | | | | Labeling proportion [%] | |
|---|---|---|---|---|---|---|---|---|---|
| | | $^{12}$C | | | $^{13}$C | | | | |
| | | H | M | L | H | M | L | H | M |
| ***Methylobacteriaceae*** | | | | | | | | | |
| | OTU 40 | 0.2 | 35.3 | 7.5 | 24.9 | 32.4 | 3.1 | **25.6** | |
| | OTU 58 | 0.0 | 0.0 | 0.0 | 0.0 | 1.5 | 0.0 | | 3.6 |
| | OTU 79 | 0.0 | 0.0 | 1.9 | 0.0 | 0.6 | 0.0 | | 1.4 |
| | OTU 107 | 0.0 | 0.8 | 0.0 | 0.0 | 1.2 | 0.0 | | 2.9 |
| | OTU 108 | 0.0 | 0.2 | 0.0 | 0.6 | 0.9 | 0.0 | 0.6 | 2.1 |
| | OTU 153 | 0.0 | 0.0 | 1.9 | 0.0 | 0.9 | 0.0 | | 2.1 |
| *ambiguous* [b] | | | | | | | | | |
| | OTU 0 | 0.0 | 0.0 | 0.0 | 0.6 | 0.0 | 0.0 | 0.6 | |
| | OTU 2 | 0.0 | 0.0 | 0.0 | 0.6 | 0.0 | 0.0 | 0.6 | |
| | OTU 9 | 0.0 | 0.0 | 0.0 | 0.2 | 1.5 | 0.0 | | 3.6 |
| | OTU 222 | 0.0 | 2.1 | 1.9 | 1.6 | 0.6 | 0.0 | 1.7 | |
| ***Hyphomicrobiaceae*** | | | | | | | | | |
| | OTU 185 | 0.4 | 10.6 | 47.2 | 30.2 | 20.2 | 11.6 | **31.0** | **47.1** |
| | OTU 202 | 0.2 | 0.0 | 1.9 | 0.0 | 2.1 | 0.0 | | **5.0** |
| *ambiguous* [b] | | | | | | | | | |
| | OTU 177 | 0.0 | 0.4 | 0.0 | 0.0 | 0.6 | 0.0 | | 1.4 |
| | OTU 197 | 0.0 | 0.2 | 0.0 | 0.0 | 0.9 | 0.0 | | 2.1 |
| | OTU 210 | 0.0 | 1.3 | 30.2 | 25.4 | 4.0 | 0.0 | **26.0** | **9.3** |
| | OTU 289 | 0.0 | 0.0 | 0.0 | 0.0 | 0.9 | 0.0 | | 2.1 |
| | OTU 316 | 0.0 | 0.0 | 0.0 | 0.0 | 0.9 | 0.0 | | 2.1 |
| ***Methylorhabdus*** | | | | | | | | | |
| | OTU 190 | 0.0 | 0.0 | 0.0 | 2.2 | 0.0 | 0.0 | 2.3 | |
| *ambiguous* [b] | | | | | | | | | |
| | OTU 18 | 0.0 | 0.0 | 0.0 | 1.8 | 0.0 | 0.0 | 1.9 | |
| ***Beijerinckiaceae*** | | | | | | | | | |
| | OTU 144 | 0.2 | 0.0 | 0.0 | 9.5 | 2.8 | 0.8 | **9.7** | **6.4** |
| | OTU 152 | 0.0 | 0.0 | 0.0 | 0.4 | 0.6 | 0.0 | | 1.4 |
| | OTU 374 | 0.0 | 0.0 | 0.0 | 0.0 | 0.6 | 0.0 | | 1.4 |
| *ambiguous* [b] | | | | | | | | | |
| | OTU 352 | 0.0 | 0.0 | 0.0 | 0.0 | 0.9 | 0.0 | | 2.1 |
| ***not classified*** | | | | | | | | | |
| | OTU 275 | 0.0 | 0.0 | 0.0 | 0.0 | 1.5 | 0.0 | | 3.6 |
| Percentage of labeled taxa to total fraction [%] | | | | | **97** | **43** | | | |

[a] Taxonomic affiliation was done with BLASTn (December 2015; (for further information see Table S2) and confirmed by positioning in phylogenetic tree (data not shown)
[b] Sequence identity with BLASTn < 90 % as well as ambiguous position in phylogenetic tree (for further information see Table S2)

**Table S4. Relative abundances of labeled fungal taxa (OTU) based on ITS gene sequences in all fractions (H, heavy; M, middle; L, light) of [$^{12}$C]- and [$^{13}C_1$]-methanol treatments of Substrate SIP experiment.** Black faces indicate values that were used for calculating 'labeling proportions' as indicators of relative importance. Bold faces indicate 'labeling proportions' > 5 %.

| Taxonomic affiliation [a] | Labeled OTU | Relative abundance [%] | | | | | | Labeling proportion [%] | |
|---|---|---|---|---|---|---|---|---|---|
| | | $^{12}$C | | | $^{13}$C | | | | |
| | | H | M | L | H | M | L | H | M |
| ***Ascomycota*** | | | | | | | | | |
| *Saccharomycetes* | OTU 2 | 0.0 | 14.2 | 5.2 | 7.6 | 8.6 | 5.1 | **9.9** | |
| *Oidiodendron* | OTU 9 | 0.0 | 0.7 | 1.0 | 0.6 | 0.0 | 0.4 | 0.8 | |
| *Penicillium* | OTU 10 | 0.0 | 1.1 | 3.2 | 14.7 | 0.8 | 4.1 | **19.2** | |
| *Paecilomyces* | OTU 14 | 0.0 | 0.1 | 0.4 | 2.5 | 0.0 | 0.1 | 3.3 | |
| *Bionectria* | OTU 24 | 0.0 | 0.4 | 0.7 | 0.0 | 0.7 | 1.2 | | 4.3 |
| *Oidiodendron* | OTU 32 | 4.1 | 0.3 | 0.9 | 5.4 | 0.1 | 1.0 | **7.1** | |
| *Geomyces* | OTU 55 | 0.0 | 0.3 | 0.8 | 0.1 | 0.6 | 0.3 | | 3.5 |
| *Hyaloscyphaceae* | OTU 135 | 0.0 | 0.1 | 0.1 | 0.0 | 1.1 | 0.1 | | **7.0** |
| *Penicillium* | OTU 188 | 0.0 | 0.0 | 0.0 | 2.9 | 0.0 | 0.0 | 3.8 | |
| ***Basidiomycota*** | | | | | | | | | |
| *Leucosporidiales* | OTU 15 | 0.0 | 4.4 | 2.6 | 1.2 | 3.3 | 2.4 | 1.6 | |
| *Syzygospora* | OTU 22 | 0.0 | 2.5 | 1.7 | 0.5 | 2.9 | 1.4 | 0.7 | **18.3** |
| *Laccaria* | OTU 27 | 0.5 | 0.9 | 0.3 | 0.6 | 1.3 | 0.3 | | **8.3** |
| *Cortinarius* | OTU 44 | 0.0 | 0.0 | 0.1 | 0.8 | 0.4 | 0.1 | 1.1 | |
| *Cryptococcus* | OTU 46 | 0.0 | 0.0 | 0.0 | 23.6 | 0.0 | 0.0 | **30.9** | |
| ***Glomeromycota*** | | | | | | | | | |
| *Glomeromycetes* | OTU 25 | 0.0 | 0.8 | 0.4 | 3.4 | 0.3 | 0.2 | 4.5 | |
| ***Rozellomycota*** | | | | | | | | | |
| | OTU 29 | 0.0 | 0.4 | 1.1 | 2.2 | 0.1 | 1.5 | 2.9 | |
| | OTU 38 | 0.8 | 1.2 | 0.7 | 2.0 | 0.5 | 0.3 | 2.6 | |
| | OTU 153 | 0.0 | 0.0 | 0.0 | 2.3 | 0.1 | 0.0 | 3.0 | |
| ***Zygomycota*** | | | | | | | | | |
| *Mortierella* | OTU 5 | 2.8 | 5.0 | 4.6 | 5.1 | 5.7 | 5.8 | **6.7** | **35.7** |
| *Mortierella* | OTU 33 | 0.0 | 0.6 | 1.3 | 1.1 | 0.7 | 1.0 | 1.4 | 4.3 |
| *Mortierella* | OTU 53 | 0.0 | 0.5 | 0.3 | 0.0 | 0.6 | 0.1 | | 3.9 |
| *Mortierella* | OTU 112 | 0.0 | 0.8 | 0.6 | 0.4 | 2.4 | 0.4 | 0.5 | **14.8** |
| Percentage of labeled taxa to total fraction [%] | | | | | **77** | **16** | | | |

[a] Taxonomic affiliation is based on the dynamic UNITE database (v7, release 01.08.2015) and was done with a bayesian classifier implied with MOTUHR based on the best hit of consensus taxonomy after 100 bootstrapped assignments (for further reference sequences based on 'massBLASTer' of UNITE see Table S12)

**Table S5. Taxonomic affiliation of fungal phylotypes (ITS gene sequences clustered at species-level 97% similarity cut-off) in numerical order.** Listed are reference sequences of the UNITE database (https://unite.ut.ee/analysis.php) and their phylogenetic affiliation based on the dynamic UNITE database (v7 release 1.08.2015) using MOTHUR.

| OTU | Reference sequence[a] | | | | | Taxonomy[b] | | | | |
|---|---|---|---|---|---|---|---|---|---|---|
| | | Accession[c] | SH code[d] | Q[e] | Id[f] | Phyl[g] | Class | Order | Family | Genus |
| **1** | *Trichosporon porosum* | NR_073209 | SH196641.07FU | 100 | 100 | *Baso* | *Tremellomycetes* | *Trichosporonales* | *Trichosporonaceae* | *Trichosporon* |
| **2** | *Saccharomycetales* | FN610974 | SH180919.07FU | 100 | 100 | *Asco* | *Saccharomycetes* | NA | NA | NA |
| **4** | *Mortierella humilis* | KP772755 | | 100 | 100 | *Zygo* | *Incertae sedis* | *Mortierellales* | *Mortierellaceae* | *Mortierella* |
| **5** | *Mortierella* | JQ312759 | | 99 | 98 | *Zygo* | *Incertae sedis* | *Mortierellales* | *Mortierellaceae* | *Mortierella* |
| **6** | *Cryptococcus terricola* | NR_073221 | SH190017.07FU | 100 | 100 | *Baso* | *Tremellomycetes* | *Tremellales* | *Incertae sedis* | *Cryptococcus* |
| **7** | *Ganoderma applanatum* | KJ857258 | SH187220.07FU | 100 | 100 | *Baso* | *Agaricomycetes* | *Polyporales* | *Ganodermataceae* | *Ganoderma* |
| **8** | *Trichoderma viride* | X93980 | SH187755.07FU | 100 | 100 | *Asco* | *Sordariomycetes* | *Hypocreales* | *Hypocreaceae* | *Trichoderma* |
| **9** | *Oidiodendron* | HM488481 | SH216993.07FU | 100 | 100 | *Asco* | *Leotiomycetes* | *Incertae sedis* | *Myxotrichaceae* | *Oidiodendron* |
| **10** | *Penicillium* | FJ379809 | SH182493.07FU | 100 | 100 | *Asco* | *Eurotiomycetes* | *Eurotiales* | *Trichocomaceae* | *Penicillium* |
| **11** | *Tomentella radiosa* | UDB017828 | SH184517.07FU | 100 | 100 | *Baso* | *Agaricomycetes* | *Thelephorales* | *Thelephoraceae* | *Tomentella* |
| **12** | *Hyaloriaceae* | HQ021773 | SH021770.07FU | 97 | 96 | *Baso* | *Agaricomycetes* | NA | NA | NA |
| **13** | *Cryptococcus podzolicus* | NR_073213 | SH181879.07FU | 100 | 100 | *Baso* | *Tremellomycetes* | *Tremellales* | *Incertae sedis* | *Cryptococcus* |
| **14** | *Paecilomyces* | LN886697 | | 98 | 97 | *Asco* | *Eurotiomycetes* | *Eurotiales* | *Trichocomaceae* | *Paecilomyces* |
| **15** | *Leucosporidiales* | FN565254 | SH193764.07FU | 100 | 100 | *Baso* | *Microbotryomycetes* | *Leucosporidiales* | NA | NA |
| **16** | *Tolypocladium cylindrosporum* | KJ028796 | SH184933.07FU | 100 | 99 | *Asco* | *Sordariomycetes* | *Hypocreales* | *Ophiocordycipitaceae* | *Elaphocordyceps* |
| **17** | *Oidiodendron* | KF212286 | SH217009.07FU | 98 | 98 | *Asco* | *Leotiomycetes* | *Incertae sedis* | *Myxotrichaceae* | *Oidiodendron* |
| **18** | *Nectria ramulariae* | FR717232 | SH217194.07FU | 100 | 100 | *Asco* | *Sordariomycetes* | *Hypocreales* | *Nectriaceae* | *Nectria* |
| **19** | *Mortierellales* | GU366727 | SH026735.07FU | 100 | 100 | *Zygo* | *Incertae sedis* | *Mortierellales* | *Mortierellaceae* | *Mortierella* |
| **20** | *Elaphomyces* | HQ021974 | SH190236.07FU | 100 | 100 | *Asco* | *Eurotiomycetes* | *Eurotiales* | *Elaphomycetaceae* | *Elaphomyces* |
| **21** | *Dermateaceae* | HQ021920 | SH196224.07FU | 99 | 98 | *Asco* | *Leotiomycetes* | *Helotiales* | *Dermateaceae* | NA |

| OTU | Reference sequence[a] | | | | | Taxonomy[b] | | | | |
|---|---|---|---|---|---|---|---|---|---|---|
| | | Accession[c] | SH code[d] | Q[e] | Id[f] | Phyl[g] | Class | Order | Family | Genus |
| **22** | *Syzygospora* | HQ211619 | SH182180.07FU | 100 | 100 | *Baso* | *Tremellomycetes* | *Cystofilobasidiales* | *Incertae sedis* | *Syzygospora* |
| **23** | *Mortierella gamsii* | AJ878509 | SH193938.07FU | 100 | 100 | *Zygo* | *Incertae sedis* | *Mortierellales* | *Mortierellaceae* | *Mortierella* |
| **24** | *Bionectria* | GQ219910 | SH211202.07FU | 100 | 100 | *Asco* | *Sordariomycetes* | *Hypocreales* | *Bionectriaceae* | *Bionectria* |
| **25** | *Glomeromycetes* | JF300543 | SH462390.07FU | 98 | 96 | *Glom* | *Glomeromycetes* | NA | NA | NA |
| **26** | *Glomeromycetes* | JF300543 | SH462390.07FU | 97 | 95 | *Glom* | *Glomeromycetes* | NA | NA | NA |
| **27** | *Laccaria amethystina* | HM189774 | SH220959.07FU | 100 | 100 | *Baso* | *Agaricomycetes* | *Agaricales* | *Hydnangiaceae* | *Laccaria* |
| **28** | *Cryptococcus podzolicus* | DQ069015 | SH181879.07FU | 97 | 96 | *Baso* | *Tremellomycetes* | *Tremellales* | *Incertae sedis* | *Cryptococcus* |
| **29** | *Rozellomycota* | AF504877 | SH211778.07FU | 100 | 99 | *Roz* | NA | NA | NA | NA |
| **30** | *Trechispora* | FJ820706 | SH009022.07FU | 99 | 99 | *Baso* | *Agaricomycetes* | *Trechisporales* | *Hydnodontaceae* | *Trechispora* |
| **31** | *Inocybe napipes* | AM882927 | SH196058.07FU | 100 | 100 | *Baso* | *Agaricomycetes* | *Agaricales* | *Inocybaceae* | *Inocybe* |
| **32** | *Oidiodendron* | FJ475564 | SH216991.07FU | 99 | 99 | *Asco* | *Leotiomycetes* | *Incertae sedis* | *Myxotrichaceae* | *Oidiodendron* |
| **33** | *Mortierellaceae* | JQ666581 | SH208762.07FU | 100 | 100 | *Zygo* | *Incertae sedis* | *Mortierellales* | *Mortierellaceae* | *Mortierella* |
| **35** | *Volutella* | HM136667 | SH474556.07FU | 100 | 100 | *Asco* | *Sordariomycetes* | *Hypocreales* | *Nectriaceae* | *Volutella* |
| **37** | *Mycena cinerella* | UDB021984 | SH220720.07FU | 100 | 100 | *Baso* | *Agaricomycetes* | *Agaricales* | *Mycenaceae* | *Mycena* |
| **38** | *Rozellomycota* | JQ666464 | SH001662.07FU | 99 | 99 | *Roz* | NA | NA | NA | NA |
| **39** | *Leptodontidium trabinellum* | GU934588 | SH019513.07FU | 100 | 100 | *Asco* | *Leotiomycetes* | *Helotiales* | *Incertae sedis* | *Leptodontidium* |
| **40** | *Chaunopycnis* | GQ302677 | SH184950.07FU | 99 | 98 | *Asco* | *Sordariomycetes* | *Hypocreales* | *Ophiocordycipitaceae* | *Chaunopycnis* |
| **41** | *Bulgaria inquinans* | JN033386 | SH194396.07FU | 100 | 100 | *Asco* | *Leotiomycetes* | *Leotiales* | *Bulgariaceae* | *Bulgaria* |
| **42** | *Mortierella simplex* | JX975982 | SH189943.07FU | 99 | 98 | *Zygo* | *Incertae sedis* | *Mortierellales* | *Mortierellaceae* | *Mortierella* |
| **44** | *Cortinarius diasemospermus* | UDB000066 | SH188559.07FU | 100 | 100 | *Baso* | *Agaricomycetes* | *Agaricales* | *Cortinariaceae* | *Cortinarius* |
| **45** | *Fungi* | KF297116 | SH032387.07FU | 98 | 95 | *Baso* | NA | NA | NA | NA |
| **46** | *Cryptococcus oeirensis* | NR_077106 | SH197623.07FU | 100 | 100 | *Baso* | *Tremellomycetes* | *Tremellales* | *Incertae sedis* | *Cryptococcus* |
| **48** | *Pochonia* | HM439557 | SH012122.07FU | 100 | 100 | *Asco* | *Sordariomycetes* | *Hypocreales* | *Clavicipitaceae* | NA |

| OTU | Reference sequence[a] | | | | | Taxonomy[b] | | | | |
|---|---|---|---|---|---|---|---|---|---|---|
| | | Accession[c] | SH code[d] | Q[e] | Id[f] | Phyl[g] | Class | Order | Family | Genus |
| **49** | *Tomentella* | KM576633 | | 100 | 100 | *Baso* | *Agaricomycetes* | *Thelephorales* | *Thelephoraceae* | *Tomentella* |
| **50** | *Mortierella longigemmata* | JX976055 | SH180129.07FU | 99 | 99 | *Zygo* | *Incertae sedis* | *Mortierellales* | *Mortierellaceae* | *Mortierella* |
| **51** | *Penicillium daleae* | GU981583 | SH182497.07FU | 100 | 100 | *Asco* | *Eurotiomycetes* | *Eurotiales* | *Trichocomaceae* | *Penicillium* |
| **53** | *Mortierella macrocystis* | JQ272448 | SH187863.07FU | 100 | 100 | *Zygo* | *Incertae sedis* | *Mortierellales* | *Mortierellaceae* | *Mortierella* |
| **55** | *Geomyces auratus* | KF039895 | SH183331.07FU | 100 | 100 | *Asco* | *Leotiomycetes* | *Incertae sedis* | *Incertae sedis* | *Geomyces* |
| **56** | *Hyaloriaceae* | FJ475763 | SH207620.07FU | 100 | 100 | *Baso* | *Agaricomycetes* | NA | NA | NA |
| **57** | *Lactarius camphoratus* | KF432971 | SH220116.07FU | 100 | 100 | *Baso* | *Agaricomycetes* | *Russulales* | *Russulaceae* | *Lactarius* |
| **58** | *Pochonia bulbillosa* | JX535180 | SH192574.07FU | 100 | 100 | *Asco* | *Sordariomycetes* | *Hypocreales* | *Clavicipitaceae* | *Pochonia* |
| **59** | *Trichoderma hamatum* | X93975 | SH177682.07FU | 100 | 100 | *Asco* | *Sordariomycetes* | *Hypocreales* | *Hypocreaceae* | *Trichoderma* |
| **60** | *Humicola* | KF981440 | SH195345.07FU | 99 | 99 | *Asco* | *Sordariomycetes* | *Sordariales* | *Chaetomiaceae* | NA |
| **61** | *Mortierella* | KC009383 | | 98 | 96 | *Zygo* | *Incertae sedis* | *Mortierellales* | *Mortierellaceae* | *Mortierella* |
| **62** | *Hyaloscyphaceae* | AB476538 | SH196230.07FU | 98 | 97 | *Asco* | *Leotiomycetes* | *Helotiales* | NA | NA |
| **64** | *Pezizomycotina* | KF359573 | SH217842.07FU | 100 | 100 | *Asco* | *Eurotiomycetes* | *Chaetothyriales* | NA | NA |
| **66** | *Aspergillus versicolor* | LN482531 | SH186265.07FU | 100 | 100 | *Asco* | *Eurotiomycetes* | *Eurotiales* | *Trichocomaceae* | *Aspergillus* |
| **67** | *Russula cyanoxantha* | KF432956 | SH186707.07FU | 100 | 100 | *Baso* | *Agaricomycetes* | *Russulales* | *Russulaceae* | *Russula* |
| **69** | *Oidiodendron* | JX456904 | SH216990.07FU | 99 | 99 | *Asco* | *Leotiomycetes* | *Incertae sedis* | *Myxotrichaceae* | *Oidiodendron* |
| **71** | *Penicillium arianeae* | KC773833 | SH005531.07FU | 100 | 100 | *Asco* | *Eurotiomycetes* | *Eurotiales* | *Trichocomaceae* | NA |
| **72** | *Volutella* | JX507700 | SH217585.07FU | 100 | 100 | *Asco* | *Sordariomycetes* | *Hypocreales* | *Nectriaceae* | NA |
| **73** | *Trichosporon moniliiforme* | NR_073240 | SH196643.07FU | 100 | 100 | *Baso* | *Tremellomycetes* | *Trichosporonales* | *Trichosporonaceae* | *Trichosporon* |
| **77** | *Tremella fibulifera* | KP986518 | | 93 | 89 | *Baso* | NA | NA | NA | NA |
| **81** | *Luellia recondita* | JF519380 | SH179954.07FU | 100 | 100 | *Baso* | *Agaricomycetes* | *Trechisporales* | *Hydnodontaceae* | *Luellia* |
| **83** | *Chaetosphaeria myriocarpa* | AF178552 | SH198584.07FU | 97 | 97 | *Asco* | *Sordariomycetes* | *Chaetosphaeriales* | *Chaetosphaeriaceae* | *Chaetosphaeria* |
| **85** | *Microbotryomycetes* | HM036653 | SH180664.07FU | 99 | 99 | *Baso* | *Microbotryomycetes* | NA | NA | NA |

| OTU | Reference sequence[a] | | | | | Taxonomy[b] | | | | |
|---|---|---|---|---|---|---|---|---|---|---|
| | | Accession[c] | SH code[d] | Q[e] | Id[f] | Phyl[g] | Class | Order | Family | Genus |
| **86** | *Cladosporium cladosporioides* | LN482458 | SH216250.07FU | 100 | 100 | *Asco* | *Dothideomycetes* | *Capnodiales* | *Davidiellaceae* | NA |
| **88** | *Cladophialophora chaetospira* | KF359558 | SH213266.07FU | 97 | 97 | *Asco* | *Eurotiomycetes* | *Chaetothyriales* | *Herpotrichiellaceae* | *Cladophialophora* |
| **91** | *Exophiala* | HM439556 | SH197643.07FU | 100 | 100 | *Asco* | *Eurotiomycetes* | *Chaetothyriales* | *Herpotrichiellaceae* | *Exophiala* |
| **94** | *Helotiales* | HQ021923 | SH201613.07FU | 99 | 99 | *Asco* | *Leotiomycetes* | *Helotiales* | NA | NA |
| **96** | *Bionectria ochroleuca* | JX967106 | SH182678.07FU | 100 | 100 | *Asco* | *Sordariomycetes* | *Hypocreales* | *Bionectriaceae* | *NA* |
| **98** | *Fungi* | KT194869 | | 99 | 98 | NA | NA | NA | NA | NA |
| **103** | *Trechispora araneosa* | AF347084 | | 99 | 99 | *Baso* | *Agaricomycetes* | *Trechisporales* | *Hydnodontaceae* | *Trechispora* |
| **108** | *Chytridiomycota* | EF619656 | SH462789.07FU | 98 | 95 | *NA* | NA | NA | NA | NA |
| **111** | *Capronia fungicola* | AF050246 | SH015749.07FU | 97 | 94 | *Asco* | *Eurotiomycetes* | *Chaetothyriales* | *Herpotrichiellaceae* | *NA* |
| **112** | *Mortierella gemmifera* | JX976121 | SH185196.07FU | 100 | 100 | *Zygo* | *Incertae sedis* | *Mortierellales* | *Mortierellaceae* | *Mortierella* |
| **113** | *Capnodiales* | KF617528 | SH472617.07FU | 98 | 98 | *Asco* | NA | NA | NA | NA |
| **114** | *Fungi* | JX675128 | SH216421.07FU | 95 | 93 | *Chyt* | NA | NA | NA | NA |
| **121** | *Saccharomycetales* | FN610974 | SH180919.07FU | 97 | 95 | *Asco* | *Saccharomycetes* | NA | NA | NA |
| **128** | *Helotiales* | HQ850140 | SH215245.07FU | 98 | 97 | *Asco* | *Leotiomycetes* | NA | NA | NA |
| **130** | *Lycoperdon pyriforme* | DQ112557 | SH175879.07FU | 100 | 100 | *Baso* | *Agaricomycetes* | *Agaricales* | *Agaricaceae* | *Lycoperdon* |
| **131** | *Lactarius quietus* | UDB015797 | SH220118.07FU | 100 | 100 | *Baso* | *Agaricomycetes* | *Russulales* | *Russulaceae* | *Lactarius* |
| **135** | *Helotiales* | AY969487 | SH195228.07FU | 100 | 100 | *Asco* | *Leotiomycetes* | *Helotiales* | *Hyaloscyphaceae* | *NA* |
| **138** | *Dermateaceae* | FN610995 | SH014516.07FU | 99 | 99 | *Asco* | *Leotiomycetes* | *Helotiales* | *NA* | *NA* |
| **140** | *Exophiala moniliae* | HE605213 | SH199199.07FU | 100 | 100 | *Asco* | *Eurotiomycetes* | *Chaetothyriales* | *Herpotrichiellaceae* | *Exophiala* |
| **141** | *Rozellomycota* | HM069428 | SH025237.07FU | 99 | 99 | *Roz* | NA | NA | NA | NA |
| **153** | *Rozellomycota* | HM069429 | SH025236.07FU | 98 | 97 | *Roz* | NA | NA | NA | NA |
| **154** | *Rozellomycota* | JQ666484 | SH020528.07FU | 98 | 96 | *Roz* | NA | NA | NA | NA |
| **157** | *Helotiales* | AY394669 | SH025727.07FU | 95 | 95 | *Asco* | *Leotiomycetes* | NA | NA | NA |

| OTU | Reference sequence[a] | | | | | Taxonomy[b] | | | | |
|---|---|---|---|---|---|---|---|---|---|---|
| | | Accession[c] | SH code[d] | Q[e] | Id[f] | Phyl[g] | Class | Order | Family | Genus |
| **161** | *Crocicreas* | AY969794 | | 99 | 99 | *Asco* | *Leotiomycetes* | *Helotiales* | *Helotiaceae* | *Crocicreas* |
| **177** | *Ilyonectria crassa* | KJ475469 | SH202967.07FU | 100 | 100 | *Asco* | *Sordariomycetes* | *Hypocreales* | *Incertae sedis* | *Ilyonectria* |
| **185** | *Pleosporales* | GU083188 | SH200451.07FU | 98 | 96 | *Asco* | *Dothideomycetes* | *Pleosporales* | NA | NA |
| **187** | *Cenococcum geophilum* | AM087244 | SH199613.07FU | 100 | 100 | *Asco* | *Dothideomycetes* | *Hysteriales* | *Gloniaceae* | *Cenococcum* |
| **188** | *Penicillium corylophilum* | KF170363 | SH207150.07FU | 99 | 99 | *Asco* | *Eurotiomycetes* | *Eurotiales* | *Trichocomaceae* | *Penicillium* |
| **190** | *Glomeromycetes* | JF300543 | SH462390.07FU | 90 | 82 | *Glom* | *Glomeromycetes* | NA | NA | NA |
| **198** | *Cladophialophora* | HQ211891 | SH193233.07FU | 97 | 95 | *Asco* | *Eurotiomycetes* | *Chaetothyriales* | *Herpotrichiellaceae* | *Cladophialophora* |
| **199** | *Xenasmatella* | JQ272394 | SH026877.07FU | 100 | 100 | *Baso* | *Agaricomycetes* | *Polyporales* | *Incertae sedis* | *Phlebiella* |
| **256** | *Hypocreales* | JF449629 | SH203174.07FU | 100 | 100 | *Asco* | *Sordariomycetes* | *Hypocreales* | NA | NA |
| **265** | *Helotiales* | HM044632 | SH013010.07FU | 95 | 95 | *Asco* | *Leotiomycetes* | *Helotiales* | NA | NA |
| **302** | *Mortierella parvispora* | AB476418 | SH193938.07FU | 99 | 99 | *Zygo* | *Incertae sedis* | *Mortierellales* | *Mortierellaceae* | *Mortierella* |
| **304** | *Rhodotorula diffluens* | NR_073289 | SH213958.07FU | 94 | 90 | *Baso* | *Microbotryomycetes* | *Sporidiobolales* | *Incertae sedis* | NA |
| **350** | *Mucor* | JQ676211 | SH188380.07FU | 100 | 100 | *Zygo* | *Incertae sedis* | *Mucorales* | *Mucoraceae* | *Mucor* |

[a] Reference sequences based on ‘massBLASTer’ of the UNITE database including sequences from the International Nucleotide Sequence Database (GenBank, EMBL, DDBJ) and environmental samples (isolated from soil, ectomycorrhizal roots, orchids etc.) in UNITE.
[b] Taxonomy is based on the dynamic UNITE database (v7, release 01.08.2015) and was done with a bayesian classifier implied with MOTHUR based on the best hit of consensus taxonomy after 100 bootstrapped assignments (implementation of Wang *et al.* 2007). ‘NA’ (not applicable) indicates no further affiliation possible based on consensus taxonomy.
[c] Accession number of reference sequence applicable in International Nucleotide Sequence Database.
[d] SH code provided by UNITE considering species hypothesis (comprise any species level group of individuals sharing a given set of observed characteristics).
[e] Query [%] of the BLAST alignment calculated with the number of mismatches over a 355 nt long input sequence and the corresponding section of the reference sequence.
[f] Maximum sequence identity [%] over the BLAST alignment.
[g] Fungal phyla using the following abbreviations: ‘Asco’, *Ascomycota*; ‘Baso’, *Basidiomycota*; ‘Chyt’, *Chytridiomycota*; ‘Glom’, *Glomeromycota*; ‘Roz’, *Rozellomycota*; ‘Zygo’, *Zygomycota*.

**Table S6. Relative abundances of labeled bacterial taxa (OTU) based on 16S rRNA gene sequences in all fractions (H, heavy; M, middle; L, light) of [$^{12}C$]- and [$^{13}C_1$]-methanol treatment at pH 4 of pH SIP experiment.** Black faces indicate values that were used for calculating 'labeling proportions' as indicators of relative importance. Bold faces indicate 'labeling proportions' > 5 %.

| Taxonomic affiliation [a] | Labeled OTU | Relative abundance [%] | | | | | | Labeling proportion [%] | |
|---|---|---|---|---|---|---|---|---|---|
| | | $^{12}C$ | | | $^{13}C$ | | | | |
| | | H | M | L | H | M | L | H | M |
| ***Actinobacteria*** | | | | | | | | | |
| *Leifsonia* [d] | OTU 721 | 0.4 | 0.54 | 2.5 | 10.8 | 7.0 | 1.2 | **45.0** | **13.9** |
| ***Bacteroidetes*** | | | | | | | | | |
| *Chitinophaga* [d] | OTU 1020 | 3.5 | 11.5 | 1.7 | 5.3 | 9.2 | 2.1 | **22.3** | |
| *Sphingobacteriales* [b] | OTU 1098 | 0.0 | 0.5 | 0.5 | 0.5 | 0.1 | 0.2 | 2.2 | |
| ***Firmicutes*** | | | | | | | | | |
| *Paenibacillus* [d] | OTU 206 | 0.2 | 0.4 | 0.8 | 0.6 | 0.2 | 0.0 | 2.6 | |
| ***Alphaproteobacteria*** | | | | | | | | | |
| *Methylovirgula* [d] | OTU 438 | 1.0 | 0.4 | 3.1 | 6.7 | 42.3 | 3.5 | **27.9** | **83.5** |
| *Sphingomonas* [d] | OTU 449 | 0.2 | 0.0 | 1.7 | 0.7 | 0.6 | 3.0 | | 1.2 |
| ***Gammaproteobacteria*** | | | | | | | | | |
| *Rhodanobacter* [d,e] | OTU 343 | 0.2 | 0.0 | 0.2 | 0.1 | 0.7 | 0.7 | | 1.4 |
| Percentage of labeled taxa to total fraction [%] | | | | | **24** | **51** | | | |

[a] Taxonomic affiliation was done with BLASTn (November 2015) and is based on the next cultivated hit for each OTU (for further information see Table S8)
[b] Sequence identity of next cultured hit < 90 %, phylogenetic affiliation up to order level
[d] Sequence identity of next cultured hit ≥ 95 %, phylogenetic affiliation up to genus level
[e] Query of next cultured hit was only 72 % with BLASTn analysis

**Table S7. Relative abundances of labeled bacterial taxa (OTU) based on 16S rRNA gene sequences in all fractions (H, heavy; M, middle; L, light) of [$^{12}$C]- and [$^{13}C_1$]-methanol treatment at pH 7 of pH SIP experiment.** Black faces indicate values that were used for calculating 'labeling proportions' as indicators of relative importance. Bold faces indicate 'labeling proportions' > 5 %.

| Taxonomic affiliation [a] | Labeled OTU | Relative abundance [%] $^{12}$C H | $^{12}$C M | $^{12}$C L | $^{13}$C H | $^{13}$C M | $^{13}$C L | Labeling proportion [%] H | Labeling proportion [%] M |
|---|---|---|---|---|---|---|---|---|---|
| ***Actinobacteria*** | | | | | | | | | |
| *Leifsonia* [d] | OTU 721 | 1.0 | 1.7 | 2.7 | 8.5 | 9.3 | 2.9 | **19.7** | **19.4** |
| ***Bacteroidetes*** | | | | | | | | | |
| *Chryseobacterium* [d] | OTU 1045 | 8.5 | 11.6 | 5.0 | 32.5 | 23.1 | 23.5 | **75.1** | **48.1** |
| *Sphingobacterium* [d] | OTU 1078 | 1.7 | 0.4 | 0.5 | 0.9 | 1.0 | 0.8 | | 2.1 |
| *Sphingobacteriaceaea* [c] | OTU 1094 | 0.3 | 0.6 | 0.5 | 0.5 | 0.6 | 0.1 | 1.3 | |
| *Cytophaga* [d] | OTU 1108 | 0.1 | 0.4 | 0.4 | 0.3 | 0.5 | 0.1 | | 1.1 |
| ***Alphaproteobacteria*** | | | | | | | | | |
| *Caulobacter* [d] | OTU 431 | 0.8 | 0.3 | 1.6 | 1.0 | 0.1 | 0.6 | 2.3 | |
| *Paracoccus* [d] | OTU 450 | 0.0 | 0.0 | 0.0 | 0.1 | 0.8 | 0.0 | | 1.6 |
| ***Betaproteobacteria*** | | | | | | | | | |
| *Methylophilus* [d] | OTU 358 | 0.6 | 0.2 | 0.5 | 0.4 | 9.8 | 0.0 | | **20.4** |
| ***Verrucomicrobia*** | | | | | | | | | |
| *Terrimicrobium* [d] | OTU 54 | 0.3 | 0.1 | 0.6 | 0.7 | 3.5 | 0.0 | 1.7 | 7.2 |
| Percentage of labeled taxa to total fraction [%] | | | | | **43** | **48** | | | |

[a] Taxonomic affiliation was done with BLASTn (November 2015) and is based on the next cultivated hit for each OTU (for further information see Table S8)
[c] Sequence identity of next cultured hit < 95 %, phylogenetic affiliation up to family level
[d] Sequence identity of next cultured hit ≥ 95 %, phylogenetic affiliation up to genus level

**Table S8. Relative abundances of labeled taxa (OTU) based on *mxaF* gene sequences in all fractions (H, heavy; M, middle; L, light) of [$^{12}$C]- and [$^{13}C_1$]-methanol treatments at pH 4 of pH SIP experiment.** Black faces indicate values that were used for calculating 'labeling proportions' as indicators of relative importance. Bold faces indicate 'labeling proportions' > 5 %.

| Taxonomic affiliation [a] | Labeled OTU | Relative abundance [%] | | | | | | Labeling proportion [%] | |
|---|---|---|---|---|---|---|---|---|---|
| | | $^{12}$C | | | $^{13}$C | | | | |
| | | H | M | L | H | M | L | H | M |
| ***Methylobacteriaceae*** | | | | | | | | | |
| | OTU 7 | 0.0 | 0.1 | 0.0 | 0.0 | 1.1 | 0.0 | | 1.3 |
| | OTU 55 | 1.1 | 1.3 | 0.9 | 2.1 | 0.1 | 0.7 | 2.7 | |
| *ambiguous* [b] | | | | | | | | | |
| | OTU 0 | 0.0 | 0.0 | 0.2 | 0.2 | 1.9 | 0.1 | | 2.2 |
| ***Hyphomicrobiaceae*** | | | | | | | | | |
| | OTU 185 | 0.6 | 0.4 | 1.9 | 9.9 | 3.7 | 7.4 | **12.2** | 4.3 |
| | OTU 186 | 0.0 | 0.0 | 0.1 | 0.7 | 0.2 | 0.4 | 0.9 | |
| | OTU 243 | 0.0 | 0.0 | 1.6 | 0.2 | 11.7 | 0.1 | | **13.7** |
| | OTU 257 | 0.3 | 0.5 | 2.0 | 7.4 | 0.2 | 0.8 | **9.1** | |
| | OTU 308 | 0.0 | 0.0 | 0.6 | 0.5 | 0.0 | 0.7 | 0.7 | |
| | OTU 309 | 0.1 | 0.0 | 0.5 | 5.3 | 0.0 | 0.1 | **6.5** | |
| *ambiguous* [b] | | | | | | | | | |
| | OTU 172 | 0.1 | 0.2 | 8.2 | 8.7 | 0.0 | 2.8 | **10.8** | |
| | OTU 210 | 0.1 | 2.6 | 6.2 | 3.0 | 19.9 | 5.5 | 3.7 | **23.1** |
| | OTU 214 | 0.5 | 0.2 | 2.2 | 3.5 | 1.6 | 3.2 | 4.4 | 1.9 |
| | OTU 236 | 1.6 | 19.5 | 46.0 | 22.1 | 31.7 | 25.4 | **27.3** | **36.9** |
| | OTU 266 | 0.4 | 0.0 | 1.4 | 1.4 | 0.1 | 0.3 | 1.7 | |
| | OTU 310 | 0.0 | 0.1 | 0.2 | 0.1 | 0.5 | 0.3 | | 0.6 |
| ***Methylorhabdus*** *ambiguous* [b] | | | | | | | | | |
| | OTU 18 | 0.0 | 0.8 | 2.4 | 8.0 | 8.2 | 0.0 | **10.0** | **9.6** |
| ***Beijerinckiaceae*** | | | | | | | | | |
| | OTU 144 | 0.0 | 0.0 | 1.3 | 1.0 | 0.0 | 0.2 | 1.2 | |
| *ambiguous* [b] | | | | | | | | | |
| | OTU 337 | 0.0 | 0.0 | 0.1 | 1.5 | 0.1 | 0.0 | 1.8 | |
| | OTU 338 | 0.1 | 0.1 | 0.3 | 4.1 | 0.0 | 0.3 | **5.1** | |
| | OTU 340 | 0.1 | 0.7 | 2.3 | 1.5 | 3.8 | 0.6 | 1.9 | 4.5 |
| | OTU 349 | 0.1 | 0.6 | 2.9 | 0.5 | 1.7 | 1.4 | | 2.0 |
| Percentage of labeled taxa to total fraction [%] | | | | | **81** | **86** | | | |

[a] Taxonomic affiliation was done with BLASTn (December 2015; for further information see Table S2) and confirmed by positioning in phylogenetic tree (data not shown)

[b] Sequence identity with BLASTn < 90 % as well as ambiguous position in phylogenetic tree (for further information see S2)

**Table S9. Relative abundances of labeled taxa (OTU) based on *mxaF* gene sequences in all fractions (H, heavy; M, middle; L, light) of [$^{12}$C]- and [$^{13}C_u$]-methanol treatments at pH 7 of pH SIP experiment.** Black faces indicate values that were used for calculating 'labeling proportions' as indicators of relative importance. Bold faces indicate 'labeling proportions' > 5 %.

| Taxonomic affiliation [a] | Labeled OTU | Relative abundance [%] | | | | | | Labeling proportion [%] | |
|---|---|---|---|---|---|---|---|---|---|
| | | $^{12}$C | | | $^{13}$C | | | | |
| | | H | M | L | H | M | L | H | M |
| ***Methylobacteriaceae*** | | | | | | | | | |
| | OTU 55 | 27.0 | 37.4 | 25.8 | 72.0 | 39.7 | 9.0 | **95.4** | **80.1** |
| | OTU 107 | 0.1 | 0.2 | 0.4 | 0.1 | 0.6 | 0.0 | | 1.2 |
| *ambiguous* [b] | | | | | | | | | |
| | OTU 141 | 0.0 | 0.7 | 1.0 | 3.5 | 1.3 | 0.4 | 4.6 | 2.7 |
| ***Hyphomicrobiaceae*** | | | | | | | | | |
| | OTU 185 | 1.4 | 1.9 | 5.8 | 0.9 | 7.3 | 12.7 | | **14.7** |
| | OTU 213 | 0.0 | 0.3 | 0.4 | 0.0 | 0.7 | 0.2 | | 1.3 |
| Percentage of labeled taxa to total fraction [%] | | | | | **75** | **50** | | | |

[a] Taxonomic affiliation was done with BLASTn (December 2015; for further information see Table S2) and confirmed by positioning in phylogenetic tree (data not shown)

[b] Sequence identity with BLASTn < 90 % as well as ambiguous position in phylogenetic tree (for further information see Table S2)

**Table S10. Relative abundances of labeled fungal taxa (OTU) based on ITS gene sequences in all fractions (H, heavy; M, middle; L, light) of [$^{12}$C]- and [$^{13}C_1$]-methanol treatments at pH 4 of pH SIP experiment.** Black faces indicate values that were used for calculating 'labeling proportions' as indicators of relative importance. Bold faces indicate 'labeling proportions' > 5 %.

| Taxonomic affiliation [a] | Labeled OTU | Relative abundance [%] $^{12}$C H | $^{12}$C M | $^{12}$C L | $^{13}$C H | $^{13}$C M | $^{13}$C L | Labeling proportion [%] H | M |
|---|---|---|---|---|---|---|---|---|---|
| ***Ascomycota*** | | | | | | | | | |
| *Bionectria* | OTU 24 | 1.3 | 1.0 | 0.6 | 1.8 | 0.8 | 1.4 | 3.8 | |
| *Chaunopycnis* | OTU 40 | 0.2 | 0.3 | 0.8 | 0.8 | 0.6 | 1.0 | 1.7 | |
| *Clavicipitaceae* | OTU 48 | 0.3 | 0.2 | 1.3 | 0.7 | 0.3 | 0.8 | 1.4 | |
| *Penicillium* | OTU 51 | 0.3 | 0.4 | 0.5 | 0.6 | 0.1 | 1.4 | 1.2 | |
| *Trichoderma* | OTU 59 | 0.3 | 0.0 | 1.0 | 0.7 | 0.5 | 1.3 | 1.4 | |
| *Chaetomiaceae* | OTU 60 | 0.6 | 0.4 | 1.9 | 1.3 | 1.2 | 3.1 | 2.7 | **6.9** |
| *Trichocomaceae* | OTU 71 | 0.0 | 0.3 | 0.2 | 0.1 | 0.6 | 0.8 | | 3.3 |
| *Ascomycota* | OTU 113 | 0.4 | 0.6 | 0.6 | 0.6 | 0.4 | 0.1 | 1.3 | |
| *Helotiales* | OTU 265 | 0.0 | 0.0 | 0.0 | 0.0 | 0.8 | 0.0 | | 4.5 |
| ***Basidiomycota*** | | | | | | | | | |
| *Ganoderma* | OTU 7 | 2.0 | 2.7 | 3.4 | 2.9 | 3.3 | 2.0 | **5.9** | **19.5** |
| *Tomentella* | OTU 11 | 0.1 | 0.3 | 0.5 | 0.4 | 0.6 | 0.0 | | 3.7 |
| *Agaricomycetes* | OTU 12 | 0.5 | 0.5 | 1.0 | 2.4 | 1.5 | 1.1 | **5.1** | **8.5** |
| *Cryptococcus* | OTU 13 | 1.3 | 0.8 | 1.2 | 1.9 | 1.2 | 0.6 | 3.9 | **6.9** |
| *Leucosporidiales* | OTU 15 | 1.1 | 3.0 | 1.4 | 3.9 | 2.8 | 1.5 | **8.1** | |
| *Laccaria* | OTU 27 | 0.7 | 1.4 | 0.8 | 1.0 | 1.0 | 0.6 | 2.0 | |
| *Trechispora* | OTU 30 | 1.6 | 2.0 | 2.1 | 2.9 | 1.8 | 2.0 | **5.9** | |
| *Mycena* | OTU 37 | 0.1 | 0.1 | 0.0 | 0.7 | 0.1 | 0.1 | 1.4 | |
| *Basidiomycota* | OTU 45 | 0.5 | 1.0 | 0.1 | 0.8 | 0.1 | 0.5 | 1.7 | |
| *Microbotryomycetes* | OTU 85 | 0.0 | 0.2 | 0.3 | 0.6 | 0.2 | 0.1 | 1.2 | |
| *Lycoperdon* | OTU 130 | 0.2 | 0.1 | 0.3 | 0.6 | 0.6 | 0.4 | 1.2 | 3.3 |
| *Phlebiella* | OTU 199 | 0.1 | 0.1 | 0.1 | 0.6 | 0.4 | 0.1 | 1.2 | |
| ***Rozellomycota*** | OTU 38 | *0.6* | *1.2* | *0.3* | *1.6* | *1.0* | *0.6* | *3.3* | |
| ***Zygomycota*** | | | | | | | | | |
| *Mortierella* | OTU 4 | 6.5 | 11.8 | 9.5 | 14.8 | 11.7 | 6.7 | **30.6** | |
| *Mortierella* | OTU 5 | 3.4 | 3.2 | 2.3 | 3.6 | 3.9 | 1.8 | **7.5** | **22.8** |
| *Mortierella* | OTU 19 | 0.7 | 0.9 | 1.0 | 1.6 | 1.4 | 1.2 | 3.3 | **8.1** |
| *Mortierella* | OTU 23 | 0.5 | 0.1 | 0.1 | 0.8 | 0.3 | 0.2 | 1.6 | |
| *Mortierella* | OTU 33 | 0.4 | 0.5 | 0.1 | 1.3 | 1.1 | 0.7 | 2.6 | **6.5** |
| *Mortierella* | OTU 50 | 0.6 | 0.5 | 0.5 | 0.6 | 1.0 | 0.2 | | **6.1** |
| Percentage of labeled taxa to total fraction [%] | | | | | **48** | **17** | | | |

[a] Taxonomic affiliation is based on the dynamic UNITE database (v7, release 01.08.2015) and was done with a bayesian classifier implied with MOTUHR based on the best hit of consensus taxonomy after 100 bootstrapped assignments (for further reference sequences based on ‘massBLASTer’ of UNITE see Table S5)

**Table S11. Relative abundances of labeled fungal taxa (OTU) based on ITS gene sequences in all fractions (H, heavy; M, middle; L, light) of [$^{12}$C]- and [$^{13}C_1$]-methanol treatments at pH 7 of pH SIP experiment.** Black faces indicate values that were used for calculating 'labeling proportions' as indicators of relative importance. Bold faces indicate 'labeling proportions' > 5 %.

| | | Relative abundance [%] | | | | | | Labeling proportion [%] | |
|---|---|---|---|---|---|---|---|---|---|
| | | $^{12}$C | | | $^{13}$C | | | | |
| **Taxonomic affiliation [a]** | **Labeled OTU** | **H** | **M** | **L** | **H** | **M** | **L** | **H** | **M** |
| ***Ascomycota*** | | | | | | | | | |
| *Saccharomycetes* | OTU 2 | 1.3 | 0.6 | 0.3 | 1.9 | 0.4 | 0.2 | 3.0 | |
| *Paecilomyces* | OTU 14 | 1.6 | 1.0 | 1.0 | 2.7 | 6.6 | 4.8 | 4.2 | **13.5** |
| *Bionectria* | OTU 24 | 1.3 | 1.0 | 0.6 | 1.8 | 1.1 | 2.7 | 2.8 | |
| *Chaetothyriales* | OTU 64 | 0.0 | 0.1 | 0.0 | 0.1 | 0.6 | 0.0 | | 1.3 |
| ***Basidiomycota*** | | | | | | | | | |
| *Trichosporon* | OTU 1 | 32.4 | 29.9 | 21.0 | 46.4 | 34.7 | 20.1 | **72.7** | **71.0** |
| *Cryptococcus* | OTU 6 | 0.9 | 0.9 | 0.2 | 2.3 | 1.6 | 1.5 | 3.6 | 3.3 |
| *Ganoderma* | OTU 7 | 2.0 | 2.7 | 3.4 | 2.1 | 3.5 | 1.3 | | **7.3** |
| *Leucosporidiales* | OTU 15 | 1.1 | 3.0 | 1.4 | 2.0 | 2.2 | 0.2 | 3.1 | |
| *Syzygospora* | OTU 22 | 1.4 | 0.1 | 0.1 | 1.9 | 0.8 | 0.6 | 3.0 | 1.7 |
| *Trichosporon* | OTU 73 | 0.4 | 0.1 | 0.0 | 1.8 | 0.5 | 0.7 | 2.9 | |
| *Basidiomycota* | OTU 77 | 1.0 | 0.4 | 0.1 | 1.4 | 0.5 | 0.3 | 2.2 | |
| ***Rozellomycota*** | OTU 154 | 0.2 | 0.0 | 0.0 | 0.9 | 0.2 | 0.2 | 1.4 | |
| ***Zygomycota*** | | | | | | | | | |
| *Mortierella* | OTU 23 | 0.5 | 0.1 | 0.1 | 0.7 | 1.0 | 0.1 | 1.1 | 2.0 |
| Percentage of labeled taxa to total fraction [%] | | | | | **64** | **49** | | | |

[a] Taxonomic affiliation is based on the dynamic UNITE database (v7, release 01.08.2015) and was done with a bayesian classifier implied with MOTUHR based on the best hit of consensus taxonomy after 100 bootstrapped assignments (for further reference sequences based on 'massBLASTer' of UNITE see Table S5)

**Table S12. Relative abundance of fungal taxa based on ITS gene sequences from combined pyrosequencing data sets of [$^{12}$C]- and [$^{13}C_u$]-substrate treatments of both SIP experiments.** Only taxa with a relative abundance ≥ 0.5% are listed, abundance < 0.5% is indicated by ∗, no presence is indicated by -. Percentages are always related to filtered datasets of ITS gene sequence of pyrosequencing amplicon libraries.

| | **Substrate SIP experiment[c]** | | | | | | | | **pH shift SIP experiment[d]** | | | |
|---|---|---|---|---|---|---|---|---|---|---|---|---|
| | | | | | | | | | pH 4 | | pH 7 | |
| | **$t_0$** | **MeOH** | **Ace +** | **Glu +** | **Xyl +** | **Van +** | **$CO_2$ +** | **$CO_2$** | **$t_0$** | **MeOH** | **$t_0$** | **MeOH** |
| **number of sequences** combined data sets & singletons removed | 8619 | 8535 | 8670 | 8833 | 8860 | 8516 | 8647 | 8526 | 4301 | 8612 | 4251 | 8673 |
| **Taxonomic affiliation [a]** | **Relative abundance [%]** | | | | | | | | | | | |
| ***Ascomycota*** | 45.16 | 37.56 | 16.68 | 14.00 | 8.18 | 51.89 | 47.84 | 57.71 | 53.80 | 38.76 | 50.60 | 36.87 |
| ***Dothideomycetes*** | 1.13 | ∗ | ∗ | ∗ | ∗ | ∗ | 1.41 | 1.38 | 1.74 | ∗ | 1.11 | ∗ |
| ***Eurotiomycetes*** | 5.80 | 9.31 | 4.35 | 5.38 | 2.61 | 10.24 | 13.81 | 12.68 | 17.41 | 9.72 | 24.84 | 11.54 |
| *Chaetothyriales* | ∗ | 0.71 | 0.60 | 1.23 | ∗ | 4.20 | ∗ | ∗ | ∗ | 0.82 | ∗ | 0.65 |
| *Eurotiales* | 5.53 | 8.60 | 3.75 | 4.14 | 2.44 | 6.04 | 13.58 | 12.30 | 16.93 | 8.89 | 24.35 | 10.90 |
| *Trichocomaceae* | 3.03 | 8.54 | 3.70 | 4.11 | 2.42 | 5.86 | 12.83 | 11.93 | 14.76 | 8.69 | 13.67 | 10.80 |
| *Elaphomycetaceae* | 2.49 | ∗ | ∗ | ∗ | ∗ | ∗ | 0.75 | ∗ | 2.16 | ∗ | 10.63 | ∗ |
| ***Leotiomycetes*** | 10.85 | 10.03 | 4.86 | 2.94 | 2.00 | 11.16 | 12.41 | 18.21 | 10.72 | 8.19 | 8.52 | 7.11 |
| *Helotiales* | 2.84 | 5.21 | 1.30 | 1.31 | 0.63 | 5.28 | 7.49 | 7.95 | 2.53 | 2.04 | 2.35 | 2.08 |
| *Leotiales* | ∗ | ∗ | 1.23 | ∗ | ∗ | ∗ | ∗ | 2.50 | ∗ | ∗ | - | ∗ |
| *Incertae sedis* | 7.08 | 4.18 | 2.16 | 1.06 | 1.08 | 4.94 | 3.99 | 6.69 | 8.07 | 5.84 | 5.01 | 4.77 |
| ***Saccharomycetes*** | 18.49 | 7.05 | 2.70 | 2.34 | 0.80 | 6.11 | 6.27 | 7.86 | 1.86 | 0.60 | 2.49 | 0.90 |
| ***Sordariomycetes*** | 6.89 | 9.82 | 4.12 | 2.62 | 2.51 | 22.91 | 12.17 | 15.66 | 19.81 | 18.32 | 11.46 | 15.81 |
| *Chaetosphaeriales* | 0.68 | 1.65 | ∗ | ∗ | ∗ | 0.72 | 0.87 | 1.02 | 3.30 | 4.30 | 1.91 | 3.46 |
| *Hypocreales* | 5.70 | 7.44 | 3.44 | 1.64 | 2.12 | 21.54 | 10.48 | 13.65 | 15.02 | 11.83 | 7.60 | 10.73 |
| *Nectriaceae* | 0.70 | 1.07 | ∗ | ∗ | 0.51 | 9.63 | 2.73 | 2.78 | 1.05 | 1.45 | 0.73 | 1.25 |
| *Ophiocordycipitaceae* | ∗ | ∗ | 1.03 | ∗ | ∗ | 5.97 | 1.33 | 3.19 | 2.58 | 1.58 | 1.51 | 1.52 |

| | Substrate SIP experiment[c] | | | | | | | | pH shift SIP experiment[d] | | | |
|---|---|---|---|---|---|---|---|---|---|---|---|---|
| | | | | | | | | | pH 4 | | pH 7 | |
| | **$t_0$** | **MeOH** | **Ace +** | **Glu +** | **Xyl +** | **Van +** | **$CO_2$ +** | **$CO_2$** | **$t_0$** | **MeOH** | **$t_0$** | **MeOH** |
| ***Basidiomycota*** | 41.39 | 40.79 | 73.18 | 82.57 | 87.88 | 32.02 | 29.58 | 26.34 | 27.23 | 36.88 | 27.26 | 46.24 |
| ***Agaricomycetes*** | 27.18 | 13.90 | 6.74 | 2.22 | 1.52 | 13.45 | 18.53 | 16.16 | 14.83 | 10.62 | 14.04 | 8.14 |
| *Agaricales* | 7.05 | 2.25 | 1.90 | 0.96 | 0.55 | 4.00 | 3.03 | 4.45 | 2.60 | 2.46 | 3.74 | 1.84 |
| *Boletales* | 1.18 | * | * | - | - | * | * | * | * | * | * | * |
| *Phallales* | 1.50 | * | * | * | - | * | * | * | * | * | * | * |
| *Polyporales (Ganodermataceae)*[b] | 2.42 | 6.55 | 0.81 | * | * | 3.60 | 3.43 | 2.31 | 1.98 | 2.96 | 1.79 | 2.57 |
| *Russulales (Russulaceae)*[b] | 6.44 | * | * | * | * | * | 0.71 | 2.31 | 2.49 | * | 1.36 | * |
| *Thelephorales (Thelephoraceae)*[b] | 3.11 | 2.41 | 1.98 | 0.62 | * | 2.89 | 5.57 | 2.44 | 0.70 | 0.71 | 0.59 | 0.55 |
| *Trechisporales* | 1.39 | * | 0.80 | * | * | 1.22 | 1.42 | 0.80 | 2.46 | 2.58 | 2.80 | 1.89 |
| ***Microbotryomycetes*** | 1.18 | 2.94 | 1.13 | * | * | 2.42 | 1.40 | 1.01 | 1.49 | 2.57 | 1.65 | 2.04 |
| *Leucosporidiales* | 1.06 | 2.36 | 1.08 | * | * | 0.97 | 0.95 | 0.76 | 1.42 | 2.33 | 1.46 | 1.68 |
| *Sporidiobolales* | * | * | - | * | * | 1.23 | * | * | - | - | - | * |
| ***Tremellomycetes*** | 12.62 | 22.12 | 65.16 | 79.90 | 85.98 | 15.78 | 9.21 | 8.64 | 9.88 | 22.71 | 9.81 | 34.94 |
| *Cystofilobasidiales (Syzygospora)*[b] | 1.25 | 1.56 | 1.64 | * | * | 0.67 | 0.76 | 0.83 | 0.88 | 0.60 | * | 0.92 |
| *Tremellales (Cryptococcus)*[b] | 4.81 | 10.44 | 2.58 | 2.56 | 1.73 | 12.99 | 5.35 | 5.40 | 4.51 | 2.08 | 5.65 | 2.42 |
| *Trichosporonaceae (Trichosporon)*[b] | 6.46 | 9.84 | 60.85 | 77.14 | 84.05 | 1.98 | 2.95 | 2.36 | 4.44 | 19.89 | 3.65 | 31.36 |

| | **Substrate SIP experiment**[c] | | | | | | | | **pH shift SIP experiment**[d] | | | |
|---|---|---|---|---|---|---|---|---|---|---|---|---|
| | | | | | | | | | pH 4 | | pH 7 | |
| | **$t_0$** | **MeOH** | **Ace +** | **Glu +** | **Xyl +** | **Van +** | **$CO_2$ +** | **$CO_2$** | **$t_0$** | **MeOH** | **$t_0$** | **MeOH** |
| ***Chytridiomycota*** | ∗ | ∗ | 1.06 | ∗ | - | - | ∗ | ∗ | - | ∗ | ∗ | ∗ |
| ***Glomeromycota*** | ***0.52*** | ***2.03*** | ***1.18*** | ∗ | ***0.55*** | ***2.21*** | ***3.70*** | ***2.40*** | ***0.65*** | ***0.52*** | ∗ | ***0.62*** |
| ***Rozellomycota*** | 3.78 | 3.55 | 1.89 | ∗ | 1.07 | 1.10 | 1.73 | 1.94 | 2.70 | 3.04 | 2.42 | 2.97 |
| ***Zygomycota*** | 8.54 | 15.51 | 5.76 | 2.68 | 1.95 | 12.09 | 15.74 | 9.90 | 14.46 | 19.71 | 18.30 | 12.30 |
| *Mortierellales* *(Mortierellaceae)* [b] | 8.24 | 15.22 | 5.61 | 2.66 | 1.94 | 11.91 | 15.64 | 9.75 | 14.35 | 19.66 | 18.14 | 12.11 |
| **not affiliated** | 0.60 | 0.54 | ∗ | ∗ | ∗ | 0.68 | 1.33 | 1.58 | 1.16 | 0.92 | 0.87 | 0.84 |

[a] Taxonomic affiliation is based on the dynamic UNITE database (v7, release 01.08.2015) and was done with a bayesian classifier implied with MOTUHR based on the best hit of consensus taxonomy after 100 bootstrapped assignments (for further reference sequences based on 'massBLASTer' of UNITE see Table S5)

[b] Taxa in brackets dominated fungal order or family

[c] Treatment with methanol (MeOH), acetate (Ace), glucose (Glu), xylose (Xyl), vanillic acid (Van) and carbon dioxide ($CO_2$); cross (+) indicates additional methanol supplementation

[d] Treatment with methanol at different pH conditions (pH 4 and pH 7)

**Table S13. Similarity analyses of bacterial communities (family-level with 90.1% cut-off of 16S rRNA gene sequence) of both SIP experiments based on ANOSIM (Analysis of Similarity) and NPMANOVA (non-parametric multivariate analysis of variance).** Values of total analyses in bold, pairwaise analyses in cursive.

**ANOSIM R 0.75/ p 0.0001**

| | **Substrate SIP experiment[a] R 0.55 / p 0.001** | | | | | | | **pH SIP experiment[b] R 1.00 / p 0.02** | | **$t_0$ R 0.48 / p 0.06** | | |
|---|---|---|---|---|---|---|---|---|---|---|---|---|
| | MeOH | Ace + | Glu + | Xyl + | Van + | $CO_2$ + | $CO_2$ | pH4 | pH7 | Sub vs pH4[c] | Sub vs pH7[c] | pH4 vs pH7[c] |
| | $t_0$ vs $t_{End}$ | | | | | | | | | $t_0$ vs $t_0$ | | |
| *R* | *1.00* | *0.21* | *1.00* | *0.04* | *0.11* | *0.18* | *0.61* | *1.00* | *1.00* | *0.58* | *0.67* | *2.00* |
| | $t_{End\ MeOH}$ vs $t_{End\ Substrate}$ | | | | | | | $t_{End\ pH4}$ vs $t_{End\ pH7}$ | | | | |
| *R* | - | *1.00* | *0.50* | *1.00* | *1.00* | *1.00* | *0.50* | *1.00* | | | | |

**NPMANOVA F 8.23 / p 0.0001**

| | **Substrate SIP experiment[a] F 5.31 / p 0.0001** | | | | | | | **pH SIP experiment[b] F 19.71 / p 0.02** | | **$t_0$ F 1.75 / p 0.07** | | |
|---|---|---|---|---|---|---|---|---|---|---|---|---|
| | MeOH | Ace + | Glu + | Xyl + | Van + | $CO_2$ + | $CO_2$ | pH4 | pH7 | Sub vs pH4[c] | Sub vs pH7[c] | pH4 vs pH7[c] |
| | $t_0$ vs $t_{End}$ | | | | | | | | | $t_0$ vs $t_0$ | | |
| *F* | *6.31* | *3.63* | *6.85* | *1.66* | *1.90* | *2.80* | *3.57* | *262.50* | *8.94* | *2.02* | *1.98* | *28.69* |
| | $t_{End\ MeOH}$ vs $t_{End\ Substrate}$ | | | | | | | $t_{End\ pH4}$ vs $t_{End\ pH7}$ | | | | |
| *F* | - | *23.69* | *2.22* | *24.74* | *21.60* | *18.36* | *3.93* | *23.99* | | | | |

[a] Treatment with methanol (MeOH), acetate (Ace), glucose (Glu), xylose (Xyl), vanillic acid (Van) and carbon dioxide ($CO_2$); cross (+) indicates additional methanol supplementation

[b] Treatment with methanol at different pH conditions (pH 4 and pH 7)

[c] Comparison between $t_0$ of Substrate SIP experiment and pH-SIP experiment (Sub vs pH) and between both t0 of pH-SIP (pH4 vs pH7)

**Table S14. Similarity analyses of fungal communities (family-level with 97.0% cut-off of ITS gene sequence) of both SIP experiments based on ANOSIM (Analysis of Similarity) and NPMANOVA (non-parametric multivariate analysis of variance).** Values of total analyses in bold, pairwaise analyses in cursive.

**ANOSIM R 0.82 / p 0.0001**

| | **Substrate SIP experiment[a] R 0.78 / p 0.0001** | | | | | | | **pH SIP experiment[b] R 0.69 / p 0.07** | | **$t_0$ R 0.60 / p 0.33** | | |
|---|---|---|---|---|---|---|---|---|---|---|---|---|
| | MeOH | Ace + | Glu + | Xyl + | Van + | $CO_2$ + | $CO_2$ | pH4 | pH7 | Sub vs pH4[c] | Sub vs pH7[c] | pH4 vs pH7[c] |
| | $t_0$ vs $t_{End}$ | | | | | | | | | $t_0$ vs $t_0$ | | |
| *R* | *1.00* | *1.00* | *1.00* | *1.00* | *1.00* | *1.00* | *1.00* | *1.00* | *1.00* | *1.00* | *1.00* | *2.00* |
| | $t_{End\ MeOH}$ vs $t_{End\ Substrate}$ | | | | | | | $t_{End\ pH4}$ vs $t_{End\ pH7}$ | | | | |
| *R* | *-* | *1.00* | *1.00* | *1.00* | *0* | *0.75* | *1.00* | *0.00* | | | | |

**NPMANOVA F 8.11 / p 0.0001**

| | **Substrate SIP experiment[a] F 9.41 / p 0.0001** | | | | | | | **pH SIP experiment[b] F 2.98 / p 0.09** | | **$t_0$ F 8.21 / p 0.0001** | | |
|---|---|---|---|---|---|---|---|---|---|---|---|---|
| | MeOH | Ace + | Glu + | Xyl + | Van + | $CO_2$ + | $CO_2$ | pH4 | pH7 | Sub vs pH4[c] | Sub vs pH7[c] | pH4 vs pH7[c] |
| | $t_0$ vs $t_{End}$ | | | | | | | | | $t_0$ vs $t_0$ | | |
| *F* | *7.23* | *13.17* | *24.93* | *30.91* | *3.58* | *3.64* | *2.69* | *2.95* | *4.62* | *3.39* | *3.79* | *0.99* |
| | $t_{End\ MeOH}$ vs $t_{End\ Substrate}$ | | | | | | | $t_{End\ pH4}$ vs $t_{End\ pH7}$ | | | | |
| *F* | *-* | *19.50* | *47.01* | *65.45* | *1.82* | *2.55* | *2.77* | *1.46* | | | | |

[a] Treatment with methanol (MeOH), acetate (Ace), glucose (Glu), xylose (Xyl), vanillic acid (Van) and carbon dioxide ($CO_2$); cross (+) indicates additional methanol supplementation

[b] Treatment with methanol at different pH conditions (pH 4 and pH 7)

[c] Comparison between $t_0$ of Substrate SIP experiment and pH-SIP experiment (Sub vs pH) and between both t0 of pH-SIP (pH4 vs pH7)

**Table S15. Similarity analyses of *mxaF*-possessing methylotrophic communities (90% cut-off) of both SIP experiments based on ANOSIM (Analysis of Similarity) and NPMANOVA (non-parametric multivariate analysis of variance).** Values of total analyses in bold, pairwaise analyses in cursive.

**ANOSIM  R 0.33 / p 0.02**

| | **Substrate SIP experiment[a]** **R 0.18 / p 0.13** | | | | | | | **pH SIP experiment[b]** **R 0.85 / p 0.02** | | **$t_0$** **R -0.04 / p 0.66** | | |
|---|---|---|---|---|---|---|---|---|---|---|---|---|
| | MeOH | Ace + | Glu + | Xyl + | Van + | $CO_2$ + | $CO_2$ | pH4 | pH7 | Sub vs pH4[c] | Sub vs pH7[c] | pH4 vs pH7[c] |
| | $t_0$ vs $t_{End}$ | | | | | | | | | $t_0$ vs $t_0$ | | |
| *R* | *0.14* | *0.14* | *0.11* | *0.14* | *-0.11* | *0.14* | *0.11* | *0.00* | *1.00* | *0.00* | *0.00* | *2.00* |
| | $t_{End\ MeOH}$ vs $t_{End\ Substrate}$ | | | | | | | $t_{End\ pH4}$ vs $t_{End\ pH7}$ | | | | |
| *R* | - | *0.25* | *0.25* | *1.00* | *0.25* | *-0.5* | *0.00* | *1.00* | | | | |

**NPMANOVA  F 2.02 / p 0.0023**

| | **Substrate SIP experiment[a]** **F 1.49 / p 0.07** | | | | | | | **pH SIP experiment[b]** **F 7.52 / p 0.04** | | **$t_0$** **F 1.59 / p 0.03** | | |
|---|---|---|---|---|---|---|---|---|---|---|---|---|
| | MeOH | Ace + | Glu + | Xyl + | Van + | $CO_2$ + | $CO_2$ | pH4 | pH7 | Sub vs pH4[c] | Sub vs pH7[c] | pH4 vs pH7[c] |
| | $t_0$ vs $t_{End}$ | | | | | | | | | $t_0$ vs $t_0$ | | |
| *F* | *1.30* | *1.47* | *1.63* | *2.01* | *0.90* | *1.56* | *1.53* | *0.99* | *7.19* | *0.77* | *1.50* | *1.97* |
| | $t_{End\ MeOH}$ vs $t_{End\ Substrate}$ | | | | | | | $t_{End\ pH4}$ vs $t_{End\ pH7}$ | | | | |
| *F* | - | *1.46* | *1.13* | *2.20* | *1.23* | *0.81* | *1.13* | *15.43* | | | | |

[a] Treatment with methanol (MeOH), acetate (Ace), glucose (Glu), xylose (Xyl), vanillic acid (Van) and carbon dioxide ($CO_2$); cross (+) indicates additional methanol supplementation

[b] Treatment with methanol at different pH conditions (pH 4 and pH 7)

[c] Comparison between $t_0$ of Substrate SIP experiment and pH-SIP experiment (Sub vs pH) and between both t0 of pH-SIP (pH4 vs pH7)

**Table S16. Relative abundance of bacterial taxa based on 16S rRNA gene sequences from combined pyrosequencing data sets of [$^{12}$C]- and [$^{13}C_u$]-substrate treatments of both SIP experiments.** Only taxa with a relative abundance ≥ 0.5% are listed, abundance < 0.5% is indicated by ∗, no presence is indicated by -. Percentages are always related to filtered datasets of 16S rRNA gene sequence of pyrosequencing amplicon libraries.

| | Substrate SIP experiment[c] | | | | | | | | pH shift SIP experiment[d] | | | |
|---|---|---|---|---|---|---|---|---|---|---|---|---|
| | | | | | | | | | pH 4 | | pH 7 | |
| | **$t_0$** | **MeOH** | **Ace +** | **Glu +** | **Xyl +** | **Van +** | **$CO_2$ +** | **$CO_2$** | **$t_0$** | **MeOH** | **$t_0$** | **MeOH** |
| **number of sequences** combined data sets & singletons removed | 11979 | 4405 | 10105 | 4744 | 8098 | 10145 | 4203 | 5023 | 3796 | 26042 | 4281 | 11188 |
| **Taxonomic affiliation [a]** | **Relative abundance [%]** | | | | | | | | | | | |
| ***Acidobacteria*** | 3.89 | 0.98 | 0.89 | 1.05 | 2.09 | 1.86 | 0.88 | 2.43 | 5.64 | ∗ | 8.36 | ∗ |
| | | | | | | | | | | | | |
| ***Actinobacteria*** | 47.14 | 19.25 | 63.29 | 8.79 | 57.56 | 57.38 | 62.62 | 30.38 | 47.63 | 2.49 | 26.98 | 8.13 |
| *Actinomycetales* | 7.89 | 1.98 | 2.11 | 4.22 | 2.84 | 3.44 | 5.19 | 11.09 | 25.66 | 0.89 | 14.48 | 1.47 |
| *Kineosporiaceae (Kineosporia. OTU 703)* [b] | 7.61 | 1.82 | 2.04 | 3.96 | 2.80 | 3.40 | 4.83 | 10.55 | 23.29 | 0.70 | 11.42 | 0.88 |
| *Corynebacteriales* | 33.32 | 15.12 | 59.72 | 0.51 | 52.14 | 51.10 | 54.15 | 10.23 | 1.24 | ∗ | ∗ | ∗ |
| *Corynebacteriaceae* (*Corynebacterium. OTU 748*) [b] | 32.62 | 14.71 | 59.31 | ∗ | 51.67 | 50.63 | 53.91 | 9.06 | - | - | - | - |
| *Mycobacteriaceae* (*Mycobacterium. OTU 750*) [b] | 0.63 | ∗ | ∗ | ∗ | ∗ | ∗ | ∗ | 1.17 | 1.24 | ∗ | ∗ | ∗ |
| *Micrococcales* | | | | | | | | | | | | |
| *Microbacteriaceae (Leifsonia. OTU 721)* [b] | ∗ | ∗ | ∗ | ∗ | ∗ | ∗ | ∗ | ∗ | ∗ | 0.78 | ∗ | 4.45 |
| *Streptosporangiales* | 0.99 | ∗ | ∗ | 0.53 | ∗ | ∗ | ∗ | 1.19 | 2.29 | ∗ | 1.40 | ∗ |
| *other Actinobacteria* | 12.39 | 3.70 | 3.27 | 6.91 | 5.08 | 5.81 | 7.71 | 18.12 | 41.07 | 1.33 | 21.16 | 2.57 |

| | Substrate SIP experiment[c] | | | | | | | | pH shift SIP experiment[d] | | | |
|---|---|---|---|---|---|---|---|---|---|---|---|---|
| | | | | | | | | | pH 4 | | pH 7 | |
| | $t_0$ | MeOH | Ace + | Glu + | Xyl + | Van + | $CO_2$ + | $CO_2$ | $t_0$ | MeOH | $t_0$ | MeOH |
| ***Bacteroidetes*** | ∗ | 13.62 | 5.31 | 19.50 | 3.42 | 3.13 | 4.21 | 14.57 | 3.29 | 9.15 | 13.38 | 62.34 |
| *Flavobacteriales* | - | - | - | - | - | - | - | - | - | - | ∗ | 18.25 |
| *Flavobacteriaceae (Chryseobacterium. OTU 1045)*[b] | - | - | - | - | - | - | - | - | - | - | ∗ | 17.31 |
| *Sphingobacteriales* | ∗ | 13.55 | 5.18 | 19.39 | 3.30 | 3.03 | 4.07 | 14.19 | 1.21 | 9.02 | 8.11 | 38.63 |
| *Chitinophagaceae (Ferruginibacter. OTU 1014)*[b] | - | ∗ | ∗ | ∗ | ∗ | ∗ | 0.57 | 2.29 | ∗ | - | ∗ | 12.14 |
| *Sphingobacteriaceae (Mucilaginibacter; OTU 1073)*[b] | - | 13.14 | 4.86 | 18.82 | 3.03 | 2.79 | 3.50 | 11.83 | 0.76 | 7.96 | 6.73 | 15.99 |
| *other Bacteroidetes* | ∗ | ∗ | ∗ | ∗ | ∗ | ∗ | ∗ | ∗ | 2.08 | ∗ | 5.21 | 5.13 |
| ***"Cand. Saccharibacteria"*** | 0.63 | ∗ | ∗ | ∗ | ∗ | ∗ | ∗ | ∗ | 0.61 | ∗ | 1.94 | ∗ |
| ***Chlamydiae*** | 0.86 | ∗ | ∗ | ∗ | ∗ | ∗ | - | ∗ | 0.74 | ∗ | 0.96 | ∗ |
| ***Firmicutes*** | 0.51 | 1.20 | ∗ | 0.53 | 0.68 | 0.78 | ∗ | 1.17 | 0.53 | ∗ | 0.93 | 1.04 |
| *Bacilli* | ∗ | ∗ | ∗ | ∗ | ∗ | ∗ | ∗ | ∗ | ∗ | ∗ | ∗ | 0.76 |
| ***Parcubacteria*** | ∗ | 3.50 | 4.46 | 0.84 | ∗ | ∗ | ∗ | ∗ | ∗ | ∗ | ∗ | - |
| ***Planctomycetes*** | 27.53 | 6.97 | 6.95 | 9.19 | 15.46 | 16.25 | 7.73 | 12.06 | 10.48 | ∗ | 12.01 | 3.12 |
| *Planctomycetia* | 16.13 | 4.31 | 4.74 | 5.35 | 10.40 | 11.21 | 4.71 | 7.76 | 7.03 | ∗ | 6.82 | 2.61 |

| | Substrate SIP experiment[c] | | | | | | | | pH shift SIP experiment[d] | | | |
|---|---|---|---|---|---|---|---|---|---|---|---|---|
| | | | | | | | | | pH 4 | | pH 7 | |
| | **$t_0$** | **MeOH** | **Ace +** | **Glu +** | **Xyl +** | **Van +** | **$CO_2$ +** | **$CO_2$** | **$t_0$** | **MeOH** | **$t_0$** | **MeOH** |
| ***Proteobacteria*** | 12.62 | 48.42 | 14.66 | 56.11 | 16.84 | 17.13 | 20.99 | 33.76 | 25.37 | 86.68 | 26.26 | 18.10 |
| ***Alphaproteobacteria*** | 10.03 | 8.58 | 4.50 | 4.93 | 5.01 | 4.77 | 4.43 | 6.67 | 18.97 | 2.61 | 17.75 | 2.61 |
| *Rhizobiales* | 6.09 | 6.61 | 1.92 | 3.35 | 3.87 | 3.59 | 2.78 | 4.38 | 11.01 | 2.03 | 8.25 | 0.92 |
| *Beijerinckiaceae* (*Methylovirgula. OTU 438*) [b] | 6.04 | 6.61 | 1.92 | 2.91 | 3.87 | 3.58 | 2.78 | 4.38 | 10.96 | 2.03 | 8.11 | 0.86 |
| *Rhodospirillales* | 2.59 | 0.54 | 1.16 | * | * | * | 0.64 | 1.39 | 4.37 | * | 4.51 | * |
| *Acetobacteraceae* | 0.98 | * | 1.03 | * | * | * | * | 0.86 | 2.00 | * | 2.27 | * |
| *Sphingomonadales* | 0.55 | * | * | 0.89 | * | * | * | * | 1.77 | * | 2.31 | 0.73 |
| *other Alphaproteobacteria* | 0.79 | 1.09 | 0.98 | * | * | * | 0.59 | * | 1.82 | * | 2.69 | 0.72 |
| ***Betaproteobacteria*** | * | * | * | 8.31 | 8.74 | 9.31 | * | * | 0.76 | * | 0.61 | 2.53 |
| *Burkholderiales* | * | - | * | 8.31 | 8.74 | 9.29 | * | * | 0.71 | * | * | 0.79 |
| *Burkholderiaceae* (*Burkholderia. OTU 361*) [b] | * | - | * | 8.26 | 8.74 | 9.29 | * | * | 0.55 | * | * | * |
| *Methylophilales* (*Methylophilus. OTU 358*) [b] | - | - | - | - | - | - | - | * | * | - | - | 1.73 |
| ***Gammaproteobacteria*** | 1.58 | 39.50 | 9.77 | 42.58 | 3.03 | 2.97 | 16.23 | 26.54 | 4.45 | 83.98 | 6.59 | 12.80 |
| *Xanthomonadales* | 0.60 | 37.25 | 9.61 | 42.22 | 2.69 | 2.63 | 15.92 | 25.24 | 3.53 | 83.70 | 4.18 | 12.27 |
| *Xanthomonadaceae* (*Rhodanobacter. OTU 300*) [b] | 0.58 | 37.09 | 9.45 | 42.12 | 2.62 | 2.57 | 15.92 | 25.20 | 3.45 | 83.65 | 4.09 | 12.16 |
| *other Gammaproteobacteria* | 0.98 | 2.25 | * | * | * | * | * | 0.72 | 0.87 | * | 2.29 | * |
| ***Deltaproteobacteria*** | 0.83 | * | * | * | * | * | * | * | 1.19 | * | 1.31 | * |

| | **Substrate SIP experiment**[c] | | | | | | | | **pH shift SIP experiment**[d] | | | |
|---|---|---|---|---|---|---|---|---|---|---|---|---|
| | | | | | | | | | pH 4 | | pH 7 | |
| | **$t_0$** | **MeOH** | **Ace +** | **Glu +** | **Xyl +** | **Van +** | **$CO_2$ +** | **$CO_2$** | **$t_0$** | **MeOH** | **$t_0$** | **MeOH** |
| ***Verrucomicrobia*** | 2.88 | 4.09 | 3.19 | 2.19 | 2.49 | 2.13 | 1.95 | 3.30 | 2.03 | 0.56 | 4.93 | 3.31 |
| *Methylacidiphilales* | ∗ | ∗ | ∗ | ∗ | ∗ | ∗ | ∗ | ∗ | - | ∗ | 0.56 | - |
| *Spartobacteria* | ∗ | 0.59 | 1.02 | ∗ | ∗ | ∗ | ∗ | 0.58 | ∗ | ∗ | 0.77 | 2.65 |
| *Verrucomicrobiae* | 1.56 | 2.07 | 1.46 | 1.05 | 1.52 | 1.29 | 1.24 | 2.07 | 1.21 | ∗ | 3.01 | 0.50 |
| *other Verrucomicrobia* | ∗ | 1.18 | 0.50 | ∗ | 0.54 | ∗ | ∗ | ∗ | ∗ | ∗ | ∗ | ∗ |
| ***not affiliated bacteria*** | 3.19 | 1.04 | 0.75 | 1.01 | ∗ | ∗ | 0.67 | 1.08 | 2.48 | ∗ | 3.22 | 3.36 |

[a] Taxonomic affiliation was done with JAguc2 and is based on GenBank Release (GenBank release 209, 14.08.2015)
[b] Genera in brackets dominated bacterial taxa
[c] Treatment with methanol (MeOH), acetate (Ace), glucose (Glu), xylose (Xyl), vanillic acid (Van) and carbon dioxide ($CO_2$); cross (+) indicates additional methanol supplementation
[d] Treatment with methanol at different pH conditions (pH 4 and pH 7)

**Table S17. Relative abundance of methylotrophic taxa (OTU) based on *mxaF* gene sequences from combined pyrosequencing data sets of [$^{12}$C]- and [$^{13}C_u$]-substrate treatments of both SIP experiments.** Only taxa with a relative abundance ≥ 0.5% are listed, abundance < 0.5% is indicated by ∗, no presence is indicated by -. Percentages are always related to filtered datasets of *mxaF* gene sequence of pyrosequencing amplicon libraries.

| | Substrate SIP experiment[c] | | | | | | | | pH shift SIP experiment[d] | | | |
|---|---|---|---|---|---|---|---|---|---|---|---|---|
| | | | | | | | | | pH 4 | | pH 7 | |
| | $t_0$ | MeOH | Ace + | Glu + | Xyl + | Van + | $CO_2$ + | $CO_2$ | $t_0$ | MeOH | $t_0$ | MeOH |
| **number of sequences** combined data sets & singletons removed | 13602 | 2036 | 6963 | 4604 | 7568 | 5372 | 4862 | 3004 | 8364 | 16826 | 11167 | 29321 |
| **Taxonomic affiliation [a]** | **Relative abundance [%]** | | | | | | | | | | | |
| ***Methylobacteriaceae*** | 72.43 | 31.14 | 50.29 | 43.22 | 44.37 | 31.24 | 33.63 | 56.79 | 55.26 | 39.24 | 16.79 | 72.92 |
| OTU 35 | 60.23 | - | ∗ | ∗ | 1.10 | 19.62 | 1.25 | 1.76 | 50.12 | 35.88 | 8.08 | 2.56 |
| OTU 40 | 8.34 | 19.94 | 45.91 | 32.78 | 4.47 | 5.64 | 16.97 | 34.79 | 2.00 | 2.41 | 3.00 | 30.17 |
| OTU 55 | 2.98 | 9.63 | 2.11 | 7.84 | 36.87 | 3.80 | 13.00 | 18.28 | 3.13 | 0.90 | 5.71 | 39.61 |
| OTU 76 | ∗ | ∗ | ∗ | 0.52 | ∗ | ∗ | ∗ | ∗ | - | - | - | ∗ |
| OTU 78 | ∗ | ∗ | 0.56 | ∗ | 0.81 | 1.01 | 1.44 | 0.93 | - | - | - | ∗ |
| OTU 79 | ∗ | ∗ | ∗ | 0.52 | ∗ | ∗ | ∗ | 0.53 | - | ∗ | - | ∗ |
| OTU 107 | ∗ | ∗ | ∗ | 0.52 | ∗ | ∗ | ∗ | ∗ | ∗ | - | - | ∗ |
| OTU 108 | ∗ | ∗ | 0.95 | ∗ | ∗ | ∗ | ∗ | ∗ | - | ∗ | - | ∗ |
| ambiguous [b] | ∗ | 1.33 | 2.77 | ∗ | ∗ | ∗ | ∗ | ∗ | ∗ | ∗ | ∗ | 1.44 |
| OTU 9 | - | ∗ | 2.56 | - | ∗ | - | - | - | ∗ | ∗ | ∗ | - |
| OTU 141 | - | - | - | - | - | - | - | - | - | - | - | 1.39 |
| OTU 222 | ∗ | 1.03 | ∗ | ∗ | ∗ | ∗ | ∗ | ∗ | ∗ | - | - | ∗ |

| | Substrate SIP experiment[c] | | | | | | | | pH shift SIP experiment[d] | | | |
|---|---|---|---|---|---|---|---|---|---|---|---|---|
| | | | | | | | | | pH 4 | | pH 7 | |
| | $t_0$ | MeOH | Ace + | Glu + | Xyl + | Van + | $CO_2$ + | $CO_2$ | $t_0$ | MeOH | $t_0$ | MeOH |
| ***Hyphomicrobiaceae*** | 14.80 | 15.86 | 24.67 | 27.65 | 7.60 | 8.41 | 20.16 | 3.36 | 6.22 | 8.31 | 24.89 | 10.85 |
| OTU 185 | 14.36 | 15.13 | 24.44 | 6.75 | 5.92 | 7.48 | 5.90 | 3.10 | 1.52 | 3.55 | 1.29 | 4.27 |
| OTU 202 | * | * | * | * | * | 0.76 | * | * | - | * | * | * |
| OTU 243 | * | * | * | * | * | - | * | - | * | 2.49 | * | * |
| OTU 257 | * | * | - | - | * | * | * | - | 3.32 | 1.36 | 22.78 | 6.36 |
| OTU 308 | * | - | - | - | * | - | - | - | 0.98 | * | * | * |
| OTU 309 | * | * | - | 20.79 | 1.15 | * | 14.01 | * | * | 0.56 | * | * |
| ambiguous [b] | 7.47 | 42.19 | 9.31 | 16.36 | 19.87 | 39.02 | 29.08 | 33.16 | 31.79 | 41.16 | 43.69 | 7.32 |
| OTU 172 | 0.82 | 3.88 | 0.69 | 3.78 | 8.31 | 3.11 | 4.44 | 3.36 | 3.71 | 3.34 | 3.73 | 1.48 |
| OTU 210 | * | 7.91 | 0.80 | 0.54 | 0.54 | 10.67 | 19.29 | * | 6.37 | 6.85 | 3.54 | 1.42 |
| OTU 214 | * | 4.86 | * | * | * | * | * | 1.33 | 2.61 | 1.86 | 14.56 | - |
| OTU 236 | * | * | 5.20 | 2.04 | 2.34 | 5.53 | 0.93 | 0.83 | 17.19 | 27.56 | 20.50 | 3.89 |
| OTU 266 | 5.65 | 24.90 | 0.85 | 9.30 | 2.48 | 14.09 | 2.45 | 9.15 | 1.26 | 0.58 | * | * |
| OTU 286 | * | - | * | - | - | * | * | 17.31 | - | - | - | - |
| OTU 298 | * | 0.54 | 1.61 | * | 6.01 | 5.32 | 1.56 | 1.00 | * | 0.75 | * | * |
| OTU 310 | - | - | - | - | - | - | - | - | * | * | 0.68 | * |
| *Methylorhabdus* | * | 0.54 | * | * | * | * | * | * | - | * | - | * |
| OTU 190 | * | 0.54 | * | * | * | * | * | * | - | * | - | * |
| ambiguous [b] | * | * | * | * | - | - | * | - | * | 2.81 | * | - |
| OTU 18 | * | * | * | * | - | - | * | - | * | 2.81 | * | - |
| ***Methylocystaceae*** | 0.57 | - | * | 2.74 | - | - | - | - | - | - | - | |
| OTU 137 | 0.57 | - | * | 2.74 | - | - | - | - | - | - | - | - |

| | Substrate SIP experiment[c] | | | | | | | | pH shift SIP experiment[d] | | | |
|---|---|---|---|---|---|---|---|---|---|---|---|---|
| | | | | | | | | | pH 4 | | pH 7 | |
| | $t_0$ | MeOH | Ace + | Glu + | Xyl + | Van + | $CO_2$ + | $CO_2$ | $t_0$ | MeOH | $t_0$ | MeOH |
| ***Beijerinckiaceae*** | * | 2.85 | * | * | * | 1.04 | 0.51 | - | * | * | 1.33 | 0.69 |
| OTU 144 | * | 2.85 | * | * | * | 1.04 | 0.51 | - | * | * | 1.33 | 0.69 |
| ambiguous [b] | * | * | 1.82 | 4.17 | 4.78 | 0.86 | 10.51 | 0.87 | 2.76 | 3.55 | 6.12 | 0.34 |
| OTU 338 | * | * | * | * | 1.03 | * | 0.58 | * | * | * | 0.55 | * |
| OTU 340 | * | - | 1.77 | 3.50 | 3.74 | * | * | 0.60 | 1.69 | 1.60 | 4.32 | * |
| OTU 349 | - | - | - | * | * | - | 9.79 | - | 0.62 | 1.47 | 1.25 | * |
| ***not affiliated*** | 1.53 | 0.54 | 4.87 | 0.76 | 15.86 | 11.93 | 0.76 | 0.80 | 2.28 | 2.29 | 5.01 | 3.91 |
| OTU 21 | 0.76 | - | 4.64 | - | 15.31 | 9.94 | * | - | 2.18 | 2.20 | 1.01 | * |
| OTU 90 | * | - | * | * | * | 0.74 | * | * | - | - | - | - |
| OTU 200 | * | 0.54 | * | * | * | * | * | * | - | - | - | * |
| OTU 234 | * | - | - | * | - | 0.54 | * | * | - | * | - | * |
| OTU 268 | * | - | * | * | * | 0.58 | * | * | - | - | - | - |
| OTU 282 | * | - | - | - | - | - | - | - | * | * | * | 2.58 |
| OTU 325 | - | - | - | - | * | - | - | - | * | * | 3.81 | 1.27 |

[a] Taxonomic affiliation was done with BLASTn (December 2015; for further information see Table S2) and confirmed by positioning in phylogenetic tree (data not shown)

[b] Sequence identity with BLASTn <90% as well as ambiguous position in phylogenetic tree (for further information see Table S2)

[c] Treatment with methanol (MeOH), acetate (Ace), glucose (Glu), xylose (Xyl), vanillic acid (Van) and carbon dioxide ($CO_2$); cross (+) indicates additional methanol supplementation

[d] Treatment with methanol at different pH conditions (pH 4 and pH 7)
